# Supplementary material for: Molecular basis for the reversible ADP-ribosylation of guanosine bases
Source: Mol Cell. 2023 Jul 6;83(13):2303–2315.e6. doi: 10.1016/j.molcel.2023.06.013 (PMC11543638; doi:10.1016/j.molcel.2023.06.013)
Supplement: Document S1. Figures S1–S6 and Tables S1–S4 [file mmc1.pdf]

Molecular Cell, Volume *83*

Supplemental information

Molecular basis for the reversible ADP-ribosylation of guanosine bases

Marion Schuller, Roberto Raggiaschi, Petra Mikolcevic, Johannes G.M. Rack, Antonio Ariza, YuGeng Zhang, Raphael Ledermann, Christoph Tang, Andreja Mikoc, and Ivan Ahel

Molecular basis for the reversible ADP-ribosylation of guanosine bases

Marion Schuller[1], Roberto Raggiaschi[1], Petra Mikolcevic[2], Johannes G. M. Rack[1], Antonio Ariza[3], YuGeng Zhang[1], Raphael Ledermann[4], Christoph Tang[1], Andreja Mikoc[2], Ivan Ahel[1*]

[1]*Sir William Dunn School of Pathology, University of Oxford, Oxford, United Kingdom*
[2]*Division of Molecular Biology, Ruđer Bošković Institute, Zagreb, Croatia*
[3]*School of Biosciences, University of Sheffield, Sheffield, United Kingdom*
[4]*Department of Biology, University of Oxford, Oxford, United Kingdom*

*Corresponding author and lead contact: ivan.ahel@path.ox.ac.uk

SUPPLEMENTAL INFORMATION

SUPPLEMENTARY FIGURES

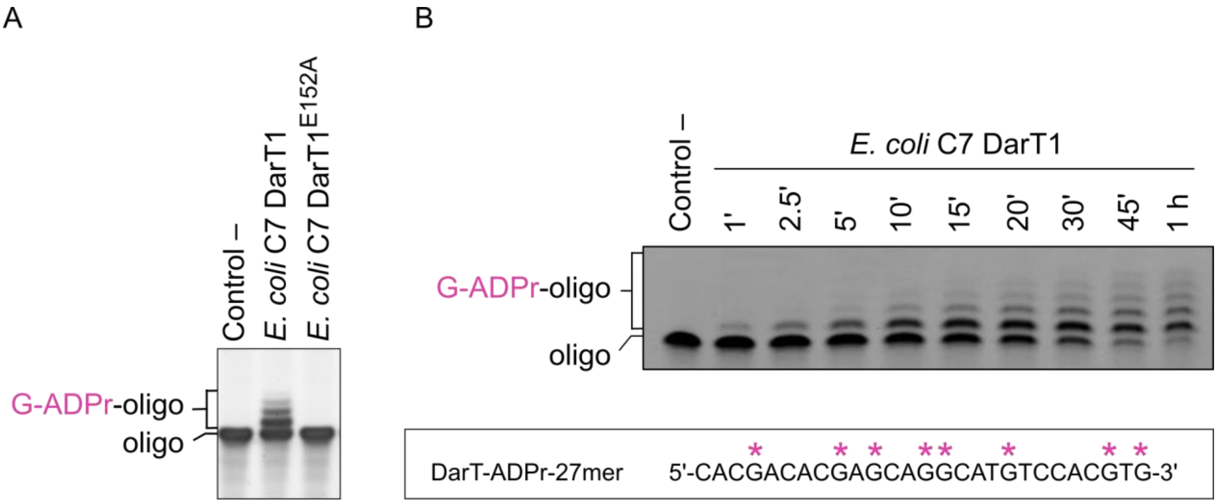

Supplementary Figure S1. ADP-ribosylation activity of *E. coli* C7 DarT1, related to Figure 2. (A) *In vitro* ADP-ribosylation activity of *E. coli* C7 DarT1 wild-type compared to mutant on the ssDNA substrate “DarT-ADPr-27mer”. Several distinct shifts of modified oligo compared to the unmodified oligo can be visualised, indicating the presence of multiple ADPr modifications on the substrate. Representative of three independent experiments. **(B)** *In vitro* ADP-ribosylation activity of *E. coli* C7 DarT1 on the substrate “DarT-ADPr-27mer” over a time course of 1h. Several ADP-ribose modifications are added onto the substrate over time leading to a laddering effect. Representative for three independent experiments.

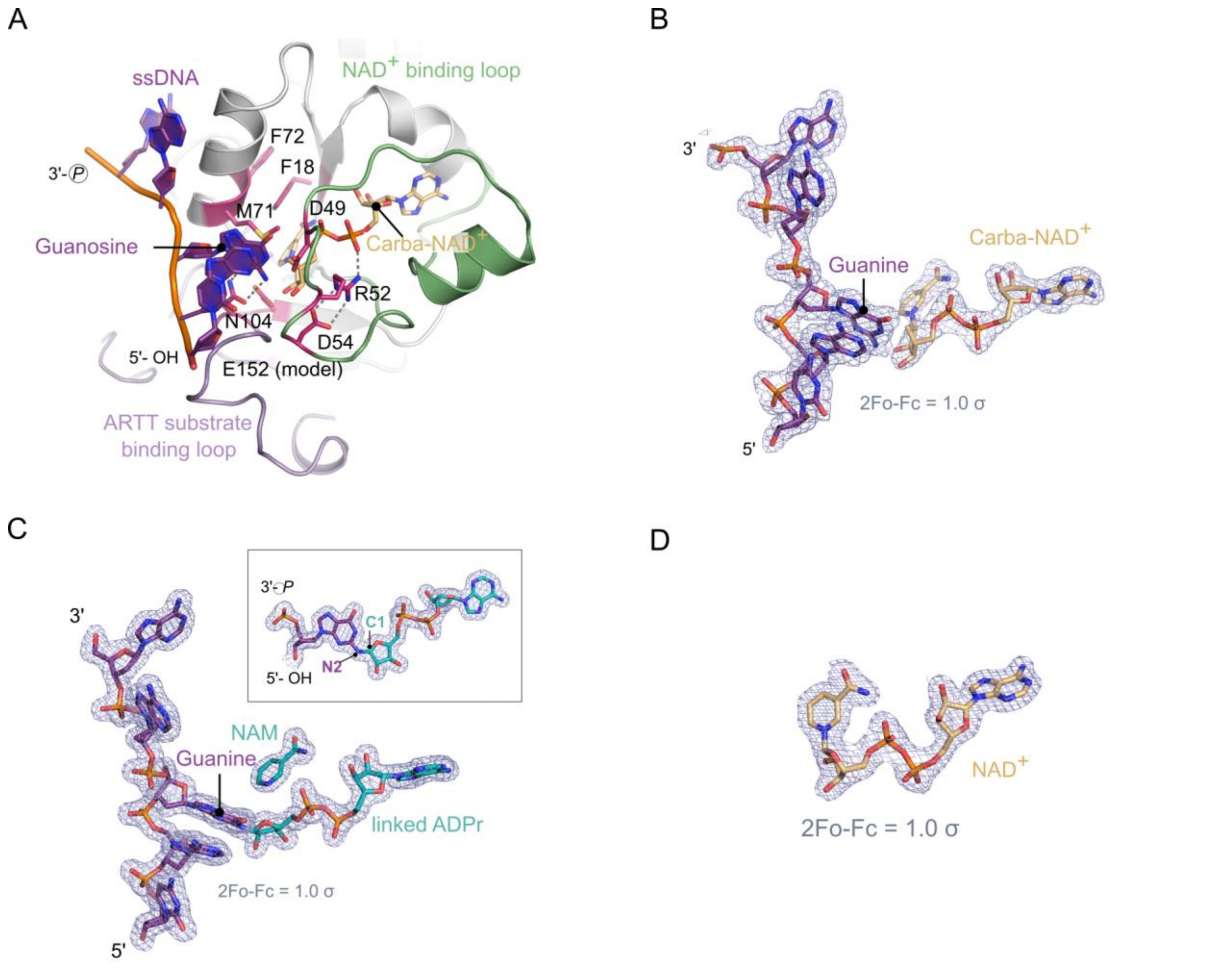

Supplementary Figure S2. DarT1 in substrate-bound pre-and post-reaction states, related to Figure 3. (A) Cartoon-stick model of the co-crystal structure of *E. coli* C7 DarT1^{E152A} with carba-NAD$^+$ (brown sticks) and ssDNA (sequence AAGAC). The substrate-binding ARTT loop is highlighted in purple, and the NAD$^+$-binding loop is in green. Active site residues are shown as pink sticks. **(B)** The 2Fo-Fc electron density map contoured at 1.0 σ around the ssDNA and the carba-NAD$^+$ ligand as in the structure shown in (A) is displayed in grey. **(C)** The 2Fo-Fc electron density map contoured at 1.0 σ around the ADP-ribosylated DNA and the NAM ligand as in *E. coli* C7 DarT1^{E152A} co-crystallised with NAD$^+$ and DNA is displayed in grey. The resolution of 1.63 Å allows revealing the DarT1-established connection of the distal-ribose C1 atom to the guanine N2 atom. The rectangular inset shows an enlarged view of this ADPr – DNA linkage. **(D)** The 2Fo-Fc electron density map contoured at 1.0 σ around the NAD$^+$ ligand as in the DarT1-NAD$^+$ co-crystal structure is shown in grey.

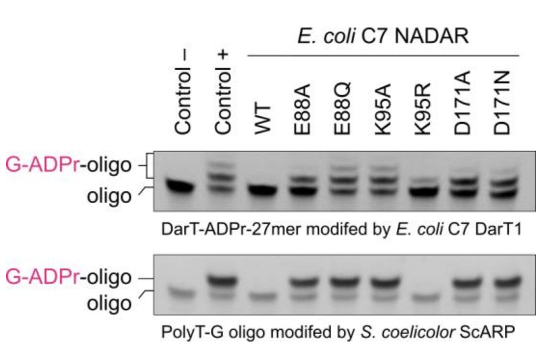

Supplementary Figure S3. Characterisation of catalytic residues of *E. coli* C7 NADAR, related to Figure 5. *In vitro* guanine-ADPr hydrolytic activity of *E. coli* C7 NADAR wild-type compared to catalytic mutants on the ssDNA substrates "DarT-ADPr-27mer" (modified by *E. coli* C7 DarT1) and "PolyT-G" (modified by *S. coelicolor* ScARP). Note, the de-ADP-ribosylation reaction of NADAR enzymes was only allowed to proceed for 15 min. Representative for three independent experiments.

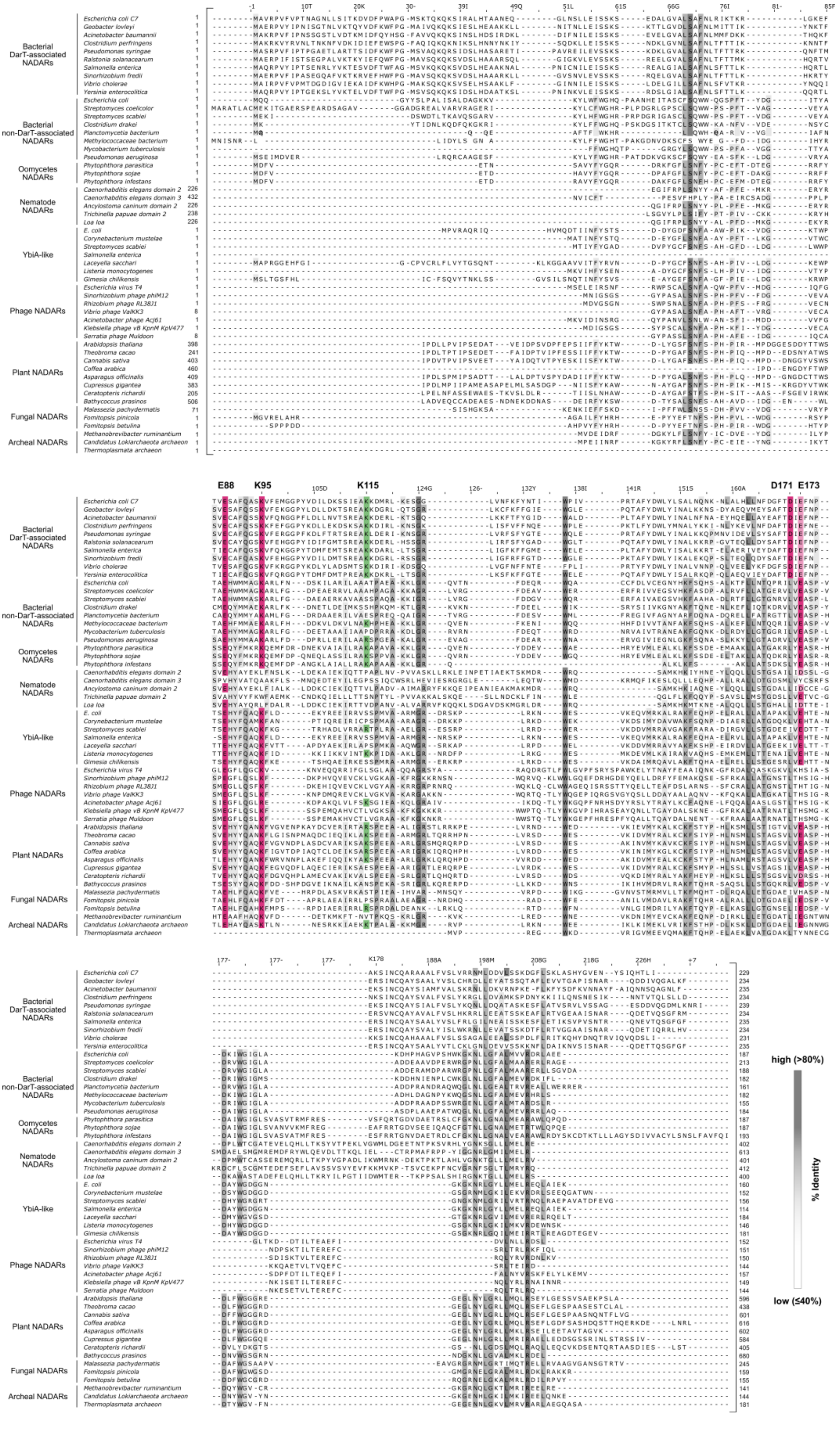

Supplementary Figure S4. Multiple sequence alignment of selected members of the NADAR superfamily, related to Figures 1 and 5. Catalytically relevant residues in *E. coli* C7 NADAR are highlighted in pink, and residues specific for DarT1-associated NADARs are highlighted in green. The alignment was used to construct the phylogenetic tree shown in Figure 1E.

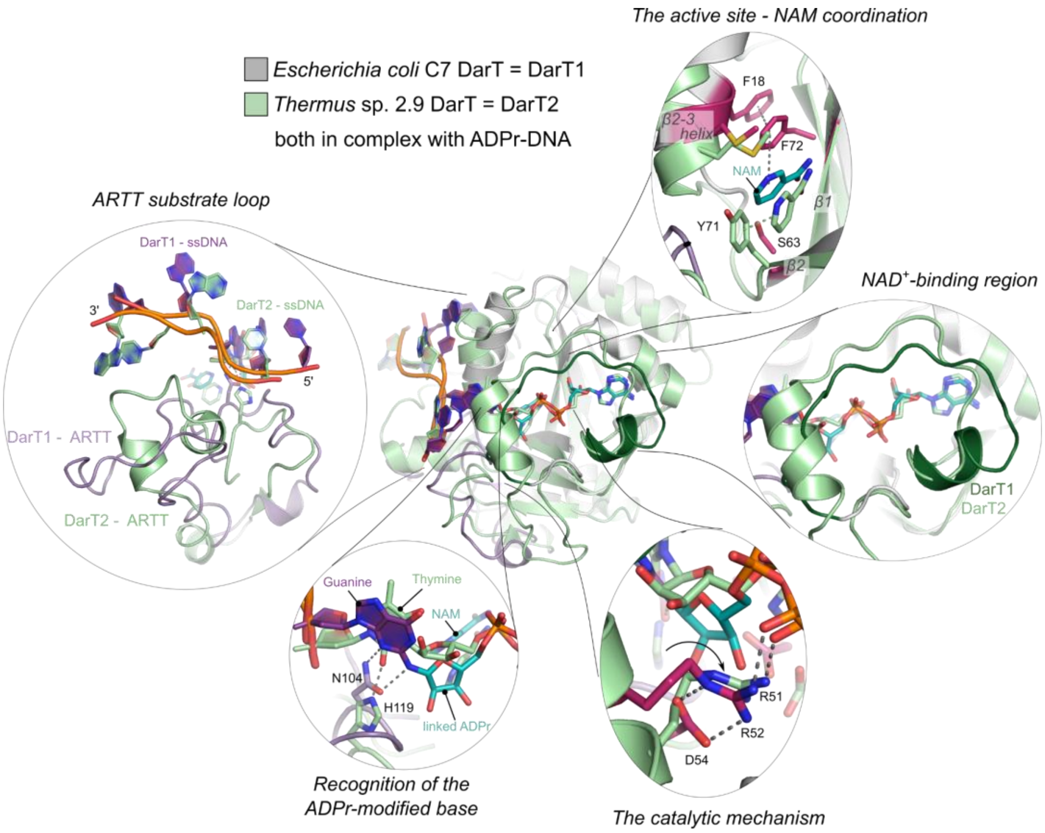

Supplementary Figure S5. Structural comparison of DarT1 and DarT2, related to Figure 3. In the middle of the figure, an overlay of *E. coli* C7 DarT, i.e. DarT1, with *Thermus* sp. 2.9 DarT, i.e. DarT2, both in complex with ADPr-DNA is shown. Differences in structural elements influencing the different catalytic functions are highlighted in the surrounding panels. The R51 side-chain flip observed in DarT2 compared to the pre-reaction state (not shown) is indicated by the black arrow. NAM coordination is relevant for considering NAD^+ polarisation.

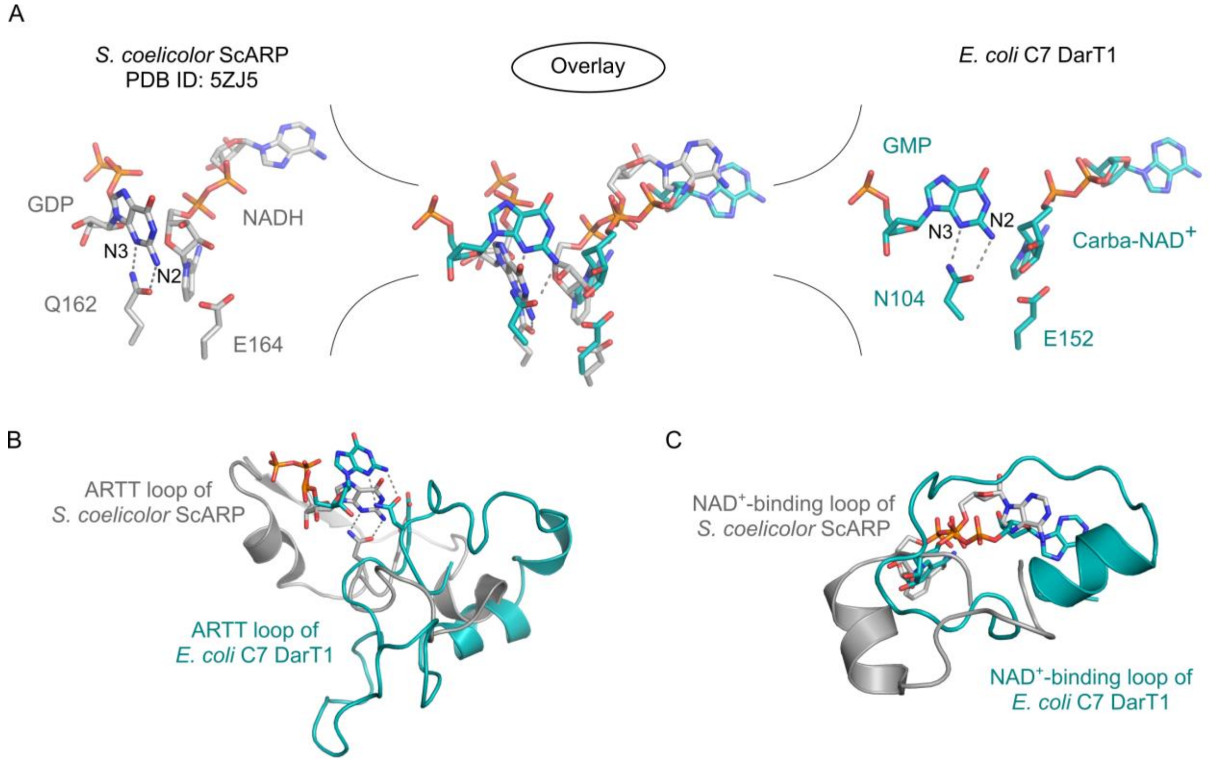

Supplementary Figure S6. Structural comparison of DarT1 and ScARP, related to Figure 6. (A) The co-crystal structure of *S. coelicolor* ScARP with GDP and NADH (PDB 5ZJ5) was overlaid with the *E. coli* C7 DarT1 structure in complex with GMP and carba-NAD$^+$. The NAD$^+$ derivatives and guanosine substrates take spatially similar positions although the transferases differ in their overall structural makeup, in particular regarding the ARTT substrate recognition loop **(B)** and the NAD$^+$-binding loop **(C)**. ScARP and DarT1 share the way of guanine positioning through N2 and N3 recognition and the orientation of the transferase-characteristic glutamate with respect to the distal-ribose of the NAD$^+$-derived ligand.

SUPPLEMENTARY TABLES

Supplementary Table S1. Data collection and refinement statistics for crystal structures described in this study, related to Figures 3, 4 and S2.

	E. coli C7 **DarT1: NAD$^+$**	***E. coli C7*** **DarT1: ADP-ribosylated DNA**	***E. coli*** **C7 DarT1: Carba-NAD$^+$ and DNA**	***G. lovleyi*** **NADAR apo**
**PDB accession code**	**8BAQ**	**8BAR**	**8BAS**	**8BAT**
**Data Collection**				
Synchrotron/beam line	DLS/I03	DLS/I03	DLS/I03	DLS/I03
Wavelength (Å)	0.9763	0.9763	0.9763	0.9763
Space group	$P\ 3_1\ 2\ 1$	$P\ 4_3\ 2_1\ 2$	$P\ 4_3\ 2_1\ 2$	$P\ 2_1\ 2\ 2_1$
a (Å)	62.31	61.76	61.90	39.28
b (Å)	62.31	61.76	61.90	81.60
c (Å)	113,08	215.04	215.55	86.63
α (°)	90.00	90.00	90.00	90.00
β (°)	90.00	90.00	90.00	90.00
γ (°)	120.00	90.00	90.00	90.00
Content of AU	1	1	1	1
Resolution (Å) [a]	53.96 - 2.00 (2.05 - 2.00)	61.74 - 1.63 (1.66 - 1.63)	61.90 – 1.92 (1.97 – 1.92)	59.40 – 2.30 (2.38 – 2.30)
R_{sym} (%) [a,b]	12.9 (140.4)	7.2 (158.1)	15.9 (236.3)	19.1 (147.5)
I/σ(I)	5.8 (0.7)	14.0 (1.3)	8.3 (0.9)	6.2 (1.0)
Completeness (%) [a]	99.9 (99.9)	100.0 (100.0)	100.0 (100.0)	100.0 (100.0)
Redundancy [a]	4.4 (4.4)	9.0 (9.0)	9.4 (9.8)	5.6 (5.3)
$CC_{1/2}$ (%) [a]	99.7 (61.5)	99.9 (70.1)	99.8 (50.1)	99.4 (55.7)
Unique reflections [a]	17764 (1267)	53204 (2567)	33191 (2151)	12984 (1242)
**Refinement**				
R_{cryst} (%) [c]	19.6	15.5	17.0	18.1
R_{free} (%) [d]	24.8	17.8	21.0	23.2
RMSD bond length (Å)	0.0043	0.012	0.013	0.011
RMSD bond angle (°)	1.171	1.89	2.04	1.95
Amino acids [e]	208 [47.6]	1721 [28.5]	1716 [39.1]	1761 [46.9]
Water [e]	131 [47.21]	356 [44.7]	242 [47.9]	124 [46.1]
Ligands [e]	5 [47.3]	173 [30.90]	177 [49.3]	4 [72.8]
Ions [e]	-	-	-	1 [49.7]
**Ramachandran plot**				
Favoured (%)	95.6	97.6	97.1	98.6
Allowed (%)	3.9	1.9	2,4	1.4
Disallowed (%)	0.5	0.5	0.5	0.0

(a) Data for the highest resolution shell are given in parentheses.

(b) R_{sym} = Σ|/-</>|/Σ/, where / is measured density for reflections with indices *hkl*.

(c) R_{cryst} = Σ||Fobs| - |Fcalc||/Σ|Fobs|.

(d) R_{free} has the same formula as R_{cryst}, except that calculation was made with the structure factors from the test set.

(e) Number of atoms followed the average B factor in brackets.

Supplementary Table S1. Data collection and refinement statistics for crystal structures described in this study, related to Figures 3, 4 and S2. (Continuation)

	***P. nicotianae var. parasitica* NADAR: ADPr**
**PDB accession code**	**8BAU**
**Data Collection**	
Synchrotron/beam line	DLS/I03
Wavelength (Å)	0.9763
Space group	$P\ 2_1\ 2_1\ 2_1$
a (Å)	44.83
b (Å)	66.59
c (Å)	72.89
α (°)	90.00
β (°)	90.00
γ (°)	90.00
Content of AU	1
Resolution (Å) [a]	49.17 - 1.60 (1.63 - 1.60)
R_{sym} (%) [a,b]	7.2 (212.8)
I/σ(I)	13.0 (0.9)
Completeness (%) [a]	100.0 (99.9)
Redundancy [a]	9.6 (9.8)
$CC_{1/2}$ (%) [a]	100.0 (59.6)
Unique reflections [a]	29538 (1421)
**Refinement**	
R_{cryst} (%) [c]	19.8
R_{free} (%) [d]	22.8
RMSD bond length (Å)	0.014
RMSD bond angle (°)	1.91
Amino acids	1539 [34.4]
Water	88 [36.6]
Ligands	56 [35.4]
Ions	-
**Ramachandran plot**	
Favoured (%)	98.4
Allowed (%)	1.6
Disallowed (%)	0.0

Supplementary Table S2. NCBI accession IDs of NADAR sequences used in this study, related to Figure 1.

Species	Accession number
**Bacterial NADARs (DarT-associated)**	
Escherichia coli C7	WP_032219797.1
Geobacter lovleyi [Trichlorobacter lovleyi]	WP_012470628.1
Sinorhizobium fredii	WP_014330845.1
Vibrio cholerae	WP_172778105.1
Acinetobacter baumannii	WP_001129309.1
Clostridium perfringens	MBS5923337.1
Yersinia enterocolitica	MBX9495195.1
Pseudomonas syringae	MCF9004830.1
Ralstonia solanacearum	WP_201016325.1
**Bacterial NADARs (non-DarT-associated)**	
Escherichia coli	WP_001183948.1
Methylococcaceae bacterium	NOQ36470.1
Pseudomonas aeruginosa	WP_116806626.1
Planctomycetia bacterium	MBL8863927.1
Streptomyces caniscabiei	WP_179201999.1
Streptomyces coelicolor	BDD75137.1
Mycobacterium tuberculosis	CNF61934.1
Clostridium drakei	WP_032077447.1
**Bacterial YbiA-like**	
Listeria monocytogenes	HAA3934926.1
Gimesia chilikensis	QDT22974.1
Laceyella sacchari	AUS10489.1
Salmonella enterica	WP_140040215.1
Streptomyces scabiei	WP_086756638.1
Corynebacterium mustelae	WP_047262349.1
Escherichia coli	HAW8155414.1
**Oomycetes NADARs**	
Phytophthora sojae	XP_009516941.1
Phytophthora nicotianae var. parasitica	XP_008911034.1
Phytophthora infestans	KAF4136563.1
**Phage NADARs**	
Sinorhizobium phage phiM12	YP_009143184.1
Acinetobacter phage Acj61	YP_004009822.1
Rhizobium phage RL38J1	QGZ13929.1
Klebsiella phage vB KpnM KpV477	YP_009288818.1
Vibrio phage ValKK3	YP_009201294.1
Escherichia virus T4	NP_049816.1
Serratia phage Muldoon	YP_009883850.1
**Nematode NADARs**	
Caenorhabditis elegans	NP_498348.1
Ancylostoma caninum	RCN47812.1
Loa loa	XP_003139559.1
Trichinella papuae	KRZ69666.1
**Plant and fungal NADARs**	
Fomitopsis pinicola	EPS93933.1
Fomitopsis betulina	KAI0715550.1
Arabidopsis thaliana	VYS59636.1
Theobroma cacao	EOY09810.1
Cannabis sativa	KAF4360218.1

Coffea arabica	XP_027089562.1
Asparagus officinalis	XP_020243908.1
Cupressus gigantea	ATG70670.1
Ceratopteris richardii	KAH7432347.1
Bathycoccus prasinos	XP_007514772.1
Malassezia pachydermatis	XP_017990970.1
**Archaeal NADARs**	
Methanobrevibacter ruminantium	WP_012956765.1
Candidatus Lokiarchaeota archaeon	MBD3226909.1
Thermoplasmata archaeon	MBE6519942.1

Supplementary Table S3. Oligonucleotides used in this study, related to STAR methods.

Oligo-ID	Sequence (5'→3')	Purpose
DarT_cryst	AAGAC	Co-crystallisation with DarT1
DarT-ADPr-27mer	CACGACACGAGCAGGCATGTCCACGTG	ADP-ribosylation activity assay
DarT-ADPr-27mer-rc	CACGTGGACATGCCTGCTCGTGTCGTG	Reverse complement for ADP-ribosylation activity assay
PolyT-G	TTTTTTGTTTTTTTTTTTT	ADP-ribosylation activity assay
PolyT-GG	TTTTTTGGTTTTTTTTTTT	ADP-ribosylation activity assay
PolyT-GTG	TTTTTTGTGTTTTTTTTTT	ADP-ribosylation activity assay
PolyT-GTTG	TTTTTTGTTGTTTTTTTTT	ADP-ribosylation activity assay
PolyT-GTTTG	TTTTTTGTTTGTTTTTTTT	ADP-ribosylation activity assay
PolyT-GTTTTG	TTTTTTGTTTTGTTTTTTT	ADP-ribosylation activity assay
DarT-Substrate_Motif1	CACTACACTATCATTCACTACCACTATC	ADP-ribosylation activity assay
DarT_Substrate_Motif2	CACTACACTATCATTCACGACCACTATC	ADP-ribosylation activity assay
DarT_Substrate_Motif3	CACTACACTATCATTCGAGCCCACTATC	ADP-ribosylation activity assay
DarT_Substrate_Motif4	CACTACACTATCATTCAGGCCCACTATC	ADP-ribosylation activity assay
DarT_Substrate_Motif5	CACTACACTATCATTCATGTCCACTATC	ADP-ribosylation activity assay
DarT_Substrate_Motif6	CACTACACTATCATTCACGTCCACTATC	ADP-ribosylation activity assay
DarT_Substrate_Motif7	CACTACACTATCATTCGTGTCCACTATC	ADP-ribosylation activity assay
EcoliDarT_fwd	AGAACCTGTACTTCCAATCCATGACCATCCAAGAAATTATTC	Cloning of *E. coli* DarT1 into pBAD33 expression vector by Gibson Assembly
EcoliDarT_rev	CCGCCAAAACAGCCAAGCTTTCAACCCAGATAATAATGAC	
GeoDarT_fwd	AGAACCTGTACTTCCAATCCATGCGTACCGCAGTTGAAAATC	Cloning of *G. lovleyi* DarT1 into pBAD33 expression vector by Gibson Assembly
GeoDarT_rev	CCGCCAAAACAGCCAAGCTTTCACAGCTGAAAGCTATTTGC	
pBAD33_fwd	AAGCTTGGCTGTTTTGGC	Vector amplification for Gibson Assembly
pBAD33_rev	GGATTGGAAGTACAGGTTC	
EcoliDarT-A152E-f001	CAGGCAGAGATTCTGGTGTTTGAGAAAATTCCGCCTAGCT	Mutagenesis of *E. coli* DarT1 to WT

EcoliDarT-A152E-r001	CAGAATCTCTGCCTGAACATCGGTGGTATATTCGCTCGGC	Mutagenesis of *E. coli* DarT1 to WT
GeoDarT-A152E-f001	CAGGCAGAGGTTCTGGTTTTTGGCACCATTGAACCGGCAT	Mutagenesis of *E. coli* DarT1 to WT
GeoDarT-A152E-r001	CAGAACCTCTGCCTGCGGATGCGTCGGATAACTACGCGGA	Mutagenesis of *E. coli* DarT1 to WT
EcoliDarT-F18A-f001	TTTCATGCGACCCATAGCGATAATCTGACCAGCATTCTGG	Mutagenesis of *E. coli* DarT1 to WT
EcoliDarT-F18A-r001	ATGGGTCGCATGAAACAGGCTGCGAATATTACGCTGCTGA	Mutagenesis of *E. coli* DarT1 to WT
EcoliDarT-D49A-f001	TGCAACGCGGAGGAACGCATTGATGGTCATCCTGATGCAA	Mutagenesis of *E. coli* DarT1
EcoliDarT-D49A-r001	TTCCTCCGCGTTGCAGTTATATTCATTGTTTTCGTTATCC	Mutagenesis of *E. coli* DarT1
EcoliDarT-R52A-f001	GAGGAAGCGATTGATGGTCATCCTGATGCAATTTGTCTGA	Mutagenesis of *E. coli* DarT1
EcoliDarT-R52A-r001	ATCAATCGCTTCCTCGTCGTTGCAGTTATATTCATTGTTT	Mutagenesis of *E. coli* DarT1
EcoliDarT-S63A-f001	TGTCTGGCGGTTAGCTATCCGAATGCCAAAATGTTTTACA	Mutagenesis of *E. coli* DarT1
EcoliDarT-S63A-r001	GCTAACCGCCAGACAAATTGCATCAGGATGACCATCAATG	Mutagenesis of *E. coli* DarT1
EcoliDarT-M71A-f001	GCCAAAGCGTTTTACAAATACCGCTGTCTGAAACCTGGTG	Mutagenesis of *E. coli* DarT1
EcoliDarT-M71A-r001	GTAAAACGCTTTGGCATTCGGATAGCTAACGCTCAGACAA	Mutagenesis of *E. coli* DarT1
EcoliDarT-F72A-f001	AAAATGGCGTACAAATACCGCTGTCTGAAACCTGGTGATT	Mutagenesis of *E. coli* DarT1
EcoliDarT-F72A-r001	TTTGTACGCCATTTTGGCATTCGGATAGCTAACGCTCAGA	Mutagenesis of *E. coli* DarT1
EcoliDarT-N104A-f001	CCGACCGCGGCAGCCAGCAATAATGTGCGTTTTATCAATC	Mutagenesis of *E. coli* DarT1
EcoliDarT-N104A-r001	GGCTGCCGCGGTCGGATAAAATGCACAATCTTTTGCCCAC	Mutagenesis of *E. coli* DarT1
EcoliDarT-D54A-f001	CGCATTGCGGGTCATCCTGATGCAATTTGTCTGAGCGTTAG	Mutagenesis of *E. coli* DarT1
EcoliDarT-D54A-r001	ATGACCCGCAATGCGTTCCTCGTCGTTGCAGTTATATTCA	Mutagenesis of *E. coli* DarT1
PpaNADAR_GTWY_for	GGGGACAAGTTTGTACAAAAAAGCAGGCTTCCTGGAAGTTCTG TTCCAGGGTCCGATGGACTTTGTGGAGACGAATTCTGCCG	Cloning Phytophthora NADAR
PpaNADAR_GTWY_rev	GGGGACCACTTTGTACAAGAAAGCT GGGTATTAGTCCTGTGGCTGTAACCAAGCGCG	
NADAR-EcoliC7-E88A-f001	ACCGTTGCGAGCGCGTTTCAAGCGAGCAAAGTGTTCGAAA	Mutagenesis of *E. coli* NADAR
NADAR-EcoliC7-E88A-r001	CGCGCTCGCAACGGTGAACTCCTTGCCCAGACGTTTGGTC	Mutagenesis of *E. coli* NADAR
NADAR-EcoliC7-K95A-f001	GCGAGCGCGGTGTTCGAAATGGGTGGCCCGTACGTTGACA	Mutagenesis of *E. coli* NADAR
NADAR-EcoliC7-K95A-r001	GAACACCGCGCTCGCTTGAAACGCGCTTTCAACGGTGAAC	Mutagenesis of *E. coli* NADAR
NADAR-EcoliC7-K115A-f001	GAGGCGGCGAAAGACATGCGTCTGAAGGAAAGCGGTGGCC	Mutagenesis of *E. coli* NADAR
NADAR-EcoliC7-K115A-r001	GTCTTTCGCCGCCTCAATGCTGCTTTTATCCAGGATGTCA	Mutagenesis of *E. coli* NADAR
NADAR-EcoliC7-K116A-f001	GCGAAGGCGGACATGCGTCTGAAGGAAAGCGGTGGCCTGG	Mutagenesis of *E. coli* NADAR
NADAR-EcoliC7-K116A-r001	CATGTCCGCCTTCGCCTCAATGCTGCTTTTATCCAGGATG	Mutagenesis of *E. coli* NADAR
NADAR-EcoliC7-R119A-f001	GACATGGCGCTGAAGGAAAGCGGTGGCCTGGTGAACTTCA	Mutagenesis of *E. coli* NADAR
NADAR-EcoliC7-R119A-r001	CTTCAGCGCCATGTCTTTCTTCGCCTCAATGCTGCTTTTA	Mutagenesis of *E. coli* NADAR
NADAR-EcoliC7-K121A-f001	CGTCTGGCGGAAAGCGGTGGCCTGGTGAACTTCAAATTTT	Mutagenesis of *E. coli* NADAR
NADAR-EcoliC7-K121A-r001	GCTTTCCGCCAGACGCATGTCTTTCTTCGCCTCAATGCTG	Mutagenesis of *E. coli* NADAR

NADAR-EcoliC7-D171A-f001	TTTACCGCGATCGAGTTTAACCCGGCGAAAAGCATTAACT	Mutagenesis of *E. coli* NADAR
NADAR-EcoliC7-D171A-r001	CTCGATCGCGGTAAAGCCGTCGAAGTTCAGCAGGTGCAGC	Mutagenesis of *E. coli* NADAR
NADAR-EcoliC7-E173A-f001	GATATCGCGTTTAACCCGGCGAAAAGCATTAACTGCCAAG	Mutagenesis of *E. coli* NADAR
NADAR-EcoliC7-E173A-r001	GTTAAACGCGATATCGGTAAAGCCGTCGAAGTTCAGCAGG	Mutagenesis of *E. coli* NADAR
NADAR-EcoliC7-K178A-f001	CCGGCGGCGAGCATTAACTGCCAAGCGCGTGCGGCGGCGC	Mutagenesis of *E. coli* NADAR
NADAR-EcoliC7-K178A-r001	AATGCTCGCCGCCGGGTTAAACTCGATATCGGTAAAGCCG	Mutagenesis of *E. coli* NADAR
NADAR-EcoliC7-D171N-f001	TTTACCAATATCGAGTTTAACCCGGCGAAAAGCATTAACT	Mutagenesis of *E. coli* NADAR
NADAR-EcoliC7-D171N-r001	CTCGATATTGGTAAAGCCGTCGAAGTTCAGCAGGTGCAGC	Mutagenesis of *E. coli* NADAR
NADAR-EcoliC7-E88Q-f001	ACCGTTCAGAGCGCGTTTCAAGCGAGCAAAGTGTTCGAAA	Mutagenesis of *E. coli* NADAR
NADAR-EcoliC7-E88Q-r001	CGCGCTCTGAACGGTGAACTCCTTGCCCAGACGTTTGGTC	Mutagenesis of *E. coli* NADAR
NADAR-EcoliC7-K95R-f001	GCGAGCCGCGTGTTCGAAATGGGTGGCCCGTACGTTGACA	Mutagenesis of *E. coli* NADAR
NADAR-EcoliC7-K95R-r001	GAACACGCGGCTCGCTTGAAACGCGCTTTCAACGGTGAAC	Mutagenesis of *E. coli* NADAR

Supplementary Table S4. Strains and plasmids used in this study, related to STAR methods.

Strain or plasmid-ID	Description	Source
DH5α	*huA2 a(argF-lacZ)U169 phoA glnV44 a80a(lacZ)M15 gyrA96 recA1 relA1 endA1 thi-1 hsdR17*	NEB
DH5α-macro	DH5α with integrated *T. aquaticus* DarG macrodomain at P21 site	Schuller *et al.*, 2021 [S1]
BL21	*fhuA2 [lon] ompT gal [dcm] ΔhsdS*	NEB
BL21(DE3)	*fhuA2 [lon] ompT gal (λ DE3) [dcm] ΔhsdS λ DE3 = λ sBamHIo ΔEcoRI-B int::(lacI::PlacUV5::T7 gene1) i21 Δnin5*	NEB
Rosetta™ BL21 (DE3)	*F-ompT hsdSB(rB- mB-) gal dcm (DE3) pRARE* (cam^R)	Novagen
**Plasmids**		
pBAD33	Medium copy plasmid with an arabinose-inducible promoter; cam^R	Guzman *et al.*, 1995 [S2]
pET28a	Medium copy plasmid containing the IPTG-inducible promoter; kan^R	Novagen
pNIC28-Bsa4	Medium copy plasmid containing the IPTG-inducible promoter; kan^R	Addgene [S3]
pBAD33_Taq_*darT*	pBAD33 carrying *T. aquaticus darT* full-length; cam^R	Jankevicius *et al.*, 2017 [S4]
pET28_SC_*SCO5461*	pET28a carrying *S. coelicolor scarp (SCO5461)* full-length; kan^R	Lalić, J. *et al.*, 2016 [S5]
pET28_Taq_*darG*_macro	pET28a carrying *T. aquaticus darG* macrodomain (aa 1-155); kan^R	Jankevicius *et al.*, 2017 [S4]
pBAD33_Ecoli_*darT1*	pBAD33 carrying *E. coli* C7 *darT1* full-length; cam^R	This study
pBAD33_Ecoli_*darT1*^{F18A}	pBAD33 carrying *E. coli* C7 *darT1*^{F18A} full-length; cam^R	This study
pBAD33_Ecoli_*darT1*^{D49A}	pBAD33 carrying *E. coli* C7 *darT1*^{D49A} full-length; cam^R	This study
pBAD33_Ecoli_*darT1*^{R52A}	pBAD33 carrying *E. coli* C7 *darT1*^{R52A} full-length; cam^R	This study
pBAD33_Ecoli_*darT1*^{D54A}	pBAD33 carrying *E. coli* C7 *darT1*^{D54A} full-length; cam^R	This study
pBAD33_Ecoli_*darT1*^{S63A}	pBAD33 carrying *E. coli* C7 *darT1*^{S63A} full-length; cam^R	This study
pBAD33_Ecoli_*darT1*^{M71A}	pBAD33 carrying *E. coli* C7 *darT1*^{M71A} full-length; cam^R	This study
pBAD33_Ecoli_*darT1*^{F72A}	pBAD33 carrying *E. coli* C7 *darT1*^{F72A} full-length; cam^R	This study
pBAD33_Ecoli_*darT1*^{N104A}	pBAD33 carrying *E. coli* C7 *darT1*^{N104A} full-length; cam^R	This study
pBAD33_Ecoli_*darT1*^{E152A}	pBAD33 carrying *E. coli* C7 *darT1*^{E152A} full-length; cam^R	This study
pNIC28_Ecoli_*darT*^{E152A}	pNIC28-Bsa4 carrying *E. coli C7 darT*^{E152A} full-length; kan^R	This study
pBAD33_Glov_*darT1*	pBAD33 carrying *G. lovleyi darT1* full-length; cam^R	This study
pBAD33_Glov_*darT1*^{E152A}	pBAD33 carrying *G. lovleyi darT1*^{E152A} full-length; cam^R	This study
pDEST17_Pnp_*nadar*	pDEST17 carrying *P. nicotianae* var. *parasitica nadar* full-length; kan^R	This study
pET28_Glov_*nadar*	pET28a carrying *G. lovleyi nadar* full-length; kan^R	This study
pET28_SinoR_*nadar*	pET28a carrying *S. fredii nadar* full-length; kan^R	This study
pET28_Ecoli_*nadar*	pET28a carrying *E. coli* C7 *nadar* full-length; kan^R	This study
pET28_Ecoli_*nadar*^{E88A}	pET28a carrying *E. coli* C7 *nadar*^{E88A} full-length; kan^R	This study
pET28_Ecoli_*nadar*^{E88Q}	pET28a carrying *E. coli* C7 *nadar*^{E88Q} full-length; kan^R	This study
pET28_Ecoli_*nadar*^{K95A}	pET28a carrying *E. coli* C7 *nadar*^{K95A} full-length; kan^R	This study
pET28_Ecoli_*nadar*^{K95R}	pET28a carrying *E. coli* C7 *nadar*^{K95R} full-length; kan^R	This study
pET28_Ecoli_*nadar*^{K115A}	pET28a carrying *E. coli* C7 *nadar*^{K115A} full-length; kan^R	This study
pET28_Ecoli_*nadar*^{K116A}	pET28a carrying *E. coli* C7 *nadar*^{K116A} full-length; kan^R	This study

pET28_Ecoli_*nadar*^{R119A}	pET28a carrying *E. coli* C7 *nadar*^{R119A} full-length; kan^{R}	This study
pET28_Ecoli_*nadar*^{K121A}	pET28a carrying *E. coli* C7 *nadar*^{K121A} full-length; kan^{R}	This study
pET28_Ecoli_*nadar*^{D171A}	pET28a carrying *E. coli* C7 *nadar*^{D171A} full-length; kan^{R}	This study
pET28_Ecoli_*nadar*^{D171N}	pET28a carrying *E. coli* C7 *nadar*^{D171N}full-length; kan^{R}	This study
pET28_Ecoli_*nadar*^{E173A}	pET28a carrying *E. coli* C7 *nadar*^{E173A} full-length; kan^{R}	This study
pET28_Ecoli_*nadar*^{K178A}	pET28a carrying *E. coli* C7 *nadar*^{K178A} full-length; kan^{R}	This study

SUPPLEMENTARY REFERENCES

S1. Schuller, M., Butler, R.E., Ariza, A., Tromans-Coia, C., Jankevicius, G., Claridge, T.D.W., Kendall, S.L., Goh, S., Stewart, G.R., and Ahel, I. (2021). Molecular basis for DarT ADP-ribosylation of a DNA base. Nature *596*, 597–602. 10.1038/s41586-021-03825-4.

S2. Guzman, L.M., Weiss, D.S., and Beckwith, J. (1997). Domain-swapping analysis of FtsI, FtsL, and FtsQ, bitopic membrane proteins essential for cell division in Escherichia coli. J Bacteriol *179*, 5094–5103. 10.1128/jb.179.16.5094-5103.1997.

S3. Savitsky, P., Bray, J., Cooper, C.D.O., Marsden, B.D., Mahajan, P., Burgess-Brown, N.A., and Gileadi, O. (2010). High-throughput production of human proteins for crystallization: The SGC experience. J Struct Biol *172*, 3–13. 10.1016/j.jsb.2010.06.008.

S4. Jankevicius, G., Ariza, A., Ahel, M., and Ahel, I. (2016). The toxin-antitoxin system DarTG catalyzes reversible ADP-ribosylation of DNA. Mol Cell *64*, 1109–1116. 10.1016/j.molcel.2016.11.014.

S5. Lalić, J., Marjanović, M.P., Palazzo, L., Perina, D., Sabljić, I., Žaja, R., Colby, T., Pleše, B., Halasz, M., Jankevicius, G., et al. (2016). Disruption of macrodomain protein SCO6735 increases antibiotic production in streptomyces coelicolor. Journal of Biological Chemistry *291*, 23175–23187. 10.1074/jbc.M116.721894.
