# Supplementary material for: Molecular basis for the reversible ADP-ribosylation of guanosine bases
Source: Mol Cell. 2023 Jul 6;83(13):2303–2315.e6. doi: 10.1016/j.molcel.2023.06.013 (PMC11543638; doi:10.1016/j.molcel.2023.06.013)
Supplement: Document S2. Article plus supplemental information [file mmc2.pdf]

Molecular Cell

Article

Molecular basis for the reversible ADP-ribosylation of guanosine bases

Graphical abstract

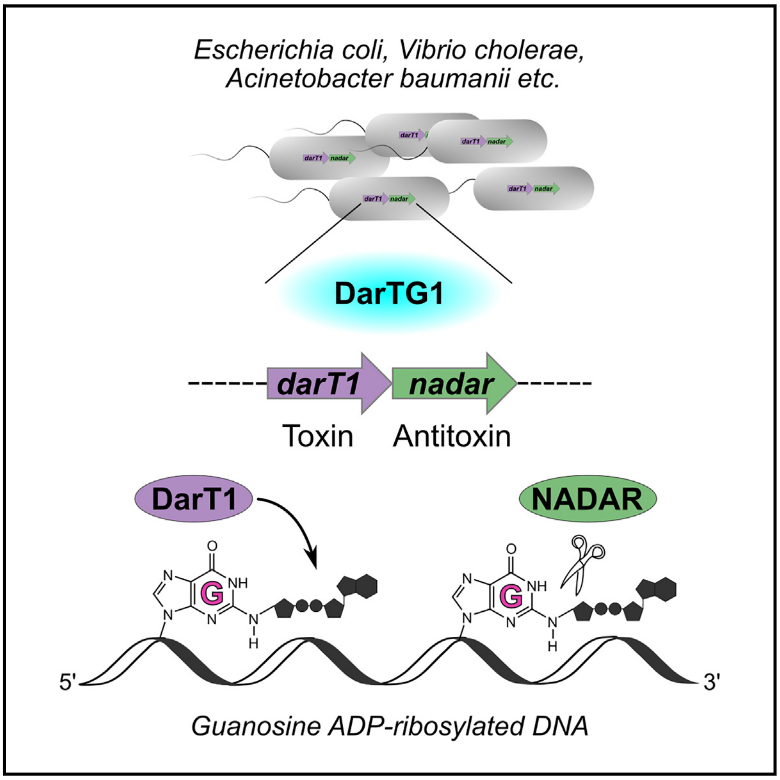

Authors

Marion Schuller, Roberto Raggiaschi, Petra Mikolcevic, ..., Christoph Tang, Andreja Mikoc, Ivan Ahel

Correspondence

ivan.ahel@path.ox.ac.uk

In brief

The toxin-antitoxin system DarTG provides control of bacterial growth and protection against bacteriophages. Schuller et al. characterize the DarTG1 subfamily as a module for reversible ADP-ribosylation of guanosine bases. Its antitoxin belongs to the NADAR enzyme family, in which hydrolytic activity on guanine-ADP-ribosylated DNA is conserved among eukaryotic and non-DarT1 toxin-associated members.

Highlights

- DarTG1 is a toxin-antitoxin system for reversible ADP-ribosylation of guanosine bases
- The toxin DarT1 links ADP-ribose to the amino group of guanines in ssDNA
- NADARs function as antitoxins by reversing DarT1-catalyzed guanine ADP-ribosylation
- Guanine de-ADP-ribosylation activity is conserved in eukaryotic NADARs

Schuller et al., 2023, Molecular Cell *83*, 2303–2315
July 6, 2023 © 2023 The Author(s). Published by Elsevier Inc.
https://doi.org/10.1016/j.molcel.2023.06.013

CellPress

Molecular Cell

CellPress
OPEN ACCESS

Article

Molecular basis for the reversible ADP-ribosylation of guanosine bases

Marion Schuller,[1] Roberto Raggiaschi,[1] Petra Mikolcevic,[2] Johannes G.M. Rack,[1] Antonio Ariza,[3] YuGeng Zhang,[1] Raphael Ledermann,[4] Christoph Tang,[1] Andreja Mikoc,[2] and Ivan Ahel[1,5,*]
[1]Sir William Dunn School of Pathology, University of Oxford, Oxford, UK
[2]Division of Molecular Biology, Ruđer Bošković Institute, Zagreb, Croatia
[3]School of Biosciences, University of Sheffield, Sheffield, UK
[4]Department of Biology, University of Oxford, Oxford, UK
[5]Lead contact
*Correspondence: ivan.ahel@path.ox.ac.uk
https://doi.org/10.1016/j.molcel.2023.06.013

SUMMARY

Modification of nucleic acids by ADP-ribosylation is catalyzed by various ADP-ribosyltransferases, including the DarT enzyme. The latter is part of the bacterial toxin-antitoxin (TA) system DarTG, which was shown to provide control of DNA replication and bacterial growth as well as protection against bacteriophages. Two subfamilies have been identified, DarTG1 and DarTG2, which are distinguished by their associated antitoxins. While DarTG2 catalyzes reversible ADP-ribosylation of thymidine bases employing a macrodomain as antitoxin, the DNA ADP-ribosylation activity of DarTG1 and the biochemical function of its antitoxin, a NADAR domain, are as yet unknown. Using structural and biochemical approaches, we show that DarT1-NADAR is a TA system for reversible ADP-ribosylation of guanosine bases. DarT1 evolved the ability to link ADP-ribose to the guanine amino group, which is specifically hydrolyzed by NADAR. We show that guanine de-ADP-ribosylation is also conserved among eukaryotic and non-DarT-associated NADAR members, indicating a wide distribution of reversible guanine modifications beyond DarTG systems.

INTRODUCTION

DNA base modifications of the canonical nucleotides occur in cellular organisms from all domains of life and viruses. A variety of modifications have been identified, including methyl groups, amino acids, polyamines, and sugars, which are associated with diverse functions and consequences, including control of gene regulation and expression, DNA repair, protection from DNA degradation, and recognition of invasive DNA.[1] The latter is a known defense mechanism in prokaryotes against bacteriophages, with 5-methylcytosine being the first modified nucleoside observed in the DNA of *Mycobacterium tuberculosis*.[2] Recently, the modification of DNA bases by ADP-ribosylation has attracted increased interest.[3] ADP-ribosylation has been traditionally studied as a reversible posttranslational modification of proteins regulating diverse fundamental processes such as transcription, DNA repair, stress and immune response, RNA biogenesis, metabolism, and microbial pathogenicity.[4–7] The modification is characterized by the transfer of ADP-ribose (ADPr) from β-nicotinamide adenine dinucleotide (β-NAD$^+$) onto acceptor sites via *N*-, *O*-, or *S*-glycosidic linkages catalyzed by ADP-ribosyltransferases (ARTs).[8] Some eukaryotic members of the ART superfamily, such as the DNA repair PARPs 1 and 2, are known for their PARylation activity, a process by which successive ADPr units are added to a growing chain.[9]

Nucleic acid ADP-ribosylation is characterized by ADPr linkage to either DNA bases or phosphorylated termini.[10] While pierisins and CARP-1, as members of the ARTC (ART-cholera toxin-like) subfamily of ARTs, target guanosine bases in dsDNA or guanosine-derived nucleosides for ADP-ribosylation,[11–15] transferases of the ART-diphtheria toxin-like (ARTD) subfamily (including the PARPs) were shown to have ADP-ribosylation activity *in vitro* against the phosphorylated ends of DNA and RNA.[16–20] Furthermore, Tre23, an antibacterial toxin delivered by the *Photorhabdus laumondii* type VI secretion system, inhibits protein translation through ADP-ribosylation of 23S ribosomal RNA.[21] This is similar to the antibacterial effector secreted by *Pseudomonas aeruginosa*, RhsP2, where cellular intoxication arises from ADP-ribosylation of 2′-hydroxyl groups of non-coding dsRNAs and tRNAs.[22] The best-characterized system that reversibly ADP-ribosylates DNA is DarTG, a toxin-antitoxin (TA) system that is widespread among prokaryotes including many human pathogens. DarT, the toxin of the system and ARTD family member, catalyzes the ADP-ribosylation of thymidine bases in ssDNA in a sequence-specific manner.[23,24] The antitoxin, DarG, counteracts DarT activity by hydrolyzing thymidine ADP-ribosylation with its catalytic N-terminal macrodomain and by forming a

This is an open access article under the CC BY license (http://creativecommons.org/licenses/by/4.0/).

complex with DarT through its predicted C-terminal DarT-binding domain.[23] As such, DarTG resembles features of both type II and IV TA systems.[25] DarT-catalyzed thymidine ADP-ribosylation induces strong bacteriostatic effects and is perceived as *bona fide* DNA damage activating the SOS response.[23,26,27] Moreover, DarT of *M. tuberculosis* was shown to preferentially ADP-ribosylate thymidines at the origin of replication, which is assumed to lead to impaired DnaB helicase activity and slow bacterial growth.[24] Yet DarTG systems are also often encoded next to phage defense elements,[23,28] in so-called defense islands,[29] suggesting the role of DarTG systems in providing bacterial defense against bacteriophages.

Indeed, using *E. coli* MG1655 as model strain, phage infection was shown to trigger the activation of DarT, which then inhibits viral DNA replication and transcription as well as RNA synthesis through ADP-ribosylation of phage DNA.[28] Consequently, this process blocks the production of mature phages; yet because some transcription is thought to occur, particularly early on, phages are able to degrade the bacterial host chromosome. Although the host bacterium does not recover, it protects its community by preventing the release of infectious phages.[28] A comprehensive bioinformatic search for TA systems involved in phage defense also identified two different subfamilies of DarTG, termed DarTG1 and DarTG2, which both confer phage protection but with different antitoxins associated with the ARTD-type toxins. Analysis of neighboring genes by de Souza and Aravind[30] uncovered that DarT (classified to the BC4486-like ART family) is encoded adjacent to genes not only for macrodomain proteins (DarTG2 class) but also for NADAR domain-containing enzymes (DarTG1 class), belonging to a family of YbiA-related enzymes previously termed DUF1768. The NADAR superfamily was named based on its potential association with "NAD$^+$ and ADP-ribose" pathways including NAD$^+$ metabolism and RNA processes and was grouped into two main subfamilies: the "YbiA family" including *E. coli* YbiA and the "BC4488 family" including NADARs encoded next to DarT.[30] NADARs are widespread in distinct bacterial, eukaryotic, and viral lineages yet absent from vertebrates.[30] Bacterial and plant NADAR enzymes are generally known for their *N*-glycosidic activity removing reactive intermediates of the riboflavin biosynthesis pathway,[31] and in *E. coli* K-12, *ybiA* was also identified among genes relevant for swarming motility.[32] However, the enzymatic activities and physiological roles of the enzymes in the BC4488 NADAR subfamily found to be associated with DarT1 are poorly understood.

In this study, we biochemically and structurally characterized TA system-associated NADARs (DarG1) with their accompanying DarT1 toxin, and as such DarTG1. We discovered that in contrast to macrodomain-associated DarTs (DarT2), which catalyze thymidine base ADP-ribosylation, NADAR-associated DarT1 ADP-ribosylates guanosine bases, while the associated NADAR domains are hydrolases for this modification, rendering DarTG1 a TA system for reversible DNA ADP-ribosylation of guanosines. Crystal structures of DarT1 in pre- and post-reaction states revealed the DarT1-catalyzed ADPr-guanine linkage and provide mechanistic insights into this reaction. Furthermore, we support our studies with the first crystal structures of NADARs in their ligand-free and ADPr-bound states to structurally characterize the DarT1-associated BC4488 subfamily NADARs in comparison with orphan YbiA subfamily NADARs using that from the plant-pathogenic oomycete *Phytophthora nicotianae* var. *parasitica* as an example. We found that the non-DarT1-associated NADAR domain of *P. nicotianae* var. *parasitica* is also capable of hydrolyzing guanine-linked ADP-ribosylation, suggesting the latter presents a conserved biochemical activity of NADAR superfamily members.

RESULTS

NADARs as antitoxins of DarTG TA systems

Genomic context analysis and bioinformatic TA system searches[28,30] identified two DarTG systems that have the ART toxin gene *darT* in an operon with either *darG* or *nadar*. The DarG-associated DarT system was the first DarTG system characterized[23,24,26] and classified as "DarTG2,"[28] while the NADAR-associated DarT system was termed "DarTG1," with DarG1 being a NADAR (Figure 1A, left). DarT2 was shown to catalyze the transfer of ADPr from NAD$^+$ onto thymidines in ssDNA (Figure 1A, right), thereby linking the anomeric carbon of the distal ribose to the in-ring nitrogen N3 of the thymine.[24] The DarT2-ADP-ribosylated DNA product is recognized and hydrolyzed by the antitoxin DarG via its macrodomain with residue K80 critical for catalysis[23] (Figure 1B, starred residue). The C-terminal region of DarG linked to the macrodomain was identified as the DarT2-binding domain enabling antitoxin neutralization of DarT2 activity through complex formation. Initial comparison of the two DarTG systems suggested a sequence alignment of the DarT1-associated NADAR domain with the DarT2-binding domain of DarG, with both domains described as "YbiA-like."[28] However, close inspection of secondary structure elements and conserved folds in both domains revealed no similar arrangement of predicted α helices and β sheets, with the only confirmed presence of a YbiA-like fold in the DarT1-associated antitoxin (Figure 1B). This identifies that the DarT1-associated antitoxin is a single domain that belongs to the NADAR family, as opposed to the DarG2 protein, which has two domains (Figure 1B). Next, we investigated whether DarT1-NADAR is also a functional TA system similar to DarT2-DarG. Indeed, lethality of *E. coli* cells expressing DarT1 from *E. coli* C7 or the thermophile *Geobacter lovleyi* could be rescued by the co-expression of the cognate NADAR (Figure 1C). Of note, *E. coli* C7 was used in previous studies as a representative species for characterizing the role of DarTG1 systems in phage defense[28] and was therefore also included in our study as the preferred model species. Moreover, and consistent with previous observations made with DarTG2, NADARs from non-cognate species were also protective against DarT1 toxicity (Figure 1D). Thus, we confirmed that DarTG1 is a TA system similar to DarTG2, albeit with a NADAR domain protein as the antitoxin for DarT1.

Phylogenetic analysis further showed that DarT1-associated NADARs form a distinct clade of the NADAR superfamily and are divergent from other branches, including the non-DarT1-associated bacterial NADARs and YbiA-like class (Figure 1E). Members of the latter that are associated with bacterial motility seem to be closely related to the flavin biosynthetic NADARs in plants and fungi. Furthermore, obvious NADAR homologs can be generally

CellPress
OPEN ACCESS

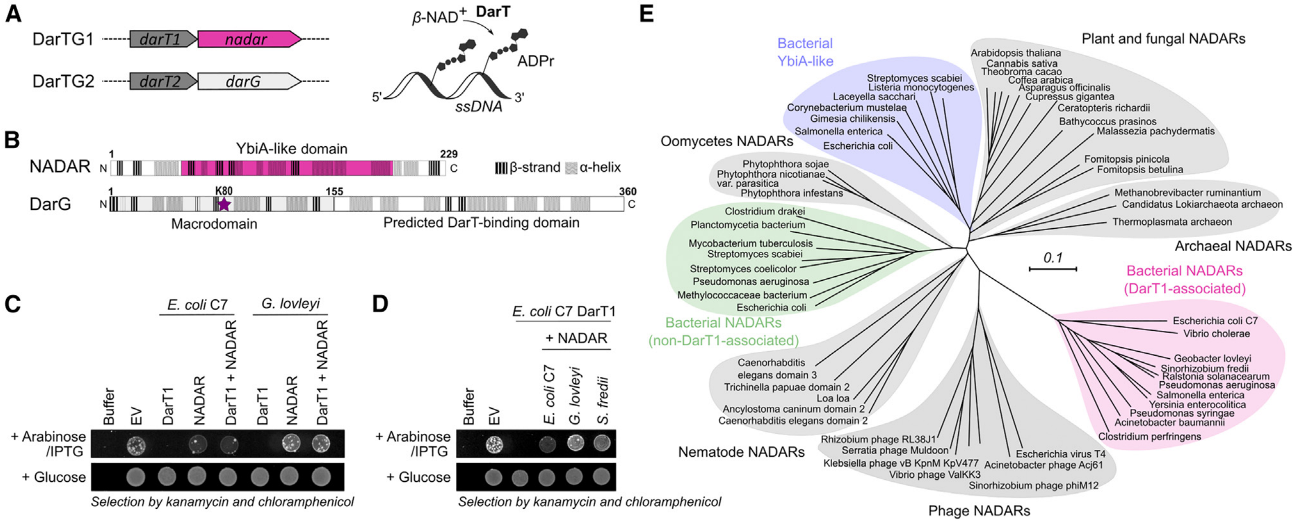

Figure 1. NADARs as antitoxins of DarTG TA systems

(A) Schematics comparing the two identified DarTG operonal systems (left) and the molecular reaction of its encoded toxin, DarT (right). DarT catalyzes the ADP-ribosylation of ssDNA by transferring ADPr from β-NAD$^+$ onto DNA bases.

(B) Comparison of predicted secondary structure elements of the operonal DarT-associated domains NADAR and DarG using PSIPRED. The characterized catalytic residue of DarG, K80, is highlighted with a star.

(C) DarT-NADAR is a toxin-antitoxin system. Toxicity assay monitoring the growth of *E. coli* BL21-DE3 under repression (glucose) and induction (arabinose/IPTG) of the expression of DarT1 or/and NADAR is shown. Representative of three biologically independent experiments. EV, empty vector control.

(D) NADARs of non-cognate species rescue from DarT1 toxicity. Representative of three biologically independent experiments as performed in (C).

(E) Unrooted phylogenetic tree of the NADAR superfamily with representative archaeal, bacterial, fungal, eukaryotic, and viral members of the different branches shown. The corresponding alignment is shown in Figure S4 and NCBI accession numbers are listed in Table S2.

identified in several eukaryotic subgroups but are absent in chordata. Finally, we also noted that the majority of NADARs (including DarT1-associated NADARs) appear to be single and free-standing domains in comparison to NADAR proteins in plants,[31] which were often found to be fused to other functional domains, perhaps for synergistic and coordinated activities.

NADARs reverse guanine ADP-ribosylation catalyzed by DarT1

Phage infection was shown to increase ADP-ribosylation of phage DNA in *E. coli* harboring the wild-type DarTG1 or DarTG2 system over cells expressing the respective systems with catalytically inactive DarTs.[28] We confirmed the DNA ADP-ribosylation activity of DarT1 by overexpression of *E. coli* C7 and *G. lovleyi* DarT1 in *E. coli* BL21 cells followed by analysis of the extracted and purified gDNA.[24] As expected, the presence of ADP-ribosylated gDNA correlated with the bacteriostatic effect of DarT1-induced toxicity (Figure 2A). When testing *E. coli* C7 DarT1 activity on the established 27-mer ssDNA oligonucleotide that contained the preferred four-base motif of *Thermus aquaticus* DarT2 (TNTC, with the ADP-ribosylated thymidine being in the third position), we first noticed a different ADP-ribosylation modification pattern compared to that produced by *T. aquaticus* DarT2. Multiple shifts appeared indicating the addition of several ADP-ribosylation modifications by DarT1 (Figure 2B lane 2; Figure S1). Furthermore, while DarG was able to remove the DarT2-catalyzed modification, DarT1-associated NADARs appeared to be inactive on the thymine-linked ADP-ribosylation modification (Figure 2B lane 1). Instead, the same NADAR enzymes showed very efficient hydrolysis of *E. coli* C7 DarT1-catalyzed ADP-ribosylation modifications on which DarG was inactive (Figure 2B lane 2). We then compared DarG and NADARs on an oligonucleotide ("polyT-G") modified by *Streptomyces coelicolor* ScARP (Figure 2B lane 3), which catalyzes the ADP-ribosylation of guanosine bases in DNA.[11,33] Interestingly, ScARP-mediated guanine ADP-ribosylation was reversed by NADARs from three different species, while DarG did not catalyze this hydrolysis (Figure 2B lane 3). Thus, we conclude that (1) DarT1 is an ART catalyzing guanine ADP-ribosylation, and (2) NADARs are hydrolases that specifically reverse guanine ADP-ribosylation. The 27-mer oligonucleotide would provide *E. coli* C7 DarT1 with several guanine modification sites, compared to DarT2 with a single thymine ADP-ribosylation site, resulting in the observed difference in ADP-ribosylation patterns of the toxins. To see if the hydrolysis of guanine ADP-ribosylation is a conserved activity of NADAR family members, we tested the non-DarT1-associated NADAR protein of *P. nicotianae* var. *parasitica* in the same experimental setup. We observed the reversal of DarT1- and ScARP-catalyzed guanine ADP-ribosylation (Figure 2C lanes 2 and 3) and its inactivity on DarT2-catalyzed thymine ADP-ribosylation modification (Figure 2C lane 1). This supports our hypothesis that guanine-ADPr hydrolysis might be a conserved function among NADARs.

Regarding the substrate preference of DarT1, we confirmed that *E. coli* C7 DarT1 is able to ADP-ribosylate all guanosine sites in the DarT-ADPr-27-mer, yet with no double modification at the "AGGA" site (possibly by the first ADP-ribose modification sterically restricting a consecutive modification on the adjacent

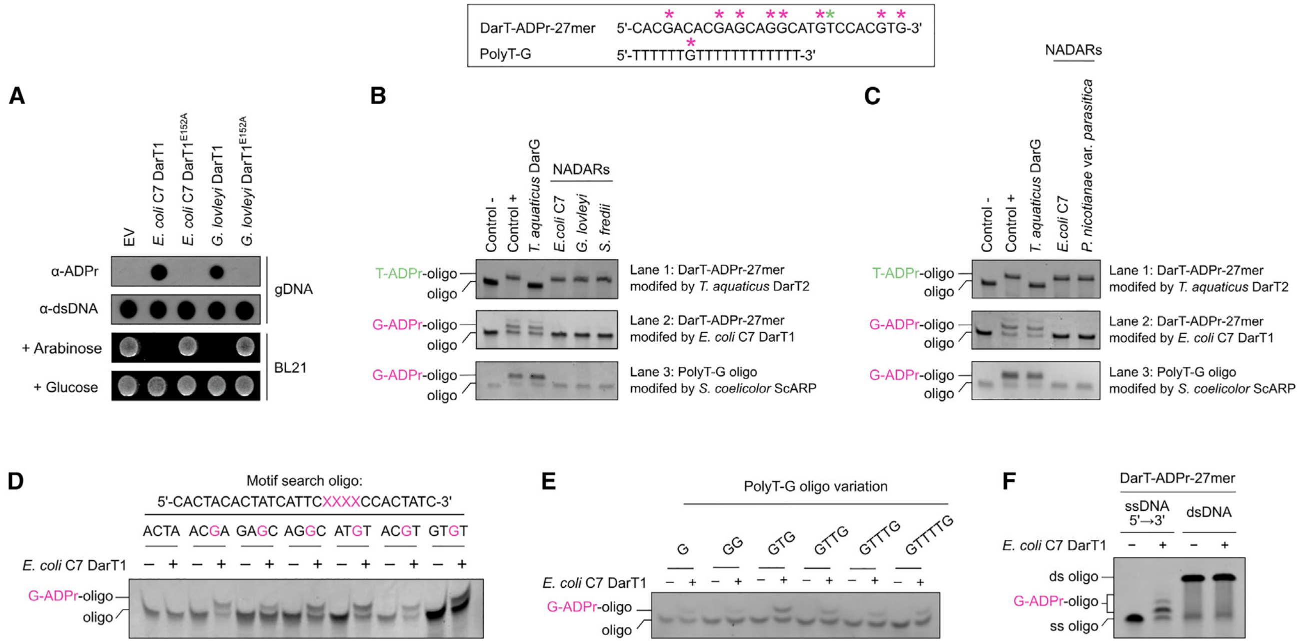

Figure 2. NADARs reverse guanine ADP-ribosylation catalyzed by DarT1

(A) Cellular toxicity of DarT1 is induced by its DNA ADP-ribosylation activity. Dot blot showing DNA ADP-ribosylation activity by *E. coli* C7 and *G. lovleyi* wild-type DarT1 and mutants on gDNA is presented above toxicity assay monitoring the growth of *E. coli* BL21 under repression (glucose) and induction (arabinose) of the expression of DarT1. EV, empty vector control.

(B) NADARs transcriptionally linked to DarT1s hydrolyze ADP-ribosylation on guanine (“G-ADPr”) and are inactive on thymine ADP-ribosylation (“T-ADPr”) modifications, in contrast to DarG. Guanine ADP-ribosylation is catalyzed by DarT1 and ScARP, while thymine ADP-ribosylation is catalyzed by DarT2. *In vitro* ADP-ribosylation assays were performed using the substrates shown in the box, with the known thymidine and guanosine nucleotides targeted for ADP-ribosylation by the characterized toxins, i.e., *T. aquaticus* DarT2 and *S. coelicolor* ScARP, highlighted with an asterisk. Representative of three independent experiments.

(C) Non-TA-system-associated NADARs of higher species including *P. nicotianae* var. *parasitica* also catalyze guanine de-ADP-ribosylation and are hydrolytically inactive on ADP-ribosylated thymidines. Representative of three independent experiments as performed in (B).

(D) *In vitro* ADP-ribosylation assay showing ability of *E. coli* C7 DarT1 to ADP-ribosylate all guanosine sites in the DarT-ADPr-27-mer, yet with no double modification of the “AGGC” site. The assay uses an oligo with four bases around the to-be-tested guanosine of the DarT-ADPr-27-mer flanked by random guanosine-free sequences, considering a four-base motif with the targeted nucleotide in third position as for DarT2 activity.

(E) *E. coli* C7 DarT1 ADP-ribosylation activity on PolyT-G oligomer variations. The presence of two guanosine bases with a spacing of one base favors the modification reaction.

(F) *In vitro* ADP-ribosylation activity of *E. coli* C7 DarT1 on the substrate “DarT-ADPr-27mer” as ssDNA and dsDNA is shown. (D, E, F) Representatives of three independent experiments.

guanosine) (Figure 2D). This suggests that DarT1 may have a certain motif specificity, which is, however, more relaxed compared to DarT2. Furthermore, testing ADP-ribosylation activity of DarT1 on variations of the PolyT-G oligomer, we observed that having two guanosine bases with a spacing of one base favors the modification reaction (Figure 2E). Considering that DarT2 recognizes a four-base motif with the thymidine in third position being modified and the base in first position being very specifically recognized (typically a thymidine as well), the first guanosine base of “GTGT” may also be recognized by DarT1, resulting in stabilization of the DNA substrate for more efficient ADP-ribosylation of the base in third position (Figure 2E). Finally, similarly to DarT2, we were not able to detect DarT1 activity on dsDNA (Figure 2F).

DarT1 is a guanine-specific ART

To confirm and characterize DarT1-catalyzed guanine ADP-ribosylation, we determined the structures of full-length *E. coli* C7 DarT1^{E152A} in its pre- and post-reaction states. Co-crystallization of *E. coli* C7 DarT1^{E152A} with NAD^{+} as well as with carba-NAD^{+}, a non-hydrolyzable NAD^{+} analog, and an ssDNA 5-mer allowed insights into the substrate-bound state of DarT1, while co-crystallization with NAD^{+} and the ssDNA 5-mer gave a structure at 1.63 Å of the product-bound state (Figures 3A, 3B, and S2). Similar to DarT2 and ARTs in general, DarT1 also binds the NAD^{+} substrate with the NAD^{+}-binding loop in a binding mode of constrained conformation over the central fold-stabilizing 6-stranded β sheet core (Figure 3A). The adenine moiety is stabilized by hydrogen bonding to G32 and M33 backbone amides and the side chain of S26 of DarT1. The adenine-proximal ribose bonds with its 2′-hydroxyl group to H17 and with its 3′-hydroxyl group over water contacts to T19 and the β-phosphate. The nicotinamide (NAM) moiety is fixed in position by an offset, stacked π-π interaction with F18 and an intramolecular hydrogen bond of its primary amide to the β-phosphate. The latter also bonds with the N48 side chain, in comparison to the

CellPress
OPEN ACCESS

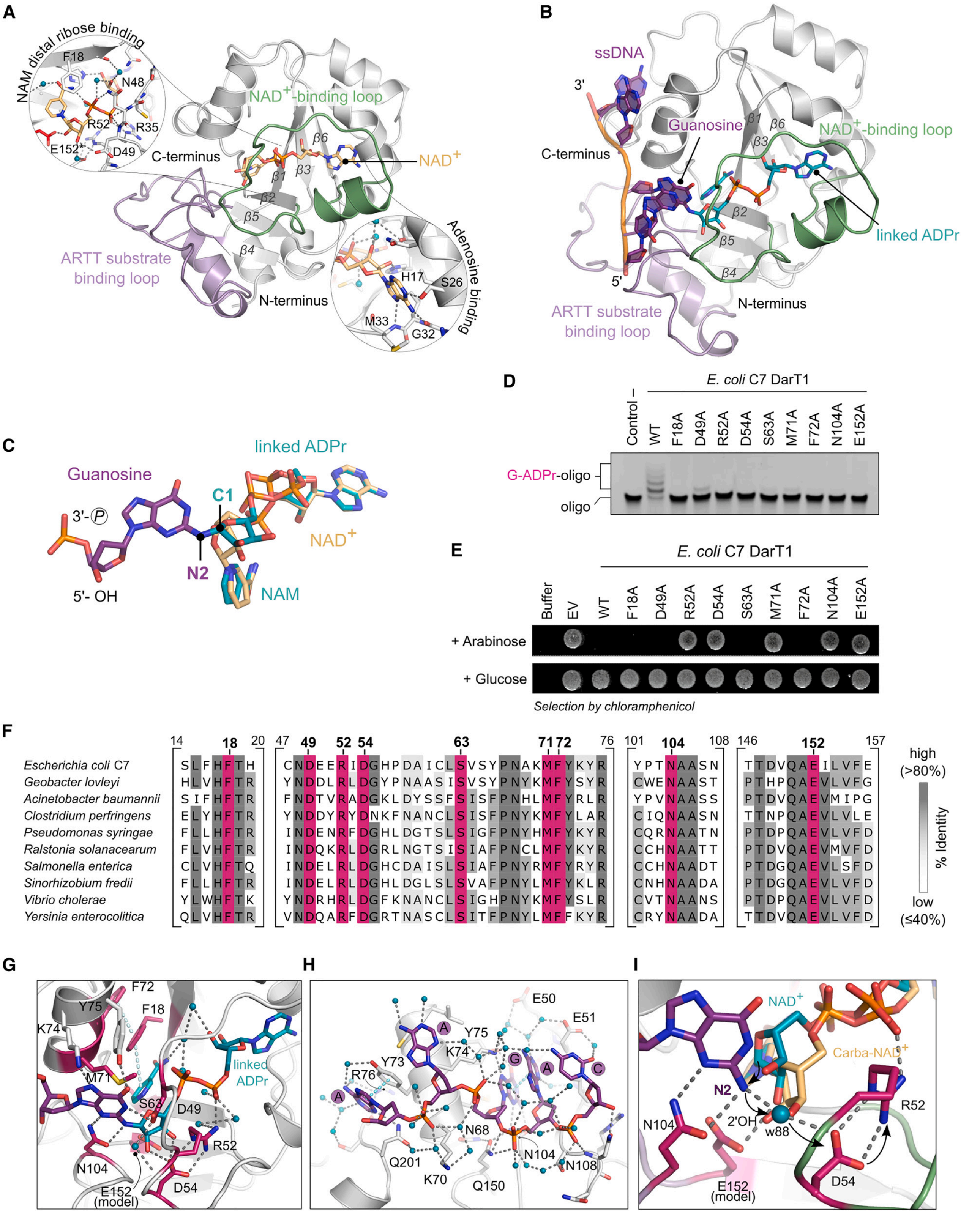

(legend on next page)

α-phosphate, which is stabilized through the N48 and D49 backbone amides and interactions with R35 and R52 side chains (Figure 3A). Co-crystallization of DarT1^{E152A} with NAD$^+$ and DNA allowed capture of the post-reaction state, that is after NAD$^+$ cleavage and reaction with the DNA substrate *in crystallo* (Figure 3B). This structure revealed that DarT1 indeed catalyzes the linkage of ADPr to the guanosine base of the DNA strand, leaving the NAM ligand in the substrate binding site. The DNA is held onto the ARTT substrate binding loop, directing the guanine into the active site of DarT1 for ADP-ribosylation. Furthermore, the structure unambiguously revealed the DarT1-established connection of the distal ribose C1 with the N2 of the amino group of the guanosine base (Figures 3C and S2C). With accompanying mutagenesis studies on *E. coli* C7 DarT1, we confirmed active-site residues relevant for catalyzing ADP-ribosylation of guanosine (Figures 3D and 3E). All tested alanine substitutions of active-site residues notably decreased the *in vitro* ADP-ribosylation activity of purified *E. coli* C7 DarT1 mutants to such an extent that guanosine ADP-ribosylation could not be observed anymore with the chosen assay conditions (Figure 3D). The more sensitive toxicity assays revealed the active-site residues that are essential for the guanine-ADPr catalysis mechanism and that have also been identified for DarT2 as critical for catalyzing DNA base ADP-ribosylation (Figure 3E). Alanine substitutions of E152 (the ART-characteristic glutamate), N104 (the target base-coordinating residue corresponding to H119 in DarT2), M71 (as potentially oxocarbenium intermediate-stabilizing residue), and R52, relevant for proton-abstraction in DarT2, all resulted in an inactive DarT1 with associated loss of toxicity (Figures 3E and 3G). These residues, which are essential for catalysis, are highly conserved in DarT1 from various bacterial species (Figure 3F), as are F18 and F72, which, though involved in NAM coordination through π-π interaction, are generally dispensable for catalysis but critical for the ADP-ribosylation efficiency (Figures 3D, 3E, and 3G). Of note, DarT1 and DarT2 display different arrangements of the NAM-coordinating residues (Figure S5). In DarT2, this function is served by Y71, which is part of the ARTD-class defining [H-Y-E] motif, but DarT1 shows a conserved but mechanistically dispensable serine (S63) in this position (Figures 3E and 3F) and a shift of the aromatic residues into the β1 sheet and the ART-conserved α helix between β2 and β3 (Figure S5). In addition, alanine substitution of the conserved D54 residue also resulted in a loss of the bacteriostatic effect of DarT1, indicating an essential mechanistic role for D54 in DarT1-catalyzed guanine ADP-ribosylation (Figures 3E and 3F). The interplay of key residues in the active site for catalyzing the reaction is enabled by their proximity, allowing coordination of the guanosine, the NAM moiety, and the distal ribose, which promote NAD$^+$ polarization, NAM-ribose bond cleavage, and generation of an oxocarbenium ion intermediate (Figure 3G). The guanine is held in position for reaction by N104 through hydrogen bonding to N2 and N3 as well as by Y75 and K74, which recognize the guanine carbonyl oxygen C6 and N7, respectively (Figure 3G). The tight coordination of the guanine guarantees base specificity of the reaction and contrasts with the overall less specific DNA binding mode. While the first and fourth base show π-π stackings to Y73 or the active-site guanine, respectively, base-protein interactions are mainly coordinated over water-mediated contacts. The phosphate-ribose backbone is held in position by few direct hydrogen bonds, including to Q150 of the active-site guanosine phosphate, and through an extensive water network involving the bases and DarT1 (Figure 3H). The differences in structural features between DarT1 and DarT2 are presented in more detail in Figure S5. Of particular note is that comparison of DarT1 in the carba-NAD$^+$:DNA-bound state (Figures S2A and S2B) with the ADPr-DNA-bound state revealed that the side-chain flexibility of R51 in DarT2 could not be observed in DarT1. The corresponding arginine residue in DarT1 (R52) hydrogen bonds in all captured states to the β-phosphate and is furthermore ideally hydrogen-bonded by D54, indicating their interplay for catalysis (Figure 3I). Thus, it can be hypothesized that guanine ADP-ribosylation is

Figure 3. DarT1 is a guanine-specific ADP-ribosyltransferase

(A) NAD$^+$ substrate recognition by DarT1. Cartoon stick model of the co-crystal structure of full-length *E. coli* C7 DarT^{E152A} with NAD$^+$ (brown sticks). The substrate-binding ARTT loop is highlighted in purple, and the NAD$^+$-binding loop is in green. The transferase-characteristic six β sheet core is labeled. The circular insets show the interaction network between NAD$^+$, protein residues (gray sticks), and waters (blue spheres). The asterisk indicates that E152 (red sticks) is modeled into the structure.

(B) Co-crystal structure of full-length *E. coli* C7 DarT1^{E152A} in product (ADPr-DNA and NAM)-bound state after NAD$^+$ cleavage and reaction *in crystallo*. The substrate-binding ARTT loop is highlighted in purple, and the NAD$^+$-binding loop is in green.

(C) Enlarged view of the ADPr-linked guanosine in overlay with NAD$^+$ as resolved in the co-crystal structures shown in (A). DarT1 establishes the connection between the distal-ribose C1 and the N2 of the guanosine base.

(D) ADP-ribosylation activity of *E. coli* C7 DarT1 active-site mutants in comparison to wild type (WT) visualized after a reaction time of 1 h. Representative of three independent experiments.

(E) Toxicity assay monitoring the growth of *E. coli* BL21 under repression (glucose) and induction (arabinose) of the expression of *E. coli* C7 DarT1 WT and catalytic site mutants. EV, empty vector control. Representative of two biologically independent experiments.

(F) Multiple sequence alignment showing conservation of active-site residues among DarT1. Numbers on top of the residues refer to *E. coli* C7 DarT1. The overall sequence identity of selected species in relation to *E. coli* C7 DarT1 is between 35% and 40%.

(G) Close-up view on the active site of *E. coli* C7 DarT1 in complex with ADPr-DNA, with residues tested for their relevance for DarT1 DNA ADP-ribosylation activity in (D) and (E) highlighted in pink. Waters are shown as blue spheres. Interactions between side chains, waters, and ligands are indicated with gray dashed lines.

(H) View of the DNA binding site of *E. coli* C7 DarT1 in complex with ADPr-DNA. Waters are shown as blue spheres and interactions between side chains, waters, and the ssDNA strand of the sequence AAGAC are indicated with gray dashed lines.

(I) Overlay of the crystal structures of *E. coli* C7 DarT1^{E152A} in complex with NAD$^+$ and carba-NAD$^+$:DNA to represent the pre-reaction, i.e., NAD$^+$:DNA-bound state. The distal-ribose C1 linkage to the base and the potential proton transfer from the guanine N2 onto D54 and R52 over the water molecule w88 involved in the mechanism is indicated with black arrows.

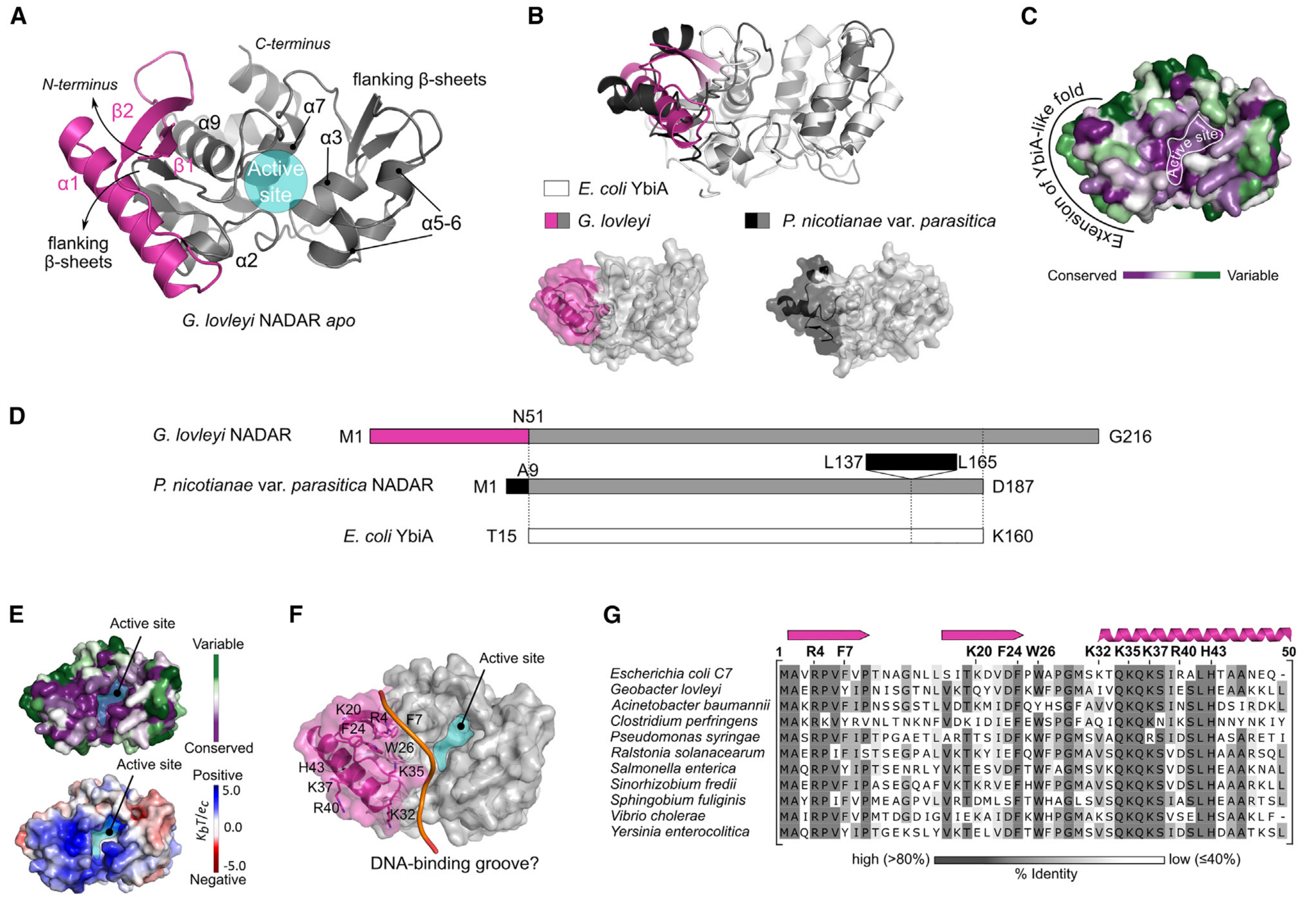

Figure 4. The NADAR domain displays an extended *E. coli* YbiA-like fold

(A) Crystal structure of *G. lovleyi* NADAR in ligand-free state. The active site is formed by an α-helical core (gray) that is N-terminally extended with two β sheets and one pronounced α helix (pink).

(B) Comparison of the NADAR crystal structures of *G. lovleyi* NADAR and *P. nicotianae* var. *parasitica* NADAR by an overlay with an *E. coli* YbiA NMR solution structure (PDB: 2B3W; white cartoon). The YbiA-like fold extension of the two NADAR species is highlighted in pink and black, with an overlayed cartoon representation at the top and surface representations of each below.

(C) The active site of NADARs is highly conserved among the NADAR superfamily. Residue conservation analysis was carried out using the ConSurf server and mapped onto an *E. coli* C7 NADAR AlphaFold2 model, with coloring representing continuous conservation scores partitioned into nine bins for visualization.

(D) Sequence schematic comparing the structural makeup of *G. lovleyi* NADAR and *P. nicotianae* var. *parasitica* NADAR compared to *E. coli* YbiA. The YbiA-like fold extensions are highlighted in pink and black. The first secondary structure element of *E. coli* YbiA begins at T15.

(E) Amino acid residue conservation using the Consurf server (top) and surface electrostatic potential using APBS (bottom) mapped onto the surface of an *E. coli* C7 NADAR AlphaFold2 model show the high conservation of a positive surface of the N-terminal YbiA-like fold extension of DarT1-associated NADARs.

(F) The N-terminal YbiA-like fold extension (pink) bears several basic and hydrophobic residues potentially involved in ssDNA binding. An *E. coli* C7 NADAR AlphaFold2 model is shown with the potential DNA-binding groove indicated for clarity with an orange line.

(G) Multiple sequence alignment highlighting the conservation and the structural position of residues in the N-terminal YbiA-like fold extension of DarT1-associated NADARs. The overall sequence identity of selected species in relation to *E. coli* C7 NADAR is between 48% and 57%.

characterized by a nucleophilic attack of the distal ribose C1 of the reactive oxocarbenium ion (an intermediate in the S_N1 mechanism of ARTs[34,35]) on the N2 nitrogen of the coordinated guanosine substrate with concomitant proton transfer, over a water molecule (w88), to D54 and R52 (Figure 3I).

The NADAR domain displays an extended YbiA-like fold

To understand the biochemical activity of the NADAR domain, we obtained crystal structures of the DarT1-associated NADAR antitoxin from *G. lovleyi* in ligand-free state as well as an ADPr substrate co-structure of the NADAR domain of eukaryotic *P. nicotianae* var. *parasitica* that is not linked with DarT. The *G. lovleyi* NADAR domain displays a globular fold and is characterized by an α-helical core that forms the active site flanked by two small β sheet regions, comprising two and three strands, respectively (Figure 4A). The core domain generally overlays with *E. coli* YbiA, yet displays some structural differences such as a different positioning of the N-terminal-distant flanking β sheet region and a split α helix (α5 and α6), both of which are absent from *P. nicotianae* var. *parasitica* NADAR (Figures 4A and 4B). However, most noticeable is an N-terminal extension of the *E. coli* YbiA domain with two β sheets and one pronounced

α helix (β1-β2-α1) in *G. lovleyi* NADAR (Figure 4B, highlighted in pink) and with a short N-terminal β sheet and three short α helices in *P. nicotianae* var. *parasitica* NADAR (Figure 4B, highlighted in black) adjacent to the active site. Conservation analysis reveals that while the active site of the NADAR domain is highly conserved among the superfamily, the *E. coli* YbiA domain extension is more variable (Figure 4C). Of note, although the N-terminal domain extension spatially takes the same position in *G. lovleyi* and *P. nicotianae* var. *parasitica* NADAR (Figure 4B), the extension is encoded in *G. lovleyi* NADAR only by an N-terminal sequence preceding the YbiA-like fold, whereas in *P. nicotianae* var. *parasitica*, it is encoded by both a short N-terminal sequence and an insertion within the YbiA-like fold (Figure 4D). Amino acid residue conservation and surface electrostatic potential mapped onto the surface of an *E. coli* C7 NADAR AlphaFold2 model further reveal the high conservation of a positive surface of the extension of the YbiA-like core among DarT1-associated NADARs (Figure 4E). The positive electrostatic potential is generated by several basic residues, which could be involved, together with conserved hydrophobic residues, in ssDNA recognition and binding (Figures 4F and 4G). This hypothesis is further supported by the observation of a potential DNA binding groove, formed by attachment of the N-terminal extension to the core domain, which may allow for interactions with ADP-ribosylated DNA substrates (Figure 4F).

Molecular basis for guanine ADPr hydrolysis

Structural studies of *G. lovleyi* and *P. nicotianae* var. *parasitica* NADAR in complex with ADPr along with mutagenesis of *E. coli* C7 NADAR elucidated the mechanisms behind ADPr substrate recognition and hydrolysis by NADARs. The ADPr ligand in the *P. nicotianae* var. *parasitica* NADAR crystallographic map displayed a bent binding mode in the open active site, with the protection of its pyrophosphates by the basic side chains of K75 and R79 (Figure 5A). The adenine head, which directs the N6 amino group toward the active site, is stabilized by π-π interaction with Y84 and hydrogen bonds to K50 and the backbone amides of G78 and V81, the latter water mediated. The adenine-proximal ribose is recognized only by the D86 side chain interacting with the outwards-facing 3′-hydroxyl group, in contrast to the pyrophosphates, which are stabilized by multiple hydrogen bond interactions including K50, R79, W89, R93, W133, and (water-mediated) D130. The distal ribose is coordinated over all three hydroxyl groups, thereby forming either direct (2″) or water-mediated (3″ and 4″) hydrogen bonds to E43 and E125 (Figure 5A). Bioinformatic studies of the NADAR superfamily[30] classified the YbiA subfamily members based on the presence of this highly conserved glutamate, E125, which is replaced with aspartate in the BC4488 NADAR subfamily and with histidine in phage NADARs. The residue conservation together with proximity to the catalytic center thus suggests their mechanistic relevance in NADAR hydrolases (Figure 5B). Indeed, alanine and asparagine substitution of D171 in *E. coli* C7 NADAR (in the structurally equivalent position of E125) resulted in loss of efficient guanine de-ADP-ribosylation activity (Figures 5B, 5C, and S3). This is in contrast to mutations of E88 (corresponding to E43 in *P. nicotianae* var. *parasitica* NADAR), which coordinates from the opposite side of the distal ribose binding cleft. While E88A had only a minor effect on substrate catalysis *in vitro* compared to the wild-type NADAR domain, E88Q mutation resulted in inactivity of the enzyme, potentially by additionally sterically inferring with recognizing the distal ribose of the ADP-ribose ligand (Figures 5B, 5C, and S3). Mutagenesis also revealed that lysine K95 (corresponding to K50 in *P. nicotianae* var. *parasitica* NADAR) plays an essential role in efficient ADPr-guanine hydrolysis, suggesting that phosphate coordination is critical for NADAR function (Figures 5B, 5C, and S3). Testing the *E. coli* C7 NADAR^{K95R} mutant also revealed that the basic function of lysine can be substituted by arginine to allow ADP-ribose recognition and perform guanine-ADPr hydrolysis *in vitro* (Figure S3). Yet the arginine mutation may either lower hydrolytic efficiency or impact NADAR antitoxin functionality in addition to its hydrolytic activity to such an extent that the mutant is not able to rescue from DarT1-toxicity anymore (Figure 5D). Apart from K95, the catalytic relevance of E88 and D171 was also confirmed by toxicity assays showing that substitutions of these residues with alanine and closely related amino acids resulted in their inability to rescue *E. coli* from *E. coli* C7 DarT1-induced toxicity (Figure 5D). Furthermore, *in vivo* toxicity assays revealed that K115 of *E. coli* C7 NADAR is also essential to counteract DarT toxicity (Figure 5D). Interestingly, the corresponding residue in *P. nicotianae* var. *parasitica* NADAR (K75) does not directly interact with the ADPr ligand despite being located close to the NADAR active site (Figure 5B). K115 is dispensable for catalysis (Figure 5C), and instability of the mutant protein is not likely based on the purification profile, indicating that this lysine residue is potentially involved in DNA substrate recognition and/or DarT1 interaction relevant in the physiological context. K115 of *E. coli* C7 NADAR is conserved among the DarT1-associated NADARs, as are the residues catalyzing guanine de-ADP-ribosylation, namely E88, K95, and D171 (Figure 5E). However, of these, only E88 and K95 are largely conserved by sequence over the NADAR superfamily (Figure S4). The multiple sequence alignment also suggests the conservation of a catalytic glutamate within the NADAR superfamily, corresponding to E173 in *E. coli* C7 NADAR; however, it is not functionally relevant for guanine-ADPr hydrolysis or DarT1 toxicity rescue (Figures 5C and 5D). Instead, this catalytic function is provided in DarT1-associated NADARs by an aspartate (D171 in *E. coli* C7) as discussed above. Thus, this arrangement of catalytic residues and the presence of a lysine residue highly conserved among DarT1-associated NADARs may be specific features of NADARs functioning as antitoxins reversing DarT1-catalyzed guanine ADP-ribosylation.

DISCUSSION

In this study, we characterize the DarT-NADAR system as a TA system catalyzing the specific and reversible ADP-ribosylation of guanosine bases, thereby providing new insights into ARTs and nucleic acid ADP-ribosylation. The DarT-NADAR (DarTG1) system shows similarities with the previously characterized DarTG TA system; however, its ART DarT1 toxin shows guanine ADP-ribosylation specificity, in contrast with the thymine substrate specificity of DarT2. The macrodomain-containing DarG antitoxin in DarTG2 is correspondingly replaced in the

CellPress
OPEN ACCESS

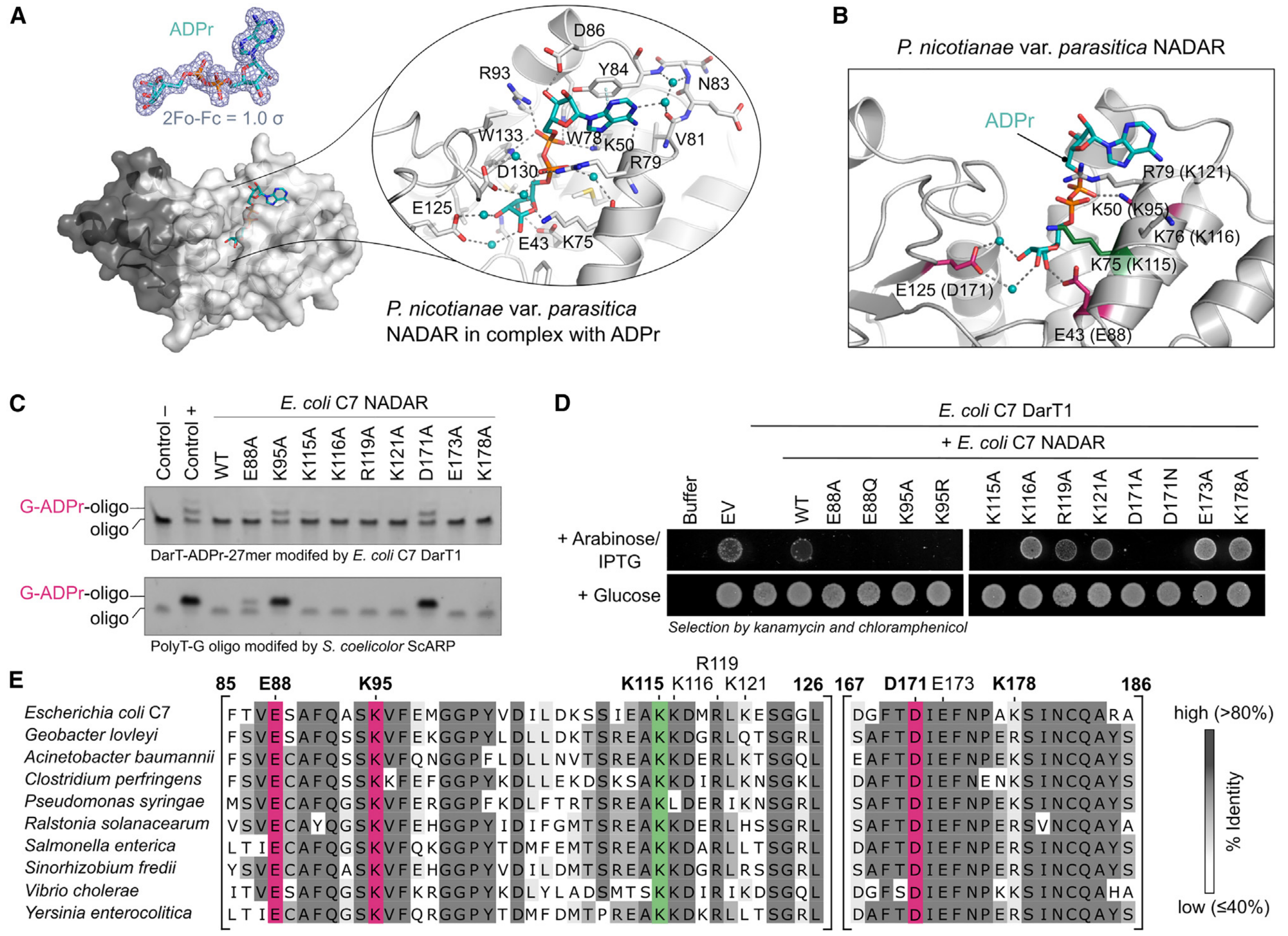

Figure 5. Molecular basis for guanine ADPr hydrolysis

(A) The co-crystal structure of *P. nicotianae* var. *parasitica* with ADPr gives insight into NADAR substrate recognition. Left top: The $2F_o$-F_c electron density map contoured at 1.0 σ around the ADPr ligand as in the structure is shown in gray. Left bottom: Cartoon surface representation of the structure is shown with ADPr as stick model. Right: Cartoon stick model of the active site showing the interaction network between ADPr (cyan stick model), protein residues (gray sticks), and waters (blue spheres).

(B) Closed-up view on the active site of *P. nicotianae* var. *parasitica* NADAR co-crystal structure with ADPr. Mechanistically relevant residues are in pink, and the *E. coli* C7 NADAR-corresponding residue rescuing from DarT1 toxicity is in green. Corresponding *E. coli* C7 NADAR residues are provided in parentheses.

(C) *In vitro* guanine-ADPr hydrolytic activity of *E. coli* C7 NADAR active-site mutants compared to wild type on two different oligo substrates (sequences shown in Figure 2). Representative of three independent experiments.

(D) Toxicity assay monitoring the growth of *E. coli* BL21-DE3 under repression (glucose) and induction (arabinose/IPTG) of the expression of *E. coli* C7 DarT1 and NADAR wild-type or catalytic site mutants. EV, empty vector control. Representative for three biologically independent experiments.

(E) Multiple sequence alignment highlighting the conservation of active-site residues in DarT1-associated NADARs. Residues mechanistically important for guanine-ADPr hydrolysis are highlighted in pink, and residues important for DarT1 toxicity rescue are highlighted in green. The overall sequence identity of selected species in relation to *E. coli* C7 NADAR is between 48% and 57%.

DarT-NADAR TA operon with an antitoxin that is capable of reversing guanine ADP-ribosylation via its NADAR domain (Figure 6). The NADARs thus represent the third superfamily (after macrodomains and ARH proteins) of enzymes that can reverse ADP-ribosylation in full, in contrast to the NUDIX and ENPP1 family members, which both cleave at the ADPr pyrophosphate bond and remove AMP while leaving a phosphoribosyl moiety on the target.[36,37] Since other, non-DarT1-associated and phylogenetically unrelated NADARs such as that in *P. nicotianae* var. *parasitica* can also hydrolyze guanine-ADP-ribosyl moieties, this function seems to be evolutionarily conserved among members of different NADAR classes. Indeed, the wide conservation of catalytic residues through the NADAR superfamily supports this notion. The only NADAR superfamily member that has been characterized previously (*E. coli* YbiA) is involved in riboflavin biosynthesis in bacteria by cleaving the *N*-glycosidic bond of reactive riboflavin intermediates.[31] It is thus conceivable that for the isolated and non-DarT1-associated NADARs the physiological targets differ from ADP-ribosylated guanines (potentially including other nucleobases and small molecules) and remain unknown. Crystal structures of NADAR domains in ligand-free and ADPr substrate-bound states give insights into

	Guanine		Thymine
DNA base ADP-ribosylation			
Writers	Pierisin 1/2, Scabin, ScARP, CARP-1	DarT1	DarT2
ART family	ARTC	ARTD	ARTD
Erasers	DarG1 and other NADARs*		DarG2
Domain family	YbiA-like		Macrodomain
TA system	No	Yes DarTG1	Yes DarTG2

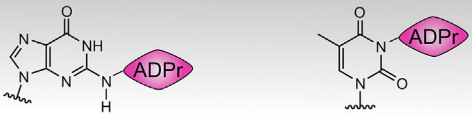

Figure 6. Overview of writers and erasers of DNA base ADP-ribosylation

*We note that while it was shown that Pierisin 1/2,[15,40] Scabin[13] and CARP-1[41] link ADP-ribose to the N2 exocyclic amine of the guanine (as does ScARP and DarT1), our assumption that NADARs function as erasers of these modifications is only based on the fact that NADARs are hydrolytically active on N2-guanine-ADPr linkages and is not experimentally confirmed.

general substrate recognition by NADARs. Based on the interactions between the protein and the ADPr ligand, the NADAR family seems to have evolved a pocket for specific ADPr recognition but with seemingly high flexibility to also recognize nucleoside derivatives such as riboflavin intermediates. Furthermore, it appears that NADARs have evolved features to function as antitoxins counteracting DarT1 activity. Structural differences including an extension of the YbiA-like core, a potential DNA binding surface, and minor arrangements of active-site residues are evident, and further studies are needed to fully understand their mechanistic relevance for DarT1-associated NADARs to counteract DarT toxicity. This could also contribute toward the development of chemical inhibitors of NADARs, which are promising drug targets given their essentiality for bacterial growth in the presence of DarT1 and the prevalence of DarTG1 in many pathogens (as DarTG2) including *Vibrio cholerae*, *Acinetobacter baumanii*, or *Salmonella enterica*. Furthermore, their absence in mammals could be advantageous in reducing the likelihood of off-target effects of drug candidates. Similarly, *Phytophthora* includes a plethora of plant pathogens causing severe agricultural damage to a wide range of host plants such as tobacco,[38] and famously potato, causing the Great Famine in Ireland,[39] so that characterization of its NADAR domain as a potential drug target is very likely worthwhile.

Our structural and biochemical studies on DarT1 revealed that (1) guanine ADP-ribosylation can also be catalyzed by ARTD family members, (2) guanine ADP-ribosylation is reversible, and (3) DarT1 and ScARP establish the same guanine-ADPr linkage, but through distinct molecular mechanisms (Figures 6 and S6). So far, guanosine base ADP-ribosylation has only been known to be catalyzed by members of the ARTC subfamily, including pierisins, ScARP, and CARP-1. Thus, DarT1 as an ARTD member close to the PARP family extends the repertoire of ART reactions catalyzed by the ARTD subfamily. This represents another striking example of the evolution of a conserved protein fold to develop different substrate specificities; in the case of DarT, this includes base modifications on thymidine and guanosine nucleotides. Furthermore, NADARs are the first characterized examples of enzymes capable of hydrolyzing the guanine-ADPr linkage, challenging the previously held assumption of the irreversibility of guanine ADP-ribosylation, which was also believed to be the underlying cause of its toxicity.[12,42] Identification of DarT1 as a guanine-specific ART prompted investigation of the TA operon-associated antitoxin enzyme, which was indeed able to reverse both DarT1- and ScARP-catalyzed ADPr transfer reactions. While NADARs function as antitoxins in DarTG1 systems, organisms expressing ARTC guanosine ARTs may encode other, non-DarT1-associated NADAR family members to protect the organism from toxic effects. It would be of further interest to clarify whether vertebrates also encode enzymes that can reverse DarT1-catalyzed ADP-ribosylation, as human TARG1 was identified to protect from DarT2 activity.[43] Finally, DarT1 produces the same guanine ADP-ribosylation product as ScARP and related toxins, yet it seems to employ a different mechanism for catalyzing the N2-C1 linkage. DarT1 and ScARP (Figure S6) differ in their overall structural makeup, in particular regarding the NAD^+-binding and the ARTT substrate recognition loop, generally suggesting they have different physiological targets. However, both share the NAD^+ binding mode as well as the guanosine substrate recognition and positioning through N2 and N3 coordination with an amide group (using glutamine in ScARP and asparagine in DarT1). Although modeled into the DarT1 structure, the transferase-characteristic glutamate is close to the catalytic center in both enzymes and there may be only minor differences in their relative positions concerning the distal-ribose and the N2 for linkage. This may influence reactivity of ScARP, requiring only the glutamate for catalysis. However, DarT1 seems to have evolved very high reaction efficiency by involving several additional residues in the catalytic mechanism. The higher toxicity of DarT1 compared to ScARP is evidenced by stronger bacteriostatic effects, requiring for instance tight expression-controlled vector systems for protein production of DarT1 and any crystallographic work performed with the glutamate mutant of DarT1. The set of essential catalytic residues is furthermore comparable between DarT1 and DarT2, making them equally efficient enzymes catalyzing base ADP-ribosylation. Of note, DarT1 and DarT2 reveal several structural differences, which allows them to act as specific guanine and thymine ARTs, respectively (Figure S5). This includes the NAD^+-binding loop, which lacks the prominent α helix and furthermore harbors, in DarT1, an additional catalytic aspartate potentially involved in proton transfer together with the conserved arginine. The latter is sufficient in

DarT2 for proton abstraction from the thymine in-ring nitrogen, yet also displays more side-chain flexibility among pre- and post-reaction states not observed in DarT1, possibly due to the presence and function of its catalytic aspartate. Moreover, DarT1 and DarT2 adapted the base-recognizing residues that are ideal for coordinating their targets, i.e., an asparagine for bridging to the in-ring nitrogen N3 and the amino group of the guanine in DarT1 and a histidine for hydrogen-bonding the thymine carbonyl oxygen in DarT2. Both of these residues that interact with the active-site base are located within the ARTT substrate binding loops, which coordinate the ssDNA strands in similar positions but do not share a common scaffold or base interactions. While DarT2 shows strong sequence specificity for a four-base motif, typically with thymine in the first and third position, we could not identify a consensus motif in *E. coli* C7 DarT1 substrates. Of note, both base-coordinating residues are in a structural position that is generally relevant for substrate binding of ARTs or that assist with the catalytic mechanism,[24] and we suggest considering those residues generally along with the ART-class-defining motifs ([H-Y-E] for ARTD and [R-S-E] for ARTC family members) to obtain insights into mechanism, substrate specificity, and function of the ART of interest. With DarT1, DarT2, and all other transferases characterized so far,[30,34,44] it becomes clear that a variety of ARTs have evolved and adapted in lower organisms for catalyzing ADP-ribosylation of different substrates but with principles also conserved in higher organisms. DarT transferases can be found linked in operons with different hydrolases,[30] and we predict that their characterization will reveal a plethora of unknown functions of ARTs and their associated enzymes, such as NADARs, in NAD^+-derived metabolism, nucleic acid processing, and beyond.

Limitations of the study

In this manuscript, we present the first biochemical characterization of the NADAR enzyme. However, further studies are needed to establish the exact physiological substrates and relevance of NADARs, particularly for NADARs that are not contained within an operon with DarT1. For the DarT1-associated NADARs, it still needs to be clarified how these enzymes recognize their respective ADP-ribosylated targets, whether they possess a certain sequence specificity, and whether potentially other DNA modifications are recognized and modulate NADAR activity. Furthermore, there are certain limitations of the current assay format used to assess the enzymatic activity of DarT and NADAR wild-type and mutant proteins. We notice in our studies that the toxicity survival assays and *in vitro* ADP-ribosylation assays appear to differ in sensitivity of the readout, resulting in minor discrepancies in the observed biochemical effects and survival phenotypes. It needs to be considered that there is the possibility of other mechanisms coming into play that are unrelated to DNA ADP-ribosylation and influence the readout, such as differences in protein folding and stability *in vitro* versus in cells. The *in vitro* ADP-ribosylation gel shift assay in its current set-up also only measures outcomes at a specific time point and therefore might not accurately capture the reaction kinetics. In future studies, a more sensitive assay could be developed and employed for addressing these limitations.

STAR★METHODS

Detailed methods are provided in the online version of this paper and include the following:

- KEY RESOURCES TABLE
- RESOURCE AVAILABILITY
  - Lead contact
  - Material availability
  - Data and code availability
- EXPERIMENTAL MODEL AND STUDY PARTICIPANT DETAILS
- METHOD DETAILS
  - Materials, reagents and chemicals
  - Constructs
  - Recombinant DarT protein expression and purification
  - Toxicity assays
  - ADP-ribosylation activity assays
  - Detection of ADP-ribosylated genomic DNA
  - Protein crystallization and data collection
- QUANTIFICATION AND STATISTICAL ANALYSIS

SUPPLEMENTAL INFORMATION

Supplemental information can be found online at https://doi.org/10.1016/j.molcel.2023.06.013.

ACKNOWLEDGMENTS

We thank Edward Lowe for help with X-ray diffraction data collection and Rachel Exley for helpful discussions; Bara Cihlova is acknowledged for help with biochemical experiments; we thank Professor Philip Poole for supporting the *in vivo* studies. We are grateful to Diamond Light Source for access to and assistance at beamline I03 throughout the project (proposal number mx23459). P.M. and A.M. were supported by the Croatian Science Foundation (project no. IP-2016-06-4242). Work in the Ahel Laboratory is supported by the Biotechnology and Biological Sciences Research Council (BB/R007195/1 and BB/W016613/1), the Wellcome Trust (210634 and 223107), and Cancer Research United Kingdom (C35050/A22284).

AUTHOR CONTRIBUTIONS

I.A. conceived the project and conceptualized experiments with input from M.S., A.M., and C.T.; M.S. conducted biochemical and crystallographic studies including the structure and data analysis/interpretation; R.R., P.M., Y.Z., and R.L. assisted with protein purification and with biochemical and *in vivo* assays; J.G.M.R. cloned *Phytophthora* NADAR; A.A. refined structural data and deposited data into the PDB; M.S. and I.A. wrote the manuscript with the support of all other authors.

DECLARATION OF INTERESTS

The authors declare no competing interests.

Received: November 10, 2022
Revised: April 13, 2023
Accepted: June 8, 2023
Published: June 29, 2023

REFERENCES

1. Weigele, P., and Raleigh, E.A. (2016). Biosynthesis and Function of Modified Bases in Bacteria and Their Viruses. Chem. Rev. *116*, 12655–12687. https://doi.org/10.1021/acs.chemrev.6b00114.

2. Johnson, T.B., and Coghill, R.D. (1925). Researches on pyrimidines. C111. The discovery of 5-methyl-cytosine in tuberculinic acid, the nucleic acid of the tubercle bacillus. J. Am. Chem. Soc. *47*, 2838–2844. https://doi.org/10.1021/ja01688a030.
3. Groslambert, J., Prokhorova, E., and Ahel, I. (2021). ADP-ribosylation of DNA and RNA. DNA Repair *105*, 103144. https://doi.org/10.1038/326223c0.
4. Challa, S., Stokes, M.S., and Kraus, W.L. (2021). Marts and marylation in the cytosol: Biological functions, mechanisms of action, and therapeutic potential. Cells *10*, 313–321. https://doi.org/10.3390/cells10020313.
5. Eleazer, R., and Fondufe-Mittendorf, Y.N. (2021). The multifaceted role of PARP1 in RNA biogenesis. Wiley Interdiscip. Rev. RNA *12*, e1617–e1621. https://doi.org/10.1002/wrna.1617.
6. Mikolčević, P., Hloušek-Kasun, A., Ahel, I., and Mikoč, A. (2021). ADP-ribosylation systems in bacteria and viruses. Comput. Struct. Biotechnol. J. *19*, 2366–2383. https://doi.org/10.1016/j.csbj.2021.04.023.
7. Palazzo, L., Mikolčević, P., Mikoč, A., and Ahel, I. (2019). ADP-ribosylation signalling and human disease. Open Biol. *9*, 190041. https://doi.org/10.1098/rsob.190041.
8. Barkauskaite, E., Jankevicius, G., and Ahel, I. (2015). Structures and Mechanisms of Enzymes Employed in the Synthesis and Degradation of PARP-Dependent Protein ADP-Ribosylation. Mol. Cell *58*, 935–946. https://doi.org/10.1016/j.molcel.2015.05.007.
9. Vyas, S., Matic, I., Uchima, L., Rood, J., Zaja, R., Hay, R.T., Ahel, I., and Chang, P. (2014). Family-wide analysis of poly(ADP-ribose) polymerase activity. Nat. Commun. *5*, 4426. https://doi.org/10.1038/ncomms5426.
10. Weixler, L., Schäringer, K., Momoh, J., Lüscher, B., Feijs, K.L.H., and Žaja, R. (2021). ADP-ribosylation of RNA and DNA: From in vitro characterization to in vivo function. Nucleic Acids Res. *49*, 3634–3650. https://doi.org/10.1093/nar/gkab136.
11. Yoshida, T., and Tsuge, H. (2018). Substrate N2 atom recognition mechanism in pierisin family DNA-targeting, guanine-specific ADP-ribosyltransferase ScARP. J. Biol. Chem. *293*, 13768–13774. https://doi.org/10.1074/jbc.AC118.004412.
12. Nakano, T., Takahashi-Nakaguchi, A., Yamamoto, M., and Watanabe, M. (2015). Pierisins and CARP-1: ADP-Ribosylation of DNA by ARTCs in Butterflies and Shellfish. In Endogenous ADP-Ribosylation, F. Koch-Nolte, ed. (Springer International Publishing), pp. 127–149. https://doi.org/10.1007/82_2014_416.
13. Lyons, B., Lugo, M.R., Carlin, S., Lidster, T., and Merrill, A.R. (2018). Characterization of the catalytic signature of Scabin toxin, a DNA-targeting ADP-ribosyltransferase. Biochem. J. *475*, 225–245. https://doi.org/10.1042/BCJ20170818.
14. Oda, T., Hirabayashi, H., Shikauchi, G., Takamura, R., Hiraga, K., Minami, H., Hashimoto, H., Yamamoto, M., Wakabayashi, K., Shimizu, T., and Sato, M. (2017). Structural basis of autoinhibition and activation of the DNA-targeting ADP-ribosyltransferase pierisin-1. J. Biol. Chem. *292*, 15445–15455. https://doi.org/10.1074/jbc.M117.776641.
15. Takamura-Enya, T., Watanabe, M., Totsuka, Y., Kanazawa, T., Matsushima-Hibiya, Y., Koyama, K., Sugimura, T., and Wakabayashi, K. (2001). Mono(ADP-ribosyl)ation of 2′-deoxyguanosine residue in DNA by an apoptosis-inducing protein pierisin-1 from cabbage butterfly. Proc. Natl. Acad. Sci. USA *98*, 12414–12419. https://doi.org/10.1073/pnas.221444598.
16. Belousova, E.A., Ishchenko, A.A., and Lavrik, O.I. (2018). DNA is a New Target of Parp3. Sci. Rep. *8*, 4176. https://doi.org/10.1038/s41598-018-22673-3.
17. Zarkovic, G., Belousova, E.A., Talhaoui, I., Saint-Pierre, C., Kutuzov, M.M., Matkarimov, B.T., Biard, D., Gasparutto, D., Lavrik, O.I., and Ishchenko, A.A. (2018). Characterization of DNA ADP-ribosyltransferase activities of PARP2 and PARP3: New insights into DNA ADP-ribosylation. Nucleic Acids Res. *46*, 2417–2431. https://doi.org/10.1093/nar/gkx1318.
18. Munnur, D., and Ahel, I. (2017). Reversible mono-ADP-ribosylation of DNA breaks. FEBS J. *284*, 4002–4016. https://doi.org/10.1111/febs.14297.
19. Munnur, D., Bartlett, E., Mikolčević, P., Kirby, I.T., Rack, J.G.M., Mikoč, A., Cohen, M.S., and Ahel, I. (2019). Reversible ADP-ribosylation of RNA. Nucleic Acids Res. *47*, 5658–5669. https://doi.org/10.1093/nar/gkz305.
20. Talhaoui, I., Lebedeva, N.A., Zarkovic, G., Saint-Pierre, C., Kutuzov, M.M., Sukhanova, M.v., Matkarimov, B.T., Gasparutto, D., Saparbaev, M.K., Lavrik, O.I., and Ishchenko, A.A. (2016). Poly(ADP-ribose) polymerases covalently modify strand break termini in DNA fragments in vitro. Nucleic Acids Res. *44*, 9279–9295. https://doi.org/10.1093/nar/gkw675.
21. Jurėnas, D., Payelleville, A., Roghanian, M., Turnbull, K.J., Givaudan, A., Brillard, J., Hauryliuk, V., and Cascales, E. (2021). Photorhabdus antibacterial Rhs polymorphic toxin inhibits translation through ADP-ribosylation of 23S ribosomal RNA. Nucleic Acids Res. *49*, 8384–8395. https://doi.org/10.1093/nar/gkab608.
22. Bullen, N.P., Sychantha, D., Thang, S.S., Culviner, P.H., Rudzite, M., Ahmad, S., Shah, V.S., Filloux, A., Prehna, G., and Whitney, J.C. (2022). An ADP-ribosyltransferase toxin kills bacterial cells by modifying structured non-coding RNAs. Mol. Cell *82*, 3484–3498.e11. https://doi.org/10.1016/j.molcel.2022.08.015.
23. Jankevicius, G., Ariza, A., Ahel, M., and Ahel, I. (2016). The toxin-antitoxin system DarTG catalyzes reversible ADP-ribosylation of DNA. Mol. Cell *64*, 1109–1116. https://doi.org/10.1016/j.molcel.2016.11.014.
24. Schuller, M., Butler, R.E., Ariza, A., Tromans-Coia, C., Jankevicius, G., Claridge, T.D.W., Kendall, S.L., Goh, S., Stewart, G.R., and Ahel, I. (2021). Molecular basis for DarT ADP-ribosylation of a DNA base. Nature *596*, 597–602. https://doi.org/10.1038/s41586-021-03825-4.
25. Singh, G., Yadav, M., Ghosh, C., and Rathore, J.S. (2021). Bacterial toxin-antitoxin modules: classification, functions, and association with persistence. Curr. Res. Microb. Sci. *2*, 100047. https://doi.org/10.1016/j.crmicr.2021.100047.
26. Lawarée, E., Jankevicius, G., Cooper, C., Ahel, I., Uphoff, S., and Tang, C.M. (2020). DNA ADP-Ribosylation Stalls Replication and Is Reversed by RecF-Mediated Homologous Recombination and Nucleotide Excision Repair. Cell Rep. *30*, 1373–1384.e4. https://doi.org/10.1016/j.celrep.2020.01.014.
27. Zaveri, A., Wang, R., Botella, L., Sharma, R., Zhu, L., Wallach, J.B., Song, N., Jansen, R.S., Rhee, K.Y., Ehrt, S., and Schnappinger, D. (2020). Depletion of the DarG antitoxin in Mycobacterium tuberculosis triggers the DNA-damage response and leads to cell death. Mol. Microbiol. *114*, 641–652. https://doi.org/10.1111/mmi.14571.
28. LeRoux, M., Srikant, S., Teodoro, G.I.C., Zhang, T., Littlehale, M.L., Doron, S., Badiee, M., Leung, A.K.L., Sorek, R., and Laub, M.T. (2022). The DarTG toxin-antitoxin system provides phage defence by ADP-ribosylating viral DNA. Nat. Microbiol. *7*, 1028–1040. https://doi.org/10.1038/s41564-022-01153-5.
29. Makarova, K.S., Wolf, Y.I., Snir, S., and Koonin, E.v. (2011). Defense Islands in Bacterial and Archaeal Genomes and Prediction of Novel Defense Systems. J. Bacteriol. *193*, 6039–6056. https://doi.org/10.1128/JB.05535-11.
30. de Souza, R.F., and Aravind, L. (2012). Identification of novel components of NAD-utilizing metabolic pathways and prediction of their biochemical functions. Mol. Biosyst. *8*, 1661–1677. https://doi.org/10.1039/c2mb05487f.
31. Frelin, O., Huang, L., Hasnain, G., Jeffryes, J.G., Ziemak, M.J., Rocca, J.R., Wang, B., Rice, J., Roje, S., Yurgel, S.N., et al. (2015). A directed-overflow and damage-control N-glycosidase in riboflavin biosynthesis. Biochem. J. *466*, 137–145. https://doi.org/10.1042/BJ20141237.
32. Inoue, T., Shingaki, R., Hirose, S., Waki, K., Mori, H., and Fukui, K. (2007). Genome-wide screening of genes required for swarming motility in Escherichia coli K-12. J. Bacteriol. *189*, 950–957. https://doi.org/10.1128/JB.01294-06.
33. Nakano, T., Matsushima-Hibiya, Y., Yamamoto, M., Takahashi-Nakaguchi, A., Fukuda, H., Ono, M., Takamura-Enya, T., Kinashi, H., and Totsuka, Y. (2013). ADP-ribosylation of guanosine by SCO5461 protein secreted from Streptomyces coelicolor. Toxicon *63*, 55–63. https://doi.org/10.1016/j.toxicon.2012.11.019.

34. Aravind, L., Zhang, D., de Souza, R.F., Anand, S., and Iyer, L.M. (2015). The Natural History of ADP-Ribosyltransferases and the ADP-Ribosylation System. In Endogenous ADP-Ribosylation, F. Koch-Nolte, ed. (Springer International Publishing), pp. 3–32. https://doi.org/10.1007/82_2014_414.

35. Jørgensen, R., Wang, Y., Visschedyk, D., and Merrill, A.R. (2008). The nature and character of the transition state for the ADP-ribosyltransferase reaction. EMBO Rep. *9*, 802–809. https://doi.org/10.1038/embor.2008.90.

36. Palazzo, L., Daniels, C.M., Nettleship, J.E., Rahman, N., McPherson, R.L., Ong, S.E., Kato, K., Nureki, O., Leung, A.K.L., and Ahel, I. (2016). ENPP1 processes protein ADP-ribosylation in vitro. FEBS J. *283*, 3371–3388. https://doi.org/10.1111/febs.13811.

37. Palazzo, L., Thomas, B., Jemth, A.S., Colby, T., Leidecker, O., Feijs, K.L.H., Zaja, R., Loseva, O., Puigvert, J.C., Matic, I., et al. (2015). Processing of protein ADP-ribosylation by Nudix hydrolases. Biochem. J. *468*, 293–301. https://doi.org/10.1042/BJ20141554.

38. Meng, Y., Zhang, Q., Ding, W., and Shan, W. (2014). Phytophthora parasitica: a model oomycete plant pathogen. Mycology *5*, 43–51. https://doi.org/10.1080/21501203.2014.917734.

39. Austin Bourke, P.M. (1964). Emergence of potato blight, 1843-46. Nature *203*, 805–808. https://doi.org/10.1038/203805a0.

40. Takamura-Enya, T., Watanabe, M., Koyama, K., Sugimura, T., and Wakabayashi, K. (2004). Mono(ADP-ribosyl)ation of the N 2 amino groups of guanine residues in DNA by pierisin-2, from the cabbage butterfly, Pieris brassicae. Biochem. Biophys. Res. Commun. *323*, 579–582. https://doi.org/10.1016/j.bbrc.2004.08.132.

41. Nakano, T., Matsushima-Hibiya, Y., Yamamoto, M., Enomoto, S., Matsumoto, Y., Totsuka, Y., Watanabe, M., Sugimura, T., and Wakabayashi, K. (2006). Purification and molecular cloning of a DNA ADP-ribosylating protein, CARP-1, from the edible clam Meretrix lamarckii. Proc. Natl. Acad. Sci. USA *103*, 13652–13657. https://doi.org/10.1073/pnas.0606140103.

42. Kono, T., Watanabe, M., Koyama, K., Kishimoto, T., Fukushima, S., Sugimura, T., and Wakabayashi, K. (1999). Cytotoxic activity of pierisin, from the cabbage butterfly, Pieris rapae, in various human cancer cell lines. Cancer Lett. *137*, 75–81. https://doi.org/10.1016/S0304-3835(98)00346-2.

43. Tromans-Coia, C., Sanchi, A., Moeller, G.K., Timinszky, G., Lopes, M., and Ahel, I. (2021). TARG1 protects against toxic DNA ADP-ribosylation. Nucleic Acids Res. *49*, 10477–10492. https://doi.org/10.1093/nar/gkab771.

44. Baysarowich, J., Koteva, K., Hughes, D.W., Ejim, L., Griffiths, E., Zhang, K., Junop, M., and Wright, G.D. (2008). Rifamycin antibiotic resistance by ADP-ribosylation: Structure and diversity of Arr. Proc. Natl. Acad. Sci. USA *105*, 4886–4891. https://doi.org/10.1073/pnas.0711939105.

45. Guzman, L.M., Weiss, D.S., and Beckwith, J. (1997). Domain-swapping analysis of FtsI, FtsL, and FtsQ, bitopic membrane proteins essential for cell division in Escherichia coli. J. Bacteriol. *179*, 5094–5103. https://doi.org/10.1128/jb.179.16.5094-5103.1997.

46. Savitsky, P., Bray, J., Cooper, C.D.O., Marsden, B.D., Mahajan, P., Burgess-Brown, N.A., and Gileadi, O. (2010). High-throughput production of human proteins for crystallization: The SGC experience. J. Struct. Biol. *172*, 3–13. https://doi.org/10.1016/j.jsb.2010.06.008.

47. Lalić, J., Posavec Marjanović, M., Palazzo, L., Perina, D., Sabljić, I., Žaja, R., Colby, T., Pleše, B., Halasz, M., Jankevicius, G., et al. (2016). Disruption of macrodomain protein SCO6735 increases antibiotic production in streptomyces coelicolor. J. Biol. Chem. *291*, 23175–23187. https://doi.org/10.1074/jbc.M116.721894.

48. Mirdita, M., Schütze, K., Moriwaki, Y., Heo, L., Ovchinnikov, S., and Steinegger, M. (2022). ColabFold: making protein folding accessible to all. Nat. Methods *19*, 679–682. https://doi.org/10.1038/s41592-022-01488-1.

49. Winter, G. (2010). *xia2*: an expert system for macromolecular crystallography data reduction. J. Appl. Crystallogr. *43*, 186–190. https://doi.org/10.1107/S0021889809045701.

50. Storoni, L.C., McCoy, A.J., and Read, R.J. (2004). Likelihood-enhanced fast rotation functions. Acta Crystallogr. D Biol. Crystallogr. *60*, 432–438. https://doi.org/10.1107/S0907444903028956.

51. Emsley, P., and Cowtan, K. (2004). Coot: Model-building tools for molecular graphics. Acta Crystallogr. D Biol. Crystallogr. *60*, 2126–2132. https://doi.org/10.1107/S0907444904019158.

52. Murshudov, G.N., Vagin, A.A., and Dodson, E.J. (1997). Refinement of macromolecular structures by the maximum-likelihood method. Acta Crystallogr. D Biol. Crystallogr. *53*, 240–255. https://doi.org/10.1107/S0907444996012255.

53. Waterhouse, A.M., Procter, J.B., Martin, D.M.A., Clamp, M., and Barton, G.J. (2009). Jalview Version 2-A multiple sequence alignment editor and analysis workbench. Bioinformatics *25*, 1189–1191. https://doi.org/10.1093/bioinformatics/btp033.

54. Huson, D.H. (1998). SplitsTree: Analyzing and visualizing evolutionary data. Bioinformatics *14*, 68–73. https://doi.org/10.1093/bioinformatics/14.1.68.

55. McGuffin, L.J., Bryson, K., and Jones, D.T. (2000). The PSIPRED protein structure prediction server. Bioinformatics *16*, 404–405. https://doi.org/10.1093/bioinformatics/16.4.404.

56. Ashkenazy, H., Abadi, S., Martz, E., Chay, O., Mayrose, I., Pupko, T., and Ben-Tal, N. (2016). ConSurf 2016: an improved methodology to estimate and visualize evolutionary conservation in macromolecules. Nucleic Acids Res. *44*, W344–W350. https://doi.org/10.1093/nar/gkw408.

57. Cowtan, K. (2010). Recent developments in classical density modification. Acta Crystallogr. D Biol. Crystallogr. *66*, 470–478. https://doi.org/10.1107/S090744490903947X.

58. Chen, V.B., Arendall, W.B., Headd, J.J., Keedy, D.A., Immormino, R.M., Kapral, G.J., Murray, L.W., Richardson, J.S., and Richardson, D.C. (2010). MolProbity: all-atom structure validation for macromolecular crystallography. Acta crystallogr *66*, 12–21. https://doi.org/10.1107/S0907444909042073.

59. Katoh, K., Rozewicki, J., and Yamada, K.D. (2019). MAFFT online service: Multiple sequence alignment, interactive sequence choice and visualization. Brief. Bioinform. *20*, 1160–1166. https://doi.org/10.1093/bib/bbx108.

60. Saitou, N., and Nei, M. (1987). The neighbor-joining method: a new method for reconstructing phylogenetic trees. Mol. Biol. Evol. *4*, 406–425. https://doi.org/10.1093/oxfordjournals.molbev.a040454.

STAR★METHODS

KEY RESOURCES TABLE

REAGENT or RESOURCE	SOURCE	IDENTIFIER
Antibodies		
Rabbit Poly/Mono-ADP Ribose	Cell Signaling Technology	E6F6A; RRID: AB_2749858
Mouse autoanti-dsDNA	DSHB	AB_10805293; RRID: AB_10805293
Goat anti-rabbit	Dako-Agilent	P0399; RRID: AB_2617141
Goat anti-mouse	Dako-Agilent	P0447; RRID:AB_2617137
Bacterial and virus strains		
DH5α (*huA2 a(argF-lacZ)U169 phoA glnV44 a80a(lacZ)M15 gyrA96 recA1 relA1 endA1 thi-1 hsdR17*)	NEB	Cat#: C2987
DH5α-macro (DH5α with integrated *T. aquaticus* DarG macrodomain at P21 site)	Schuller et al., 2021[24]	N/A
BL21 (*fhuA2 [lon] ompT gal [dcm] ΔhsdS*)	NEB	Cat#: C2530
BL21(DE3) (*fhuA2 [lon] ompT gal (λ DE3) [dcm] ΔhsdS λ DE3 = λ sBamHIo ΔEcoRI-B int::(lacI::PlacUV5::T7 gene1) i21 Δnin5*)	NEB	Cat#: C2527
Rosetta™ (DE3) (*F-ompT hsdSB(rB- mB-) gal dcm (DE3) pRARE* (cam^R))	Novagen	Cat#: 70954
Biological samples		
Genomic DNA of *P. nicotianae* var. *parasitica* strain IMI403522	CABI	N/A
Chemicals, peptides, and recombinant proteins		
β-NAD^+	Roche-Merck	Cat#: 10127965001
Carba-NAD^+	Hangzhou YiLu Biological technology	Cas#: 112345-60-5
ADP-ribose	Sigma Aldrich-Merck	Cat#: A0752
Crystallization Screen: Clear Strategy 2	Molecular Dimensions	Cat#: MD1-15
Crystallization Screen: Structure Screen 1 + 2	Molecular Dimensions	Cat#: MD1-30
SYBR Gold Nucleic Acid Gel Stain	Invitrogen	Cat#: S11494
Critical commercial assays		
QuikChange Lightning Site-Directed Mutagenesis Kit	Agilent	Cat#: 210518
NEBuilder HiFi DNA Assembly Master Mix	NEB	Cat#: E2621
Pierce ECL Western Blotting Substrate	Thermo Scientific	Cat#: 32106
Deposited data		
E. coli C7 DarT1: NAD^+ structure	This paper	PDB: 8BAQ
E. coli C7 DarT1: ADP-ribosylated DNA structure	This paper	PDB: 8BAR
E. coli C7 DarT1: Carba-NAD^+ and DNA structure	This paper	PDB: 8BAS
G. lovleyi NADAR apo structure	This paper	PDB: 8BAT
P. nicotianae var. *parasitica* NADAR:ADPr structure	This paper	PDB: 8BAU
Coding sequence of *P. nicotianae* var. *parasitica* NADAR	This paper	GenBank#: OP425850
Uncropped gel images and toxicity assay results	This paper	Mendeley: https://doi.org/10.17632/fzrbk79x5f.1
Oligonucleotides		
Primer: DarT-ADPr-27mer Forward: CACGACACGAGCAGGCATGTCCACGTG	This paper	N/A
Primer: DarT-ADPr-27mer-rc Forward: CACGTGGACATGCCTGCTCGTGTCGTG	This paper	N/A

(*Continued on next page*)

CellPress
OPEN ACCESS

Continued

REAGENT or RESOURCE	SOURCE	IDENTIFIER
Primer: PolyT-G<br>Forward: TTTTTTGTTTTTTTTTTTT	This paper	N/A
Primer: PolyT-GG<br>Forward: TTTTTTGGTTTTTTTTTTT	This paper	N/A
Primer: PolyT-GTG<br>Forward: TTTTTTGTGTTTTTTTTTT	This paper	N/A
Primer: PolyT-GTTG<br>Forward: TTTTTTGTTGTTTTTTTTT	This paper	N/A
Primer: PolyT-GTTTG<br>Forward: TTTTTTGTTTGTTTTTTTT	This paper	N/A
Primer: PolyT-GTTTTG<br>Forward: TTTTTTGTTTTGTTTTTTT	This paper	N/A
Primer: DarT_cryst<br>Forward: AAGAC	This paper	N/A
Additional primers for assays, cloning and site-directed mutagenesis of DarT1 and NADARs, see Supp. Table S3	This paper	N/A
Recombinant DNA		
pBAD33 (Medium copy plasmid with an arabinose-inducible promoter; cam^R)	Guzman et al., 1995[45]	N/A
pET28a (Medium copy plasmid containing an IPTG-inducible promoter; kan^R)	Novagen	Cat#69864
pNIC28-Bsa4 (Medium copy plasmid containing an IPTG-inducible promoter; kan^R)	Addgene[46]	Cat#26103
pBAD33_Taq_*darT* (pBAD33 carrying *T. aquaticus* *darT2* full-length; cam^R)	Jankevicius et al., 2017 [23]	N/A
pET28_SC_*SCO5461* (pET28a carrying *S. coelicolor* *scarp (SCO5461)* full-length; kan^R)	Lalić, J. et al., 2016 [47]	N/A
pET28_Taq_*darG*_macro (pET28a carrying *T. aquaticus* *darG* macrodomain (aa 1–155); kan^R)	Jankevicius et al., 2017 [23]	N/A
pBAD33_Ecoli_*darT1* (pBAD33 carrying *E. coli* C7 *darT1* full-length; cam^R)	This paper	N/A
pBAD33_Ecoli_*darT1*^{F18A} (pBAD33 carrying *E. coli* C7 *darT1*^{F18A} full-length; cam^R)	This paper	N/A
pBAD33_Ecoli_*darT1*^{D49A} (pBAD33 carrying *E. coli* C7 *darT1*^{D49A} full-length; cam^R)	This paper	N/A
pBAD33_Ecoli_*darT1*^{R52A} (pBAD33 carrying *E. coli* C7 *darT1*^{R52A} full-length; cam^R)	This paper	N/A
pBAD33_Ecoli_*darT1*^{D54A} (pBAD33 carrying *E. coli* C7 *darT1*^{D54A} full-length; cam^R)	This paper	N/A
pBAD33_Ecoli_*darT1*^{S63A} (pBAD33 carrying *E. coli* C7 *darT1*^{S63A} full-length; cam^R)	This paper	N/A
pBAD33_Ecoli_*darT1*^{M71A} (pBAD33 carrying *E. coli* C7 *darT1*^{M71A} full-length; cam^R)	This paper	N/A
pBAD33_Ecoli_*darT1*^{F72A} (pBAD33 carrying *E. coli* C7 *darT1*^{F72A} full-length; cam^R)	This paper	N/A
pBAD33_Ecoli_*darT1*^{N104A} (pBAD33 carrying *E. coli* C7 *darT1*^{N104A} full-length; cam^R)	This paper	N/A
pBAD33_Ecoli_*darT1*^{E152A} (pBAD33 carrying *E. coli* C7 *darT1*^{E152A} full-length; cam^R)	This paper	N/A
pNIC28_Ecoli_*darT*^{E152A} (pNIC28-Bsa4 carrying *E. coli* C7 *darT*^{E152A} full-length; kan^R)	This paper	N/A
pBAD33_Glov_*darT1* (pBAD33 carrying *G. lovleyi* *darT1* full-length; cam^R)	This paper	N/A

(*Continued on next page*)

Continued		
REAGENT or RESOURCE	SOURCE	IDENTIFIER
pBAD33_Glov_*darT1*^{E152A} (pBAD33 carrying *G. lovleyi darT1*^{E152A} full-length; cam^{R})	This paper	N/A
pDEST17_Pnp_*nadar* (pDEST17 carrying *P. nicotianae* var. *parasitica nadar* full-length; kan^{R})	This paper	N/A
pET28_Glov_*nadar* (pET28a carrying *G. lovleyi nadar* full-length; kan^{R})	This paper	N/A
pET28_SinoR_*nadar* (pET28a carrying *S. fredii nadar* full-length; kan^{R})	This paper	N/A
pET28_Ecoli_*nadar* (pET28a carrying *E. coli* C7 *nadar* full-length; kan^{R})	This paper	N/A
pET28_Ecoli_*nadar*^{E88A} (pET28a carrying *E. coli* C7 *nadar*^{E88A} full-length; kan^{R})	This paper	N/A
pET28_Ecoli_*nadar*^{E88Q} (pET28a carrying *E. coli* C7 *nadar*^{E88Q} full-length; kan^{R})	This paper	N/A
pET28_Ecoli_*nadar*^{K95A} (pET28a carrying *E. coli* C7 *nadar*^{K95A} full-length; kan^{R})	This paper	N/A
pET28_Ecoli_*nadar*^{K95R} (pET28a carrying *E. coli* C7 *nadar*^{K95R} full-length; kan^{R})	This paper	N/A
pET28_Ecoli_*nadar*^{K115A} (pET28a carrying *E. coli* C7 *nadar*^{K115A} full-length; kan^{R})	This paper	N/A
pET28_Ecoli_*nadar*^{K116A} (pET28a carrying *E. coli* C7 *nadar*^{K116A} full-length; kan^{R})	This paper	N/A
pET28_Ecoli_*nadar*^{R119A} (pET28a carrying *E. coli* C7 *nadar*^{R119A} full-length; kan^{R})	This paper	N/A
pET28_Ecoli_*nadar*^{K121A} (pET28a carrying *E. coli* C7 *nadar*^{K121A} full-length; kan^{R})	This paper	N/A
pET28_Ecoli_*nadar*^{D171A} (pET28a carrying *E. coli* C7 *nadar*^{D171A} full-length; kan^{R})	This paper	N/A
pET28_Ecoli_*nadar*^{D171N} (pET28a carrying *E. coli* C7 *nadar*^{D171N}full-length; kan^{R})	This paper	N/A
pET28_Ecoli_*nadar*^{E173A} (pET28a carrying *E. coli* C7 *nadar*^{E173A} full-length; kan^{R})	This paper	N/A
pET28_Ecoli_*nadar*^{K178A} (pET28a carrying *E. coli* C7 *nadar*^{K178A} full-length; kan^{R})	This paper	N/A
Software and algorithms		
AlphaFold2 - Colaboratory	Mirdita, M. et al., 2022[48]	AlphaFold2_advanced.ipynb
XIA2-DIALS platform	Winter, G. et al., 2010[49]	https://www.ccp4.ac.uk/
PHASER	Storoni, L.C. et al., 2004[50]	https://www.ccp4.ac.uk/
COOT	Emsley, P., and Cowtan, K., 2004[51]	https://www.ccp4.ac.uk/
REFMAC5	Murshudov, G.N. et al., 1997[52]	https://www.ccp4.ac.uk/
PyMol v.2.3.3	Schrödinger, LLC	https://pymol.org/2/
JalView v2	Waterhouse, A.M. et al., 2009[53]	https://www.jalview.org/
SplitsTree4	Huson, D.H., 1998[54]	https://github.com/husonlab/splitstree4
PSIPRED 4.0	McGuffin, L.J. et al., 2000[55]	http://bioinf.cs.ucl.ac.uk/psipred/
ConSurf	Ashkenazy, H. et al., 2016[56]	http://consurf.tau.ac.il
Inkscape 1.2.1	Inkscape Project, 2020	https://inkscape.org
Other		
NCBI database and Basic Local Alignment Search Tool	NCBI	https://blast.ncbi.nlm.nih.gov/Blast.cgi

RESOURCE AVAILABILITY

Lead contact

Further information and requests for resources and reagents should be directed to and will be fulfilled by the lead contact, Ivan Ahel (ivan.ahel@path.ox.ac.uk).

Material availability

Plasmids generated in this study will be provided upon request to the lead contact.

Data and code availability

- All structures have been deposited in the PDB database (https://www.rcsb.org). The coding sequence of NADAR from *P. nicotianae* var. *parasitica* was deposited in GenBank. Uncropped gel images and toxicity assay results have been deposited at Mendeley Data and are publicly available as of the date of publication. Accession numbers and DOI are listed in the key resources table.
- This paper does not report any original code.
- Any additional information required to reanalyze the data reported in this paper is available from the lead contact upon request.

EXPERIMENTAL MODEL AND STUDY PARTICIPANT DETAILS

All *E. coli* strains used in this study were grown in Luria-Bertani (LB) broth (Fisher Scientific) supplemented with 25 μg/mL chloramphenicol to maintain pBAD33-based plasmids and 50 μg/mL kanamycin to maintain pET28a- and pNIC28-based plasmids. Bacteria which carry pBAD33 plasmids encoding toxin were additionally grown in the presence of 0.8% glucose to prevent toxin expression. Bacteria were grown at 37°C unless stated otherwise.

METHOD DETAILS

Materials, reagents and chemicals

High-fidelity DNA polymerase Phusion, Gibson Assembly and Gateway cloning reagents were obtained from New England Biolabs and Thermo Scientific. DNA primers and ssDNA substrates (Table S3) were synthesized by Thermo Scientific. Carba-NAD^+ was synthesized by Hangzhou YiLu Biological technology. Crystallization screens were procured from Molecular Dimensions. All remaining chemicals were purchased from Sigma unless stated otherwise.

Constructs

The gene fragment encoding full-length *E. coli* C7 $DarT1^{E152A}$ (residues 1–207) was synthesised by Thermo Scientific and cloned into a pNIC28-Bsa4 expression vector for protein crystallization and a pBAD33 expression vector for biochemical studies, both adding an N-terminal His_6-TEV cleavage site, by Gibson Assembly (NEB). Wild-type *E. coli* C7 DarT1 was obtained with site-directed mutagenesis of the constructed pBAD33 expression vector. *S. coelicolor* ScARP (SCO5461) was produced as previously described.[47] The coding sequence of full-length NADAR from *P. nicotianae* var. *parasitica* strain IMI403522 was amplified from genomic DNA obtained from CABI (Egham, UK) and cloned into pDEST17 via Gateway cloning. The forward primer contained the coding sequence for an HRV3C cleavage site, which allowed tag removal from the protein expressed from the resulting construct. The coding sequence was confirmed by Sanger sequencing of three independent clones and deposited in GenBank with the accession number OP425850. The full-length genes of *E. coli* C7 NADAR (residues 1–229), *G. lovleyi* [synonym: *Trichlorobacter lovleyi*] NADAR (residues 1–234) and *S. fredii* NADAR (residues 1–234) were synthesised and cloned into a pET28a vector by GenScript. Mutations were introduced using the QuikChange Lightning Site-Directed Mutagenesis Kit (Agilent). All plasmids were verified by Sanger sequencing. The constructs used in this study are summarised in Table S4.

Recombinant DarT protein expression and purification

To enable the crystallographic studies, *E. coli* C7 DarT1 was expressed and purified with the earlier described catalytic-null glutamate substitution E152A (corresponding to E160A in *T. aquaticus* DarT2)[23] to counteract the inherent toxicity of DarT. *E. coli* Rosetta strain BL21(DE3) was transformed with NADAR or *E. coli* C7 $DarT1^{E152A}$ constructs and grown at 37°C in Terrific Broth (Merck Millipore) supplemented with 50 μg/mL of kanamycin and 35 μg/mL of chloramphenicol. After reaching an OD_{600nm} of 1.0–1.2, the temperature was lowered to 18°C prior to induction of protein expression overnight (O/N) by adding 0.5 mM IPTG. Harvested cells were resuspended in lysis buffer (50 mM HEPES (pH 7.4), 500 mM NaCl, 5% glycerol, 20 mM imidazole, 0.5 mM TCEP, cOmplete EDTA-free protease inhibitors (Roche)) and stored at −20°C until purification.

For protein purification, pellets were gently thawed and lysed by high-pressure homogenisation. DNA was digested using Benzonase Nuclease (Merck Life Science). Proteins were purified by immobilised metal affinity chromatography (IMAC) using Ni-Sepharose resin (GE Healthcare) and eluted stepwise in binding buffer containing 40–500 mM imidazole. Typically, a high salt wash with 1 M

NaCl was combined with the first elution step including 40 mM imidazole. Removal of the hexahistidine tag was carried out by addition of recombinant TEV protease during O/N dialysis into buffer without imidazole, followed by purification on a second IMAC column and finally by size-exclusion chromatography (SEC) (Superdex 75, GE Healthcare) in a buffer consisting of 25 mM HEPES (pH 7.4), 300 mM NaCl, 5% glycerol and 0.5 mM TCEP.

For expression and purification of wild-type and mutant *E. coli* C7 DarT1 proteins for activity assays, the corresponding pBAD33 plasmids were transformed into "DH5α-macro" cells as described previously.[24] Cells were grown at 37°C in LB medium (Miller) supplemented with 25 μg/mL chloramphenicol and 0.8% (*w/v*) glucose to an OD_{600nm} of 0.8–1.0. Cells were then pelleted by centrifugation at 4000 x g for 15 min at RT and resuspended in fresh LB media containing 25 μg/mL chloramphenicol and 0.8% (*w/v*) arabinose to induce protein expression. After 2.0 h at 37°C, cells were harvested by centrifugation (4000 x g, 15 min) and resuspended in lysis buffer (50 mM TRIS (pH 8.0), 500 mM NaCl, 5% glycerol, 20 mM imidazole, 0.5 mM TCEP) and stored at −20°C until purification. Cells were lysed using BugBuster (Novagen) following the manufacturer's instructions after adding cOmplete EDTA-free protease inhibitors (Roche) and Benzonase Nuclease (Merck Life Science). The DarT1 proteins were purified by IMAC using Ni-Sepharose resin (GE Healthcare) and then dialyzed against protein storage buffer containing 25 mM HEPES (pH 7.4), 300 mM NaCl, 5% glycerol, 0.5 mM TCEP.

All proteins were characterized by SDS-PAGE, then flash frozen in liquid nitrogen and stored at −80°C until required. Protein concentrations were determined by measuring absorption of the sample at 280 nm with a DS-11 FX nanodrop (DeNovix).

Toxicity assays

BL21 cells were used for *E. coli* C7 DarT1 wild-type and mutant expression studies while BL21(DE3) cells were used for co-expression studies of *E. coli* C7 DarT1 and NADAR proteins. Cells were transformed with the respective constructs and selected O/N on LB agar plates in the presence of 0.8% (*w/v*) glucose and the appropriate antibiotics for selection using 25 μg/mL chloramphenicol and 50 μg/mL kanamycin. 3–5 colonies were picked and grown up in LB medium in the presence of 0.8% (*w/v*) glucose and the appropriate antibiotics until the cultures reached OD_{600nm} of ~0.5–0.8. The OD_{600nm} was then adjusted from all samples to 0.5 and 1:10 dilution series were prepared. 5 μL were then spotted onto LB agar plates containing the appropriate antibiotics for selection and 0.8% (*w/v*) glucose for repression or 0.8% (*w/v*) arabinose/50 μM IPTG for induction of protein expression, respectively. The bacteriostatic effects were assessed after incubating the plates at 37°C O/N.

ADP-ribosylation activity assays

Oligo ADP-ribosylation reactions were performed in buffer containing 50 mM TRIS-Cl (pH 8.0), 50 mM NaCl supplemented with 5 mM ETDA at 37°C for 30 min for *T. aquaticus* DarT2 and 60 min for *E. coli* C7 DarT1 and *S. coelicolor* ScARP in an assay volume of 10 μL. In general, 1 μM DarT protein was incubated with oligonucleotides at a concentration of 3 μM and β-NAD^+ in excess (500 μM) and 0.1 μM *S. coelicolor* ScARP protein was incubated with oligonucleotides at a concentration of 10 μM and β-NAD^+ in excess (3 mM). For following oligo de-modification by hydrolases, the ADP-ribosylation reaction was stopped by heating the samples for 15 min at 95°C. Then, samples were incubated with buffer as control or 1 μM of the indicated hydrolase at 37°C for 30 min (or 15 min where stated). Reaction products were analyzed by separation on denaturing polyacrylamide gels run in TBE buffer, after adding 10 μL urea loading dye (10 mM TRIS-Cl (pH 8.0), 10 mM EDTA, 4 M urea) to the ADP-ribosylation reactions and incubation for 3 min at 95°C. 10 μL of the samples were loaded onto the gel and oligos visualised under UV light (340 nm) after ethidium bromide-staining. PolyT-G oligos were visualised by SYBR Gold Nucleic Acid Gel Stain (Invitrogen). We note that if not stated otherwise negative (−) controls are samples which have been treated as all other samples and have buffer instead of toxin or hydrolase enzyme added to the reaction, thus showing the unmodified oligo for internal referencing to the ADPr-modified oligos. The positive (+) controls are samples which have also been treated as all other samples and have toxin but no hydrolyze enzyme added to the reaction, thus showing the ADPr-modified oligo for internal referencing to the unmodified oligos.

Detection of ADP-ribosylated genomic DNA

E. coli BL21 cells transformed with DarT1-encoding pBAD33 plasmids were grown to OD_{600nm} of 0.2–0.3 in LB containing 0.8% (*w/v*) glucose before protein expression was induced with 0.8% (*w/v*) arabinose. Cells were harvested by centrifugation (4000 x g, 3 min), washed with PBS, re-suspended in boiling lysis buffer (1.0% SDS, 10 mM TRIS-Cl, 1 mM EDTA, pH 8.0) and lysed by heating to 95°C for 5 min. Cell lysates were subjected to proteinase K treatment for 1 h, 50°C. gDNA was then extracted by phenol:chloroform: isoamyl alcohol extraction and recovered by ammonium acetate/ethanol precipitation. The DNA pellets were washed twice with 70% ethanol before re-suspending in TE buffer and concentration determination using a DS-11 FX nanodrop (DeNovix). 750 ng of gDNA was dotted onto a nitrocellulose membrane (Amersham Protran 0.45 NC nitrocellulose) and crosslinked with 1200 J using a Stratalinker UV crosslinker. Crosslinked DNA was then immunoblotted for gDNA (autoanti-dsDNA, DSHB, 1:200) or ADPr-gDNA (Poly/Mono-ADP Ribose, E6F6A, Cell Signaling Technology, 1:1000) for 1 h at RT in 5% (*w/v*) powdered milk in PBS-T. Secondary peroxidase-couple antibodies (Dako-Agilent) were incubated at RT for 1 h. ECL-based chemiluminescence was detected using Pierce ECL Western Blotting Substrate (Thermo Scientific) and Hyperfilms (GE). Autoanti-dsDNA was deposited to the DSHB by Voss, E.W. (DSHB Hybridoma Product autoanti-dsDNA).

Protein crystallization and data collection

Purified *E. coli* C7 DarT1^{E152A} protein was concentrated to 26.9 mg/mL and incubated for co-crystallization with substrates used at following concentrations: 4 mM β-NAD$^+$, 4 mM carba-NAD$^+$, 1.56 mM (1.5x) ssDNA of sequence AAGAC. For co-crystallization with NAD$^+$ ligands and unmodified DNA, proteins were first pre-incubated with 4 mM β-NAD$^+$ or carba-NAD$^+$ for 30 min at 4°C which was followed by incubation with DNA for another 30 min.

P. nicotianae var. *parasitica* NADAR protein was concentrated 9.5 mg/mL and incubated with 4 mM ADP-ribose for 30 min at 4°C. *G. lovleyi* NADAR protein was concentrated to 25.5 mg/mL for crystallization trials. Crystallization trials were performed with *E. coli* C7 DarT1^{E152A} at 4°C and with *P. nicotianae* var. *parasitica* and *G. lovleyi* NADAR at 20°C using the sitting-drop vapour-diffusion method. Crystallization drops were set-up in MRC two-well crystallization microplates (Swissci) using the Mosquito Crystal robot (TTP Labtech) with protein to reservoir ratios of 1:1 and 1:2 in 150 nL total volume equilibrated against 75 μL of reservoir solution.

Crystals of *E. coli* C7 DarT1^{E152A} protein in complex with β-NAD$^+$ were obtained with reservoir solution containing 4.0 M sodium formate and 0.1 M Sodium acetate pH 5.5 while *E. coli* C7 DarT1^{E152A} protein in complex with carba-NAD$^+$ and ADPr-DNA (i.e. β-NAD$^+$/ssDNA) crystallised with reservoir solution containing 4.0 M sodium formate and 0.1 M TRIS-Cl pH 7.5. Crystals of *G. lovleyi* NADAR protein grew in 0.15 M potassium thiocyanate, 0.1 M TRIS-Cl pH 7.5, 18% (*w/v*) PEG 5000 MME and crystals of *P. nicotianae* var. *parasitica* NADAR protein in complex with ADP-ribose appeared in presence of 0.1 M sodium HEPES pH 7.5 and 20% (*w/v*) PEG 10000. Crystals were harvested using reservoir solution supplemented with 20% ethylene glycol (*v/v*) as a cryo-protectant prior to flash freezing in liquid nitrogen. X-ray data were collected at beamline I03 at the Diamond Light Source (Rutherford Appleton Laboratory, Harwell, UK) and data collection statistics are given in Table S1.

QUANTIFICATION AND STATISTICAL ANALYSIS

X-ray data were processed using the XIA2-DIALS platform[49] and phase information was obtained using the molecular replacement method with PHASER[50] using AlphaFold2[48] models of *E. coli* C7 DarT1 and *G. lovleyi* and *P. nicotianae* var. *parasitica* NADAR. Density modification was implemented with PARROT.[57] Atomic models were improved following consecutive cycles of manual building in COOT[51] and structure refinement in REFMAC5.[52] The structures were refined to good Ramachandran statistics and MolProbity[58] was used to validate the models prior to deposition in the PDB. Processing and refinement statistics are given in Table S1. Structural alignments and analyses, as well as figure preparation, were carried out using PyMol (Molecular Graphics System, Version 2.3.3 Schrödinger, LLC). For multiple-sequence alignments, JalView v2[53] and MAFFT7[59] was used. The phylogenetic tree for the NADAR superfamily was generated with SplitsTree4[54] (v4.15.1) using the Neighbour-Joining (NJ) method[60] and confidence levels estimated using 1000 cycles of the bootstrap method. The NCBI accession IDs of NADAR sequences are listed in Table S2. PSIPRED 4.0[55] was used for secondary structure prediction. Sequence conservation mapping was performed using ConSurf 2016.[56] Inkscape 1.2.1 was used for figure preparation. The number of repeats performed of a respective experiment is provided in the figure legends.

Molecular Cell, Volume *83*

Supplemental information

Molecular basis for the reversible ADP-ribosylation of guanosine bases

Marion Schuller, Roberto Raggiaschi, Petra Mikolcevic, Johannes G.M. Rack, Antonio Ariza, YuGeng Zhang, Raphael Ledermann, Christoph Tang, Andreja Mikoc, and Ivan Ahel

Molecular basis for the reversible ADP-ribosylation of guanosine bases

Marion Schuller[1], Roberto Raggiaschi[1], Petra Mikolcevic[2], Johannes G. M. Rack[1], Antonio Ariza[3], YuGeng Zhang[1], Raphael Ledermann[4], Christoph Tang[1], Andreja Mikoc[2], Ivan Ahel[1*]

[1]*Sir William Dunn School of Pathology, University of Oxford, Oxford, United Kingdom*
[2]*Division of Molecular Biology, Ruđer Bošković Institute, Zagreb, Croatia*
[3]*School of Biosciences, University of Sheffield, Sheffield, United Kingdom*
[4]*Department of Biology, University of Oxford, Oxford, United Kingdom*

*Corresponding author and lead contact: ivan.ahel@path.ox.ac.uk

SUPPLEMENTAL INFORMATION

SUPPLEMENTARY FIGURES

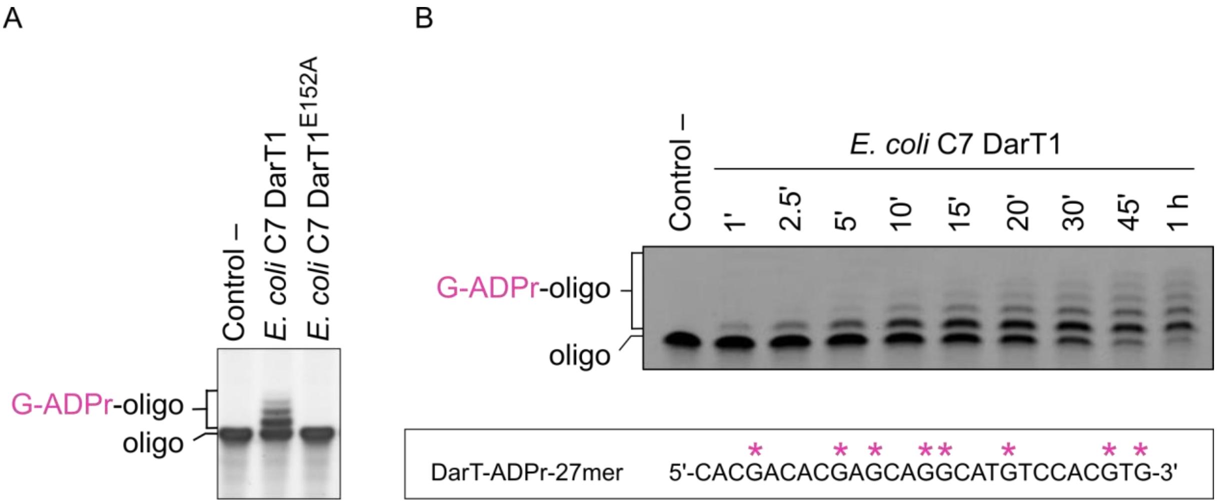

Supplementary Figure S1. ADP-ribosylation activity of *E. coli* C7 DarT1, related to Figure 2. (A) *In vitro* ADP-ribosylation activity of *E. coli* C7 DarT1 wild-type compared to mutant on the ssDNA substrate "DarT-ADPr-27mer". Several distinct shifts of modified oligo compared to the unmodified oligo can be visualised, indicating the presence of multiple ADPr modifications on the substrate. Representative of three independent experiments. **(B)** *In vitro* ADP-ribosylation activity of *E. coli* C7 DarT1 on the substrate "DarT-ADPr-27mer" over a time course of 1h. Several ADP-ribose modifications are added onto the substrate over time leading to a laddering effect. Representative for three independent experiments.

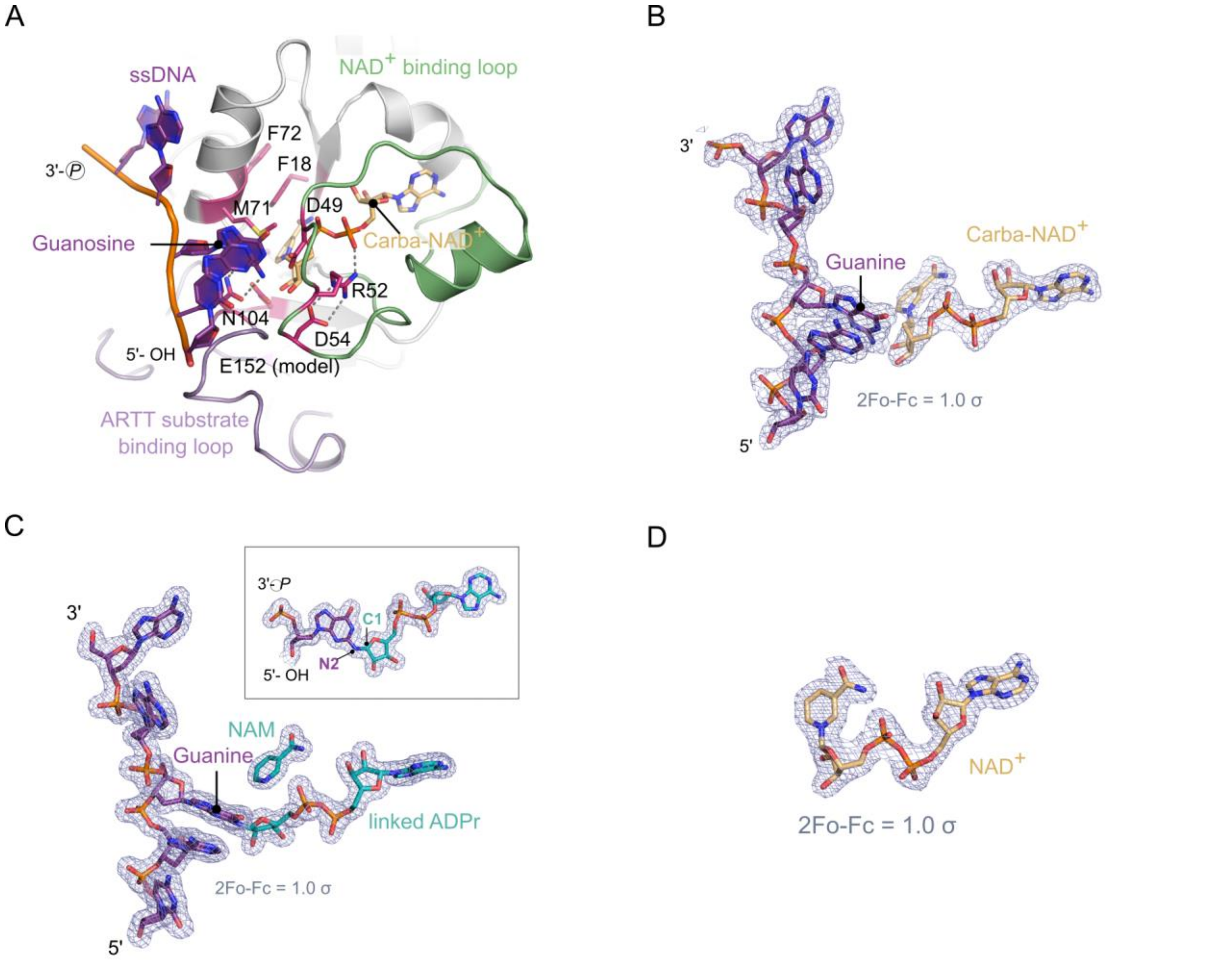

Supplementary Figure S2. DarT1 in substrate-bound pre-and post-reaction states, related to Figure 3. (A) Cartoon-stick model of the co-crystal structure of *E. coli* C7 DarT1^{E152A} with carba-NAD^{+} (brown sticks) and ssDNA (sequence AAGAC). The substrate-binding ARTT loop is highlighted in purple, and the NAD^{+}-binding loop is in green. Active site residues are shown as pink sticks. **(B)** The 2Fo-Fc electron density map contoured at 1.0 σ around the ssDNA and the carba-NAD^{+} ligand as in the structure shown in (A) is displayed in grey. **(C)** The 2Fo-Fc electron density map contoured at 1.0 σ around the ADP-ribosylated DNA and the NAM ligand as in *E. coli* C7 DarT1^{E152A} co-crystallised with NAD^{+} and DNA is displayed in grey. The resolution of 1.63 Å allows revealing the DarT1-established connection of the distal-ribose C1 atom to the guanine N2 atom. The rectangular inset shows an enlarged view of this ADPr – DNA linkage. **(D)** The 2Fo-Fc electron density map contoured at 1.0 σ around the NAD^{+} ligand as in the DarT1-NAD^{+} co-crystal structure is shown in grey.

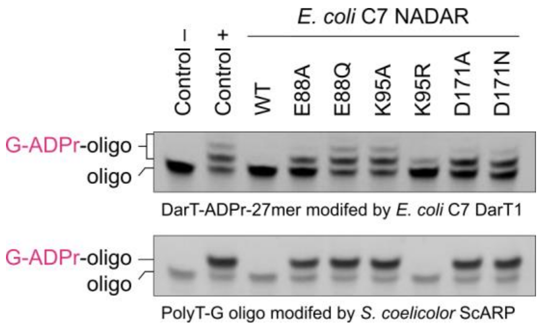

Supplementary Figure S3. Characterisation of catalytic residues of *E. coli* C7 NADAR, related to Figure 5. *In vitro* guanine-ADPr hydrolytic activity of *E. coli* C7 NADAR wild-type compared to catalytic mutants on the ssDNA substrates “DarT-ADPr-27mer” (modified by *E. coli* C7 DarT1) and “PolyT-G” (modified by *S. coelicolor* ScARP). Note, the de-ADP-ribosylation reaction of NADAR enzymes was only allowed to proceed for 15 min. Representative for three independent experiments.

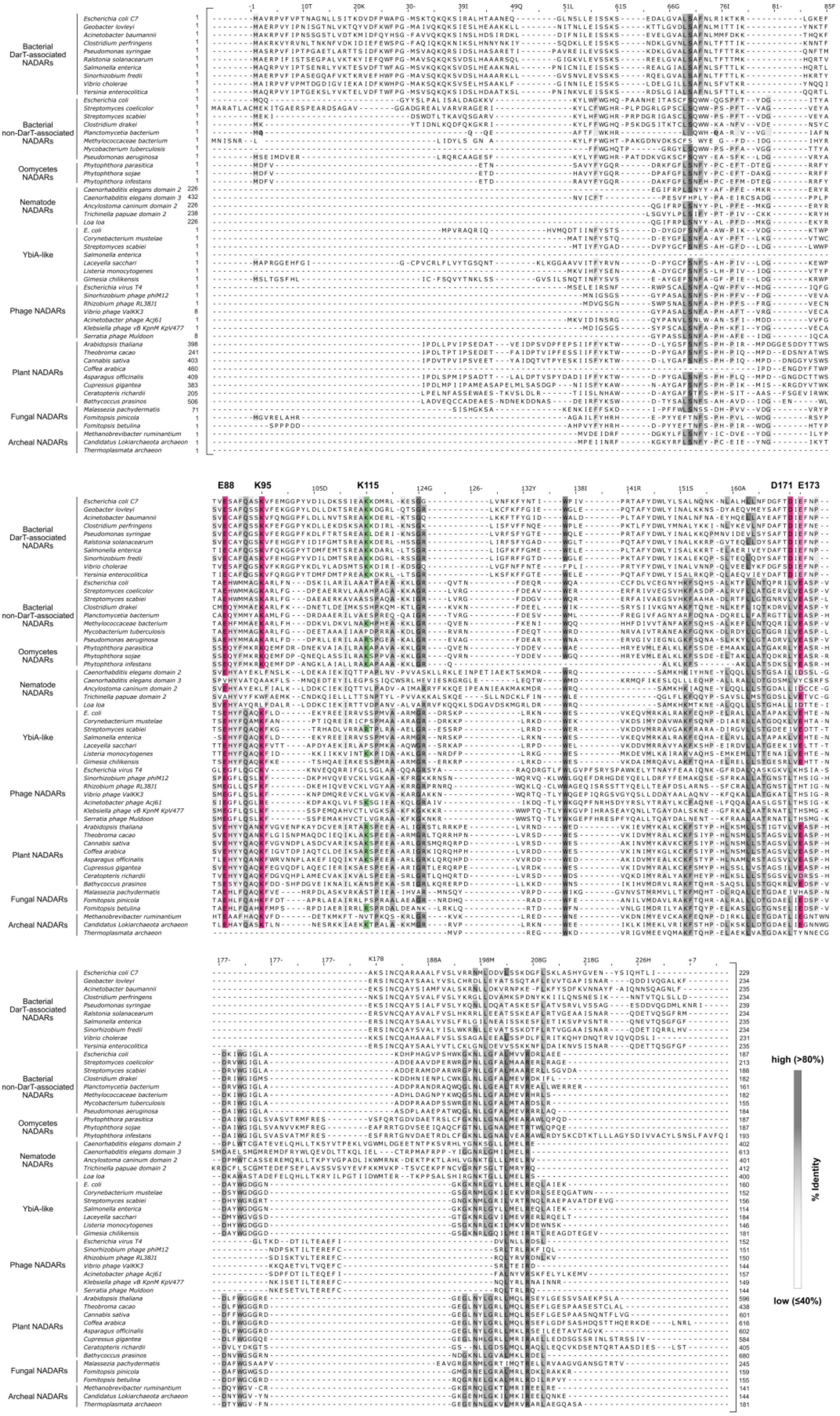

Supplementary Figure S4. Multiple sequence alignment of selected members of the NADAR superfamily, related to Figures 1 and 5. Catalytically relevant residues in *E. coli* C7 NADAR are highlighted in pink, and residues specific for DarT1-associated NADARs are highlighted in green. The alignment was used to construct the phylogenetic tree shown in Figure 1E.

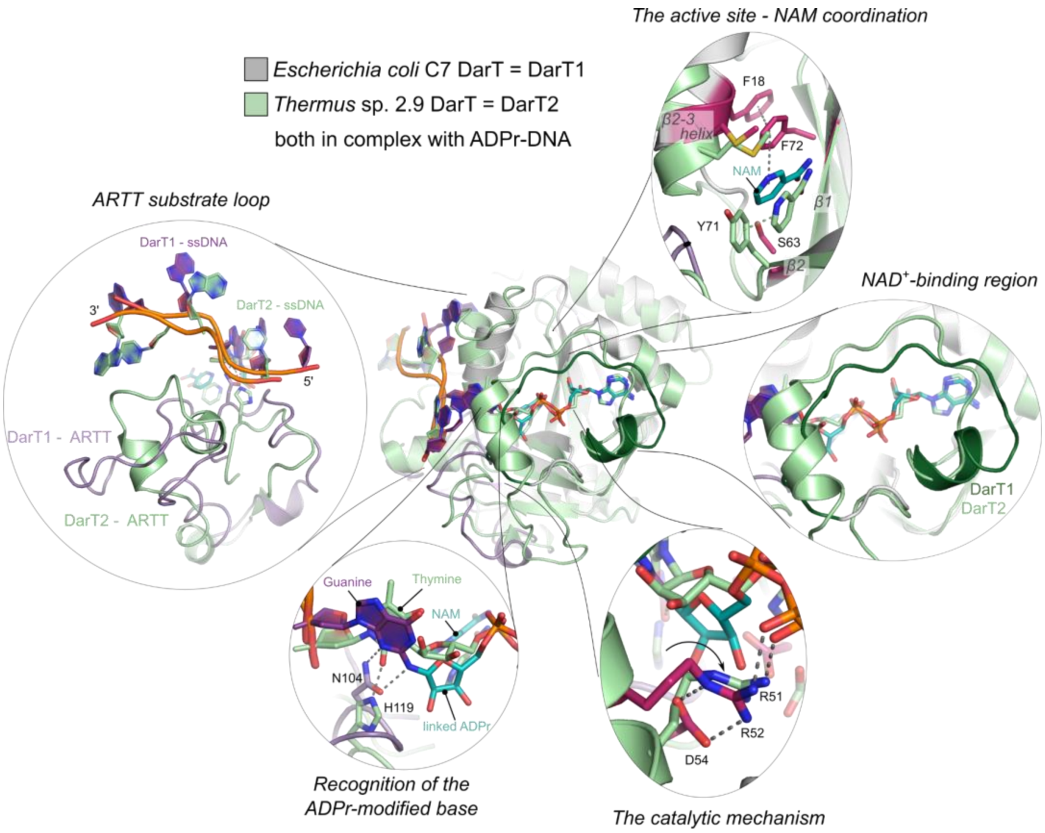

Supplementary Figure S5. Structural comparison of DarT1 and DarT2, related to Figure 3. In the middle of the figure, an overlay of *E. coli* C7 DarT, i.e. DarT1, with *Thermus* sp. 2.9 DarT, i.e. DarT2, both in complex with ADPr-DNA is shown. Differences in structural elements influencing the different catalytic functions are highlighted in the surrounding panels. The R51 side-chain flip observed in DarT2 compared to the pre-reaction state (not shown) is indicated by the black arrow. NAM coordination is relevant for considering NAD^+ polarisation.

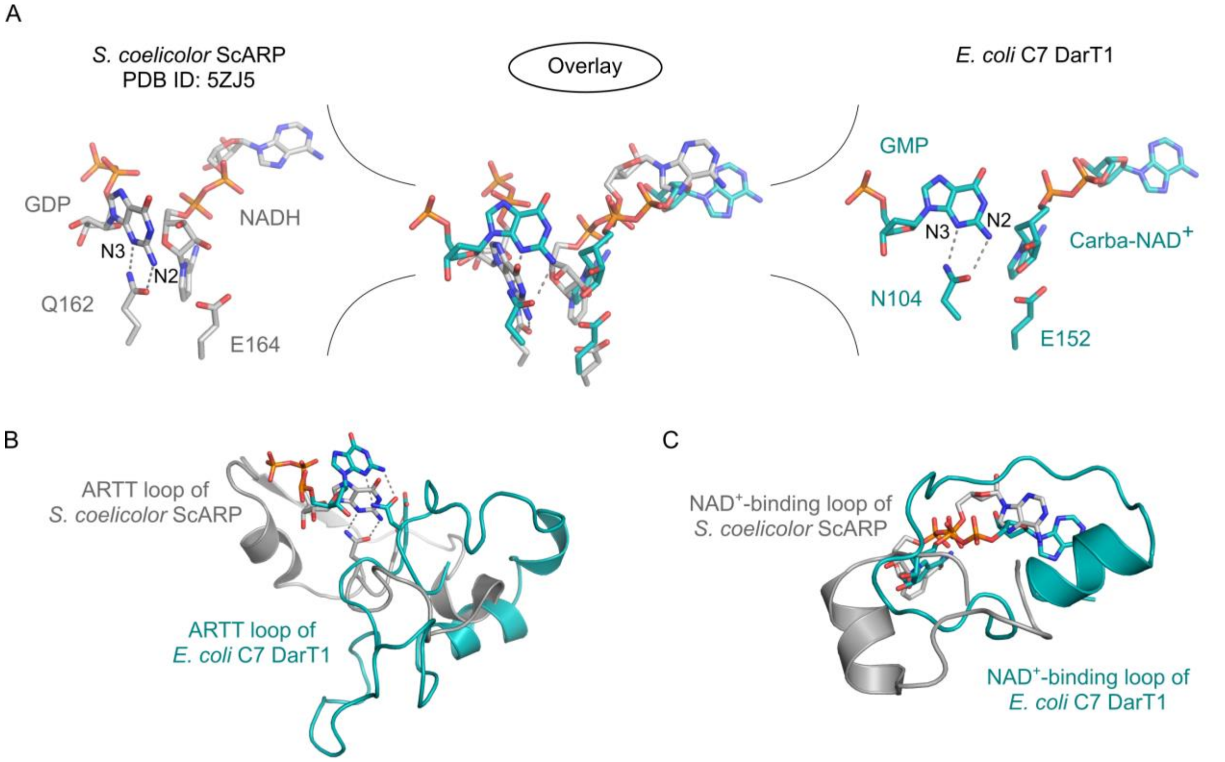

Supplementary Figure S6. Structural comparison of DarT1 and ScARP, related to Figure 6. (A) The co-crystal structure of *S. coelicolor* ScARP with GDP and NADH (PDB 5ZJ5) was overlaid with the *E. coli* C7 DarT1 structure in complex with GMP and carba-NAD$^+$. The NAD$^+$ derivatives and guanosine substrates take spatially similar positions although the transferases differ in their overall structural makeup, in particular regarding the ARTT substrate recognition loop **(B)** and the NAD$^+$-binding loop **(C)**. ScARP and DarT1 share the way of guanine positioning through N2 and N3 recognition and the orientation of the transferase-characteristic glutamate with respect to the distal-ribose of the NAD$^+$-derived ligand.

SUPPLEMENTARY TABLES

Supplementary Table S1. Data collection and refinement statistics for crystal structures described in this study, related to Figures 3, 4 and S2.

	E. coli C7 **DarT1: NAD$^+$**	***E. coli C7*** **DarT1: ADP-ribosylated DNA**	***E. coli*** **C7 DarT1: Carba-NAD$^+$ and DNA**	***G. lovleyi*** **NADAR apo**
**PDB accession code**	**8BAQ**	**8BAR**	**8BAS**	**8BAT**
**Data Collection**				
Synchrotron/beam line	DLS/I03	DLS/I03	DLS/I03	DLS/I03
Wavelength (Å)	0.9763	0.9763	0.9763	0.9763
Space group	$P\ 3_1\ 2\ 1$	$P\ 4_3\ 2_1\ 2$	$P\ 4_3\ 2_1\ 2$	$P\ 2_1\ 2\ 2_1$
a (Å)	62.31	61.76	61.90	39.28
b (Å)	62.31	61.76	61.90	81.60
c (Å)	113,08	215.04	215.55	86.63
α (°)	90.00	90.00	90.00	90.00
β (°)	90.00	90.00	90.00	90.00
γ (°)	120.00	90.00	90.00	90.00
Content of AU	1	1	1	1
Resolution (Å) [a]	53.96 - 2.00 (2.05 - 2.00)	61.74 - 1.63 (1.66 - 1.63)	61.90 – 1.92 (1.97 – 1.92)	59.40 – 2.30 (2.38 – 2.30)
R_{sym} (%) [a,b]	12.9 (140.4)	7.2 (158.1)	15.9 (236.3)	19.1 (147.5)
I/σ(I)	5.8 (0.7)	14.0 (1.3)	8.3 (0.9)	6.2 (1.0)
Completeness (%) [a]	99.9 (99.9)	100.0 (100.0)	100.0 (100.0)	100.0 (100.0)
Redundancy [a]	4.4 (4.4)	9.0 (9.0)	9.4 (9.8)	5.6 (5.3)
$CC_{1/2}$ (%) [a]	99.7 (61.5)	99.9 (70.1)	99.8 (50.1)	99.4 (55.7)
Unique reflections [a]	17764 (1267)	53204 (2567)	33191 (2151)	12984 (1242)
**Refinement**				
R_{cryst} (%) [c]	19.6	15.5	17.0	18.1
R_{free} (%) [d]	24.8	17.8	21.0	23.2
RMSD bond length (Å)	0.0043	0.012	0.013	0.011
RMSD bond angle (°)	1.171	1.89	2.04	1.95
Amino acids [e]	208 [47.6]	1721 [28.5]	1716 [39.1]	1761 [46.9]
Water [e]	131 [47.21]	356 [44.7]	242 [47.9]	124 [46.1]
Ligands [e]	5 [47.3]	173 [30.90]	177 [49.3]	4 [72.8]
Ions [e]	-	-	-	1 [49.7]
**Ramachandran plot**				
Favoured (%)	95.6	97.6	97.1	98.6
Allowed (%)	3.9	1.9	2,4	1.4
Disallowed (%)	0.5	0.5	0.5	0.0

(a) Data for the highest resolution shell are given in parentheses.
(b) R_{sym} = Σ|/-</>|/Σ/, where / is measured density for reflections with indices *hkl*.
(c) R_{cryst} = Σ||Fobs| - |Fcalc||/Σ|Fobs|.
(d) R_{free} has the same formula as R_{cryst}, except that calculation was made with the structure factors from the test set.
(e) Number of atoms followed the average B factor in brackets.

Supplementary Table S1. Data collection and refinement statistics for crystal structures described in this study, related to Figures 3, 4 and S2. (Continuation)

	***P. nicotianae var. parasitica* NADAR: ADPr**
**PDB accession code**	**8BAU**
**Data Collection**	
Synchrotron/beam line	DLS/I03
Wavelength (Å)	0.9763
Space group	$P\ 2_1\ 2_1\ 2_1$
a (Å)	44.83
b (Å)	66.59
c (Å)	72.89
α (°)	90.00
β (°)	90.00
γ (°)	90.00
Content of AU	1
Resolution (Å) [a]	49.17 - 1.60 (1.63 - 1.60)
R_{sym} (%) [a,b]	7.2 (212.8)
I/σ(I)	13.0 (0.9)
Completeness (%) [a]	100.0 (99.9)
Redundancy [a]	9.6 (9.8)
$CC_{1/2}$ (%) [a]	100.0 (59.6)
Unique reflections [a]	29538 (1421)
**Refinement**	
R_{cryst} (%) [c]	19.8
R_{free} (%) [d]	22.8
RMSD bond length (Å)	0.014
RMSD bond angle (°)	1.91
Amino acids	1539 [34.4]
Water	88 [36.6]
Ligands	56 [35.4]
Ions	-
**Ramachandran plot**	
Favoured (%)	98.4
Allowed (%)	1.6
Disallowed (%)	0.0

Supplementary Table S2. NCBI accession IDs of NADAR sequences used in this study, related to Figure 1.

Species	Accession number
**Bacterial NADARs (DarT-associated)**	
Escherichia coli C7	WP_032219797.1
Geobacter lovleyi [Trichlorobacter lovleyi]	WP_012470628.1
Sinorhizobium fredii	WP_014330845.1
Vibrio cholerae	WP_172778105.1
Acinetobacter baumannii	WP_001129309.1
Clostridium perfringens	MBS5923337.1
Yersinia enterocolitica	MBX9495195.1
Pseudomonas syringae	MCF9004830.1
Ralstonia solanacearum	WP_201016325.1
**Bacterial NADARs (non-DarT-associated)**	
Escherichia coli	WP_001183948.1
Methylococcaceae bacterium	NOQ36470.1
Pseudomonas aeruginosa	WP_116806626.1
Planctomycetia bacterium	MBL8863927.1
Streptomyces caniscabiei	WP_179201999.1
Streptomyces coelicolor	BDD75137.1
Mycobacterium tuberculosis	CNF61934.1
Clostridium drakei	WP_032077447.1
**Bacterial YbiA-like**	
Listeria monocytogenes	HAA3934926.1
Gimesia chilikensis	QDT22974.1
Laceyella sacchari	AUS10489.1
Salmonella enterica	WP_140040215.1
Streptomyces scabiei	WP_086756638.1
Corynebacterium mustelae	WP_047262349.1
Escherichia coli	HAW8155414.1
**Oomycetes NADARs**	
Phytophthora sojae	XP_009516941.1
Phytophthora nicotianae var. parasitica	XP_008911034.1
Phytophthora infestans	KAF4136563.1
**Phage NADARs**	
Sinorhizobium phage phiM12	YP_009143184.1
Acinetobacter phage Acj61	YP_004009822.1
Rhizobium phage RL38J1	QGZ13929.1
Klebsiella phage vB KpnM KpV477	YP_009288818.1
Vibrio phage ValKK3	YP_009201294.1
Escherichia virus T4	NP_049816.1
Serratia phage Muldoon	YP_009883850.1
**Nematode NADARs**	
Caenorhabditis elegans	NP_498348.1
Ancylostoma caninum	RCN47812.1
Loa loa	XP_003139559.1
Trichinella papuae	KRZ69666.1
**Plant and fungal NADARs**	
Fomitopsis pinicola	EPS93933.1
Fomitopsis betulina	KAI0715550.1
Arabidopsis thaliana	VYS59636.1
Theobroma cacao	EOY09810.1
Cannabis sativa	KAF4360218.1

Coffea arabica	XP_027089562.1
Asparagus officinalis	XP_020243908.1
Cupressus gigantea	ATG70670.1
Ceratopteris richardii	KAH7432347.1
Bathycoccus prasinos	XP_007514772.1
Malassezia pachydermatis	XP_017990970.1
**Archaeal NADARs**	
Methanobrevibacter ruminantium	WP_012956765.1
Candidatus Lokiarchaeota archaeon	MBD3226909.1
Thermoplasmata archaeon	MBE6519942.1

Supplementary Table S3. Oligonucleotides used in this study, related to STAR methods.

Oligo-ID	Sequence (5'→3')	Purpose
DarT_cryst	AAGAC	Co-crystallisation with DarT1
DarT-ADPr-27mer	CACGACACGAGCAGGCATGTCCACGTG	ADP-ribosylation activity assay
DarT-ADPr-27mer-rc	CACGTGGACATGCCTGCTCGTGTCGTG	Reverse complement for ADP-ribosylation activity assay
PolyT-G	TTTTTTGTTTTTTTTTTTT	ADP-ribosylation activity assay
PolyT-GG	TTTTTTGGTTTTTTTTTTT	ADP-ribosylation activity assay
PolyT-GTG	TTTTTTGTGTTTTTTTTTT	ADP-ribosylation activity assay
PolyT-GTTG	TTTTTTGTTGTTTTTTTTT	ADP-ribosylation activity assay
PolyT-GTTTG	TTTTTTGTTTGTTTTTTTT	ADP-ribosylation activity assay
PolyT-GTTTTG	TTTTTTGTTTTGTTTTTTT	ADP-ribosylation activity assay
DarT-Substrate_Motif1	CACTACACTATCATTCACTACCACTATC	ADP-ribosylation activity assay
DarT_Substrate_Motif2	CACTACACTATCATTCACGACCACTATC	ADP-ribosylation activity assay
DarT_Substrate_Motif3	CACTACACTATCATTCGAGCCCACTATC	ADP-ribosylation activity assay
DarT_Substrate_Motif4	CACTACACTATCATTCAGGCCCACTATC	ADP-ribosylation activity assay
DarT_Substrate_Motif5	CACTACACTATCATTCATGTCCACTATC	ADP-ribosylation activity assay
DarT_Substrate_Motif6	CACTACACTATCATTCACGTCCACTATC	ADP-ribosylation activity assay
DarT_Substrate_Motif7	CACTACACTATCATTCGTGTCCACTATC	ADP-ribosylation activity assay
EcoliDarT_fwd	AGAACCTGTACTTCCAATCCATGACCATCCAAGAAATTATTC	Cloning of *E. coli* DarT1 into pBAD33 expression vector by Gibson Assembly
EcoliDarT_rev	CCGCCAAAACAGCCAAGCTTTCAACCCAGATAATAATGAC	
GeoDarT_fwd	AGAACCTGTACTTCCAATCCATGCGTACCGCAGTTGAAAATC	Cloning of *G. lovleyi* DarT1 into pBAD33 expression vector by Gibson Assembly
GeoDarT_rev	CCGCCAAAACAGCCAAGCTTTCACAGCTGAAAGCTATTTGC	
pBAD33_fwd	AAGCTTGGCTGTTTTGGC	Vector amplification for Gibson Assembly
pBAD33_rev	GGATTGGAAGTACAGGTTC	
EcoliDarT-A152E-f001	CAGGCAGAGATTCTGGTGTTTGAGAAAATTCCGCCTAGCT	Mutagenesis of *E. coli* DarT1 to WT

EcoliDarT-A152E-r001	CAGAATCTCTGCCTGAACATCGGTGGTATATTCGCTCGGC	Mutagenesis of *E. coli* DarT1 to WT
GeoDarT-A152E-f001	CAGGCAGAGGTTCTGGTTTTTGGCACCATTGAACCGGCAT	Mutagenesis of *E. coli* DarT1 to WT
GeoDarT-A152E-r001	CAGAACCTCTGCCTGCGGATGCGTCGGATAACTACGCGGA	Mutagenesis of *E. coli* DarT1 to WT
EcoliDarT-F18A-f001	TTTCATGCGACCCATAGCGATAATCTGACCAGCATTCTGG	Mutagenesis of *E. coli* DarT1 to WT
EcoliDarT-F18A-r001	ATGGGTCGCATGAAACAGGCTGCGAATATTACGCTGCTGA	Mutagenesis of *E. coli* DarT1 to WT
EcoliDarT-D49A-f001	TGCAACGCGGAGGAACGCATTGATGGTCATCCTGATGCAA	Mutagenesis of *E. coli* DarT1
EcoliDarT-D49A-r001	TTCCTCCGCGTTGCAGTTATATTCATTGTTTTCGTTATCC	Mutagenesis of *E. coli* DarT1
EcoliDarT-R52A-f001	GAGGAAGCGATTGATGGTCATCCTGATGCAATTTGTCTGA	Mutagenesis of *E. coli* DarT1
EcoliDarT-R52A-r001	ATCAATCGCTTCCTCGTCGTTGCAGTTATATTCATTGTTT	Mutagenesis of *E. coli* DarT1
EcoliDarT-S63A-f001	TGTCTGGCGGTTAGCTATCCGAATGCCAAAATGTTTTACA	Mutagenesis of *E. coli* DarT1
EcoliDarT-S63A-r001	GCTAACCGCCAGACAAATTGCATCAGGATGACCATCAATG	Mutagenesis of *E. coli* DarT1
EcoliDarT-M71A-f001	GCCAAAGCGTTTTACAAATACCGCTGTCTGAAACCTGGTG	Mutagenesis of *E. coli* DarT1
EcoliDarT-M71A-r001	GTAAAACGCTTTGGCATTCGGATAGCTAACGCTCAGACAA	Mutagenesis of *E. coli* DarT1
EcoliDarT-F72A-f001	AAAATGGCGTACAAATACCGCTGTCTGAAACCTGGTGATT	Mutagenesis of *E. coli* DarT1
EcoliDarT-F72A-r001	TTTGTACGCCATTTTGGCATTCGGATAGCTAACGCTCAGA	Mutagenesis of *E. coli* DarT1
EcoliDarT-N104A-f001	CCGACCGCGGCAGCCAGCAATAATGTGCGTTTTATCAATC	Mutagenesis of *E. coli* DarT1
EcoliDarT-N104A-r001	GGCTGCCGCGGTCGGATAAAATGCACAATCTTTTGCCCAC	Mutagenesis of *E. coli* DarT1
EcoliDarT-D54A-f001	CGCATTGCGGGTCATCCTGATGCAATTTGTCTGAGCGTTAG	Mutagenesis of *E. coli* DarT1
EcoliDarT-D54A-r001	ATGACCCGCAATGCGTTCCTCGTCGTTGCAGTTATATTCA	Mutagenesis of *E. coli* DarT1
PpaNADAR_GTWY_for	GGGGACAAGTTTGTACAAAAAAGCAGGCTTCCTGGAAGTTCTG TTCCAGGGTCCGATGGACTTTGTGGAGACGAATTCTGCCG	Cloning Phytophthora NADAR
PpaNADAR_GTWY_rev	GGGGACCACTTTGTACAAGAAAGCT GGGTATTAGTCCTGTGGCTGTAACCAAGCGCG	
NADAR-EcoliC7-E88A-f001	ACCGTTGCGAGCGCGTTTCAAGCGAGCAAAGTGTTCGAAA	Mutagenesis of *E. coli* NADAR
NADAR-EcoliC7-E88A-r001	CGCGCTCGCAACGGTGAACTCCTTGCCCAGACGTTTGGTC	Mutagenesis of *E. coli* NADAR
NADAR-EcoliC7-K95A-f001	GCGAGCGCGGTGTTCGAAATGGGTGGCCCGTACGTTGACA	Mutagenesis of *E. coli* NADAR
NADAR-EcoliC7-K95A-r001	GAACACCGCGCTCGCTTGAAACGCGCTTTCAACGGTGAAC	Mutagenesis of *E. coli* NADAR
NADAR-EcoliC7-K115A-f001	GAGGCGGCGAAAGACATGCGTCTGAAGGAAAGCGGTGGCC	Mutagenesis of *E. coli* NADAR
NADAR-EcoliC7-K115A-r001	GTCTTTCGCCGCCTCAATGCTGCTTTTATCCAGGATGTCA	Mutagenesis of *E. coli* NADAR
NADAR-EcoliC7-K116A-f001	GCGAAGGCGGACATGCGTCTGAAGGAAAGCGGTGGCCTGG	Mutagenesis of *E. coli* NADAR
NADAR-EcoliC7-K116A-r001	CATGTCCGCCTTCGCCTCAATGCTGCTTTTATCCAGGATG	Mutagenesis of *E. coli* NADAR
NADAR-EcoliC7-R119A-f001	GACATGGCGCTGAAGGAAAGCGGTGGCCTGGTGAACTTCA	Mutagenesis of *E. coli* NADAR
NADAR-EcoliC7-R119A-r001	CTTCAGCGCCATGTCTTTCTTCGCCTCAATGCTGCTTTTA	Mutagenesis of *E. coli* NADAR
NADAR-EcoliC7-K121A-f001	CGTCTGGCGGAAAGCGGTGGCCTGGTGAACTTCAAATTTT	Mutagenesis of *E. coli* NADAR
NADAR-EcoliC7-K121A-r001	GCTTTCCGCCAGACGCATGTCTTTCTTCGCCTCAATGCTG	Mutagenesis of *E. coli* NADAR

NADAR-EcoliC7-D171A-f001	TTTACCGCGATCGAGTTTAACCCGGCGAAAAGCATTAACT	Mutagenesis of *E. coli* NADAR
NADAR-EcoliC7-D171A-r001	CTCGATCGCGGTAAAGCCGTCGAAGTTCAGCAGGTGCAGC	Mutagenesis of *E. coli* NADAR
NADAR-EcoliC7-E173A-f001	GATATCGCGTTTAACCCGGCGAAAAGCATTAACTGCCAAG	Mutagenesis of *E. coli* NADAR
NADAR-EcoliC7-E173A-r001	GTTAAACGCGATATCGGTAAAGCCGTCGAAGTTCAGCAGG	Mutagenesis of *E. coli* NADAR
NADAR-EcoliC7-K178A-f001	CCGGCGGCGAGCATTAACTGCCAAGCGCGTGCGGCGGCGC	Mutagenesis of *E. coli* NADAR
NADAR-EcoliC7-K178A-r001	AATGCTCGCCGCCGGGTTAAACTCGATATCGGTAAAGCCG	Mutagenesis of *E. coli* NADAR
NADAR-EcoliC7-D171N-f001	TTTACCAATATCGAGTTTAACCCGGCGAAAAGCATTAACT	Mutagenesis of *E. coli* NADAR
NADAR-EcoliC7-D171N-r001	CTCGATATTGGTAAAGCCGTCGAAGTTCAGCAGGTGCAGC	Mutagenesis of *E. coli* NADAR
NADAR-EcoliC7-E88Q-f001	ACCGTTCAGAGCGCGTTTCAAGCGAGCAAAGTGTTCGAAA	Mutagenesis of *E. coli* NADAR
NADAR-EcoliC7-E88Q-r001	CGCGCTCTGAACGGTGAACTCCTTGCCCAGACGTTTGGTC	Mutagenesis of *E. coli* NADAR
NADAR-EcoliC7-K95R-f001	GCGAGCCGCGTGTTCGAAATGGGTGGCCCGTACGTTGACA	Mutagenesis of *E. coli* NADAR
NADAR-EcoliC7-K95R-r001	GAACACGCGGCTCGCTTGAAACGCGCTTTCAACGGTGAAC	Mutagenesis of *E. coli* NADAR

Supplementary Table S4. Strains and plasmids used in this study, related to STAR methods.

Strain or plasmid-ID	Description	Source
DH5α	*huA2 a(argF-lacZ)U169 phoA glnV44 a80a(lacZ)M15 gyrA96 recA1 relA1 endA1 thi-1 hsdR17*	NEB
DH5α-macro	DH5α with integrated *T. aquaticus* DarG macrodomain at P21 site	Schuller *et al.*, 2021 [S1]
BL21	*fhuA2 [lon] ompT gal [dcm] ∆hsdS*	NEB
BL21(DE3)	*fhuA2 [lon] ompT gal (λ DE3) [dcm] ∆hsdS λ DE3 = λ sBamHIo ∆EcoRI-B int::(lacI::PlacUV5::T7 gene1) i21 ∆nin5*	NEB
Rosetta^{TM} BL21 (DE3)	*F-ompT hsdSB(rB- mB-) gal dcm (DE3) pRARE* (cam^R)	Novagen
**Plasmids**		
pBAD33	Medium copy plasmid with an arabinose-inducible promoter; cam^R	Guzman *et al.*, 1995 [S2]
pET28a	Medium copy plasmid containing the IPTG-inducible promoter; kan^R	Novagen
pNIC28-Bsa4	Medium copy plasmid containing the IPTG-inducible promoter; kan^R	Addgene [S3]
pBAD33_Taq_*darT*	pBAD33 carrying *T. aquaticus darT* full-length; cam^R	Jankevicius *et al.*, 2017 [S4]
pET28_SC_*SCO5461*	pET28a carrying *S. coelicolor scarp (SCO5461)* full-length; kan^R	Lalić, J. *et al.*, 2016 [S5]
pET28_Taq_*darG*_macro	pET28a carrying *T. aquaticus darG* macrodomain (aa 1-155); kan^R	Jankevicius *et al.*, 2017 [S4]
pBAD33_Ecoli_*darT1*	pBAD33 carrying *E. coli* C7 *darT1* full-length; cam^R	This study
pBAD33_Ecoli_*darT1*^{F18A}	pBAD33 carrying *E. coli* C7 *darT1*^{F18A} full-length; cam^R	This study
pBAD33_Ecoli_*darT1*^{D49A}	pBAD33 carrying *E. coli* C7 *darT1*^{D49A} full-length; cam^R	This study
pBAD33_Ecoli_*darT1*^{R52A}	pBAD33 carrying *E. coli* C7 *darT1*^{R52A} full-length; cam^R	This study
pBAD33_Ecoli_*darT1*^{D54A}	pBAD33 carrying *E. coli* C7 *darT1*^{D54A} full-length; cam^R	This study
pBAD33_Ecoli_*darT1*^{S63A}	pBAD33 carrying *E. coli* C7 *darT1*^{S63A} full-length; cam^R	This study
pBAD33_Ecoli_*darT1*^{M71A}	pBAD33 carrying *E. coli* C7 *darT1*^{M71A} full-length; cam^R	This study
pBAD33_Ecoli_*darT1*^{F72A}	pBAD33 carrying *E. coli* C7 *darT1*^{F72A} full-length; cam^R	This study
pBAD33_Ecoli_*darT1*^{N104A}	pBAD33 carrying *E. coli* C7 *darT1*^{N104A} full-length; cam^R	This study
pBAD33_Ecoli_*darT1*^{E152A}	pBAD33 carrying *E. coli* C7 *darT1*^{E152A} full-length; cam^R	This study
pNIC28_Ecoli_*darT*^{E152A}	pNIC28-Bsa4 carrying *E. coli C7 darT*^{E152A} full-length; kan^R	This study
pBAD33_Glov_*darT1*	pBAD33 carrying *G. lovleyi darT1* full-length; cam^R	This study
pBAD33_Glov_*darT1*^{E152A}	pBAD33 carrying *G. lovleyi darT1*^{E152A} full-length; cam^R	This study
pDEST17_Pnp_*nadar*	pDEST17 carrying *P. nicotianae* var. *parasitica nadar* full-length; kan^R	This study
pET28_Glov_*nadar*	pET28a carrying *G. lovleyi nadar* full-length; kan^R	This study
pET28_SinoR_*nadar*	pET28a carrying *S. fredii nadar* full-length; kan^R	This study
pET28_Ecoli_*nadar*	pET28a carrying *E. coli* C7 *nadar* full-length; kan^R	This study
pET28_Ecoli_*nadar*^{E88A}	pET28a carrying *E. coli* C7 *nadar*^{E88A} full-length; kan^R	This study
pET28_Ecoli_*nadar*^{E88Q}	pET28a carrying *E. coli* C7 *nadar*^{E88Q} full-length; kan^R	This study
pET28_Ecoli_*nadar*^{K95A}	pET28a carrying *E. coli* C7 *nadar*^{K95A} full-length; kan^R	This study
pET28_Ecoli_*nadar*^{K95R}	pET28a carrying *E. coli* C7 *nadar*^{K95R} full-length; kan^R	This study
pET28_Ecoli_*nadar*^{K115A}	pET28a carrying *E. coli* C7 *nadar*^{K115A} full-length; kan^R	This study
pET28_Ecoli_*nadar*^{K116A}	pET28a carrying *E. coli* C7 *nadar*^{K116A} full-length; kan^R	This study

pET28_Ecoli_*nadar*^{R119A}	pET28a carrying *E. coli* C7 *nadar*^{R119A} full-length; kan^{R}	This study
pET28_Ecoli_*nadar*^{K121A}	pET28a carrying *E. coli* C7 *nadar*^{K121A} full-length; kan^{R}	This study
pET28_Ecoli_*nadar*^{D171A}	pET28a carrying *E. coli* C7 *nadar*^{D171A} full-length; kan^{R}	This study
pET28_Ecoli_*nadar*^{D171N}	pET28a carrying *E. coli* C7 *nadar*^{D171N}full-length; kan^{R}	This study
pET28_Ecoli_*nadar*^{E173A}	pET28a carrying *E. coli* C7 *nadar*^{E173A} full-length; kan^{R}	This study
pET28_Ecoli_*nadar*^{K178A}	pET28a carrying *E. coli* C7 *nadar*^{K178A} full-length; kan^{R}	This study

SUPPLEMENTARY REFERENCES

S1. Schuller, M., Butler, R.E., Ariza, A., Tromans-Coia, C., Jankevicius, G., Claridge, T.D.W., Kendall, S.L., Goh, S., Stewart, G.R., and Ahel, I. (2021). Molecular basis for DarT ADP-ribosylation of a DNA base. Nature *596*, 597–602. 10.1038/s41586-021-03825-4.

S2. Guzman, L.M., Weiss, D.S., and Beckwith, J. (1997). Domain-swapping analysis of FtsI, FtsL, and FtsQ, bitopic membrane proteins essential for cell division in Escherichia coli. J Bacteriol *179*, 5094–5103. 10.1128/jb.179.16.5094-5103.1997.

S3. Savitsky, P., Bray, J., Cooper, C.D.O., Marsden, B.D., Mahajan, P., Burgess-Brown, N.A., and Gileadi, O. (2010). High-throughput production of human proteins for crystallization: The SGC experience. J Struct Biol *172*, 3–13. 10.1016/j.jsb.2010.06.008.

S4. Jankevicius, G., Ariza, A., Ahel, M., and Ahel, I. (2016). The toxin-antitoxin system DarTG catalyzes reversible ADP-ribosylation of DNA. Mol Cell *64*, 1109–1116. 10.1016/j.molcel.2016.11.014.

S5. Lalić, J., Marjanović, M.P., Palazzo, L., Perina, D., Sabljić, I., Žaja, R., Colby, T., Pleše, B., Halasz, M., Jankevicius, G., et al. (2016). Disruption of macrodomain protein SCO6735 increases antibiotic production in streptomyces coelicolor. Journal of Biological Chemistry *291*, 23175–23187. 10.1074/jbc.M116.721894.
